# Bifunctional Fluorophosphonium Triflates as Intramolecular Frustrated Lewis Pairs: Reversible CO_2_ Sequestration and Binding of Carbonyls, Nitriles and Acetylenes

**DOI:** 10.1002/chem.202102382

**Published:** 2021-08-25

**Authors:** Chun‐Xiang Guo, Kai Schwedtmann, Jannis Fidelius, Felix Hennersdorf, Arne Dickschat, Antonio Bauzá, Antonio Frontera, Jan J. Weigand

**Affiliations:** ^1^ Faculty of Chemistry and Food Chemistry TU Dresden Chair of Inorganic Molecular Chemistry 01062 Dresden Germany; ^2^ Department of Chemistry Universitat de Illes Balears 07122 Palma de Mallorca Spain

**Keywords:** carbon dioxide, frustrated Lewis pairs, fluorophosphonium cations, small-molecule activation

## Abstract

Electrophilic fluorophosphonium triflates bearing pyridyl (**3**[OTf]) or imidazolyl (**4**[OTf])‐substituents act as intramolecular frustrated Lewis pairs (FLPs) and reversibly form 1 : 1 adducts with CO_2_ (**5** ^+^ and **6** ^+^). An unusual and labile spirocyclic tetrahedral intermediate (**7** ^2+^) is observed in CO_2_‐pressurized (0.5–2.0 bar) solutions of cation **4** ^+^ at low temperatures, as demonstrated by variable‐temperature NMR studies, which were confirmed crystallographically. In addition, cations **3** ^+^ and **4** ^+^ actively bind carbonyls, nitriles and acetylenes by 1,3‐dipolar cycloaddition, as shown by selected examples.

## Graphical abstract

**Extending the FLP library**: Electrophilic fluorophosphonium triflates bearing pyridyl or imidazolyl substituents act as intramolecular N/P frustrated Lewis pairs that are capable of small molecule activation, that is, reversible CO_2_ sequestration and binding of carbonyls, nitriles and acetylenes.

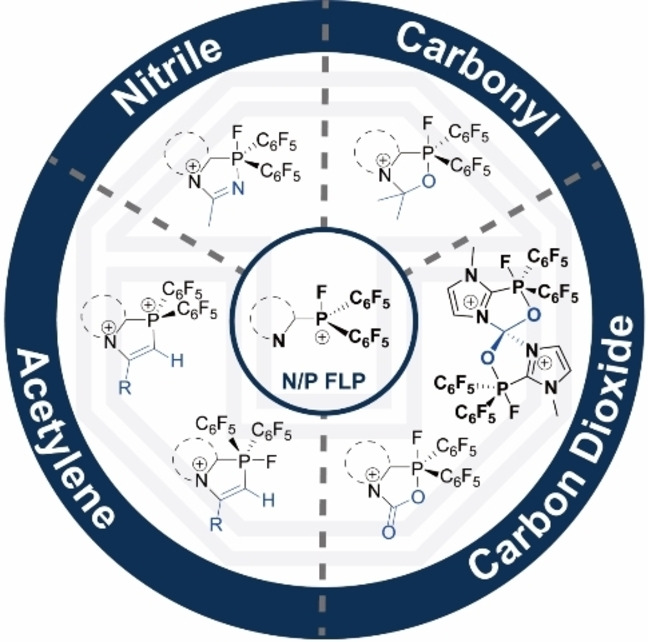

The selective, reversible binding of carbon dioxide (CO_2_) and its transformation into value‐added chemicals and materials is a challenge of today and success would significantly contribute to decline CO_2_ accumulation in the atmosphere.^[1]^ The chemistry of CO_2_ binding and transformation receives tremendous attention as it is regarded as an important heat‐trapping (greenhouse) gas^[2]^ but at the same time considered as readily available, abundant, nontoxic, and renewable C1 synthon in synthesis.^[3]^ In this context, generous efforts continue to be devoted to the development of efficient physical or chemical CO_2_ activation approaches.[^1a^, ^4^] A key‐step in the capture‐and‐release strategies and catalytic conversion, however, is a reversible binding of the CO_2_ molecule which needs to be enabled by low‐energy and readily‐cleavable bonds.^[5]^ Thus, a variety of organic carbon, nitrogen and phosphorus based Lewis bases were developed that are capable of forming adducts with the CO_2_ molecule.^[6]^ Notably, a variety of frustrated Lewis pairs (FLPs) have been investigated towards CO_2_ sequestration including inter‐ (I, II)^[7]^ and intramolecular (III,IV)^[8]^ FLPs derived from different combinations of electrophiles (e. g., borane‐, aluminum‐, or silylium‐based) and nucleophiles (e. g., phosphanes, carbenes, or amines; Scheme 1). However, the key step in a capture‐and‐release application for the conversion of CO_2_ ^[1]^ is usually hampered by the fact that the majority of FLPs form stable adducts with CO_2_.[^7a^, ^7b^, ^8^] Efforts towards binding of CO_2_ by highly Lewis acidic amidophosphoranes were reported by the Stephan group (Scheme 1, **V**).^[9]^ Although phosphonium cations have been extensively studied for their electrophilic properties, their application as Lewis acid in FLP chemistry is scarcely investigated so far. In thought of using fluorophosphonium cations in FLP chemistry, where in contrast to known systems the P atom is the Lewis acidic site, two different strategies are feasible: either the addition of a Lewis base to the fluorophosphonium salt or designing a fluorophosphonium derivative with an intramolecular Lewis basic site.

**Scheme 1:**
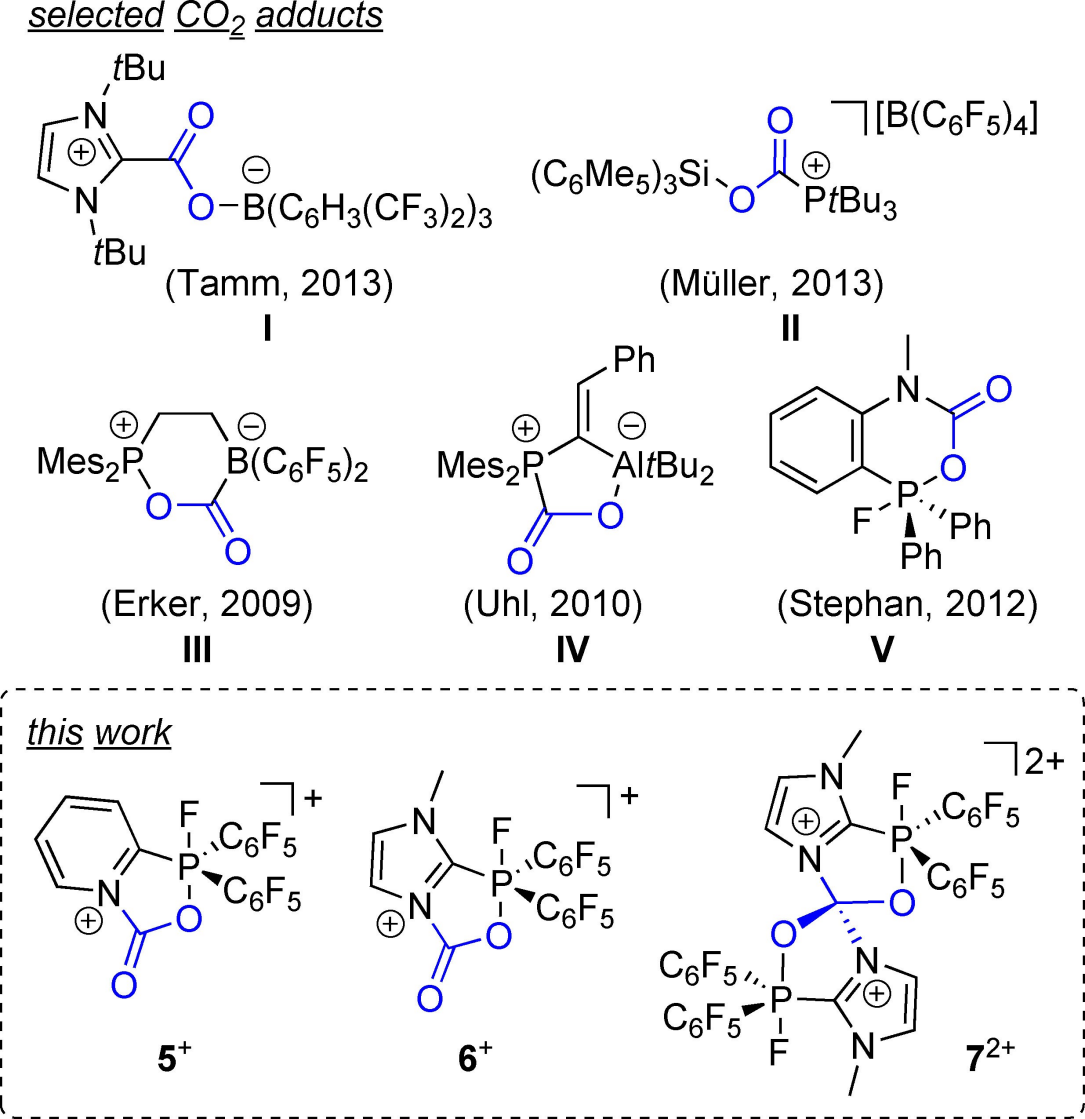
Selected examples of stable CO_2_ adducts of inter‐ and intramolecular FLP systems (**I**–**V**) and CO_2_ adducts **5** ^+^, **6** ^+^ and **7** ^2+^ derived from bifunctional fluorophosphonium compounds (this work).

Herein, we present the synthesis of pyridyl‐ and imidazolyl‐substituted fluorophosphonium triflate salts and their application as intramolecular N/P FLP systems. Both compounds readily form labile adducts with CO_2_ and bind carbonyls, nitriles and acetylenes by 1,3‐dipolar cycloaddition shown by selected examples.

To illustrate our approach, we first reacted fluorophosphonium salt **1**[OTf]^[10]^ as a very strong Lewis acid in combination with pyridine as a Lewis base and *p‐*tolylaldehyde in CH_2_Cl_2_ (Scheme 2).^[10]^ The formation of adduct **2** ^+^ is indicated by multinuclear NMR spectroscopy and the ^31^P NMR spectrum displays a doublet resonance at *δ*(P)=−53.2 ppm (^1^ *J*_PF_=644 Hz) being well in the range for penta‐coordinate P atoms. X‐ray analysis of **2**[OTf] confirms that the Lewis acidic P atom of the fluorophosphonium cation and Lewis basic N atom of the pyridine act cooperatively acts as an intermolecular FLP system activating the carbonyl group of the aldehyde (Figure 1). The corresponding P1−O1 and C1−N1 distances are 1.7038(10) Å and 1.5206(17) Å, respectively. The C1−O1 distance of 1.3974(16) Å is about 0.2 Å longer compared to the C=O double bond in free aldehydes.^[11]^ However, the application of this intermolecular FLP system is limited as we observed the decomposition of **1**[OTf] in the presence of pyridine without a suitable substrate.

**Scheme 2:**
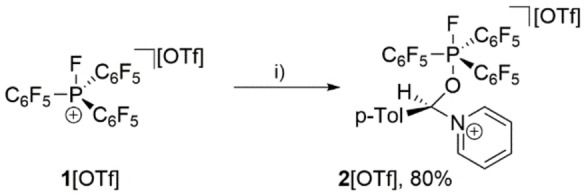
Adduct formation of **2**[OTf] from the reaction of **1**[OTf] with pyridine and *p*‐tolylaldehyde; i) pyridine (1 equiv.), *p*‐tolCHO (1 equiv.), CH_2_Cl_2_, RT, 4 h.

**Figure 1:**
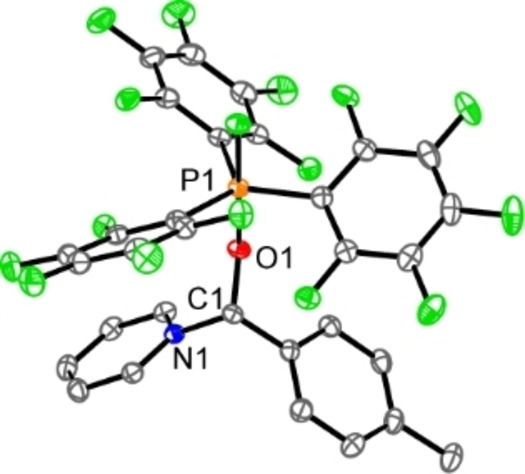
Molecular structure of **2**[OTf] ⋅ CH_2_Cl_2_ (hydrogen atoms, CH_2_Cl_2_, and non‐coordinating anions are omitted for clarity, ellipsoids are set at 50 % probability); Selected bond lengths (Å) and angles (°): P1−O1 1.7038(10), O1−C1 1.3974(16), C1−N1 1.5206(17).

We therefore targeted the synthesis of bifunctional fluorophosphonium salts such as **3**[OTf] and **4**[OTf] which contain a pyridyl‐ or imidazolyl‐substituent, respectively. The synthesis follows our recently reported one‐pot procedure, where *N*‐fluorobenzenesulfonimide (NFSI) is added to the corresponding phosphane (C_6_F_5_)_2_PR (R=imidazolyl, pyridyl), followed by the addition of MeOTf (Scheme 3).^[10]^ Triflate salts **3**, **4**[OTf] are isolated as colorless, air‐ and moisture‐sensitive salts in very good yields of >88 %.^[12]^ We started to investigate the cooperative properties of these salts by pressurizing degassed CD_2_Cl_2_ solutions with CO_2_ (2 bar). The corresponding ^31^P and ^19^F NMR spectra of the solution containing **3**[OTf] reveal no interaction or reaction at ambient temperature indicated by the resonances for cation **3** ^+^ (*δ*(^31^P)=54.8 ppm, *δ*(^19^F)=−114.0 ppm, ^1^ *J*_PF_=996 Hz) in the spectra. However, a VT NMR investigation disclosed the reversible and temperature‐dependent binding of CO_2_ by **3**[OTf] (Figure 2, left).

**Scheme 3:**
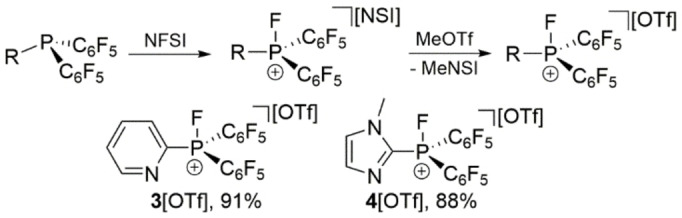
Synthesis of fluorophosphonium triflate salts **3**[OTf] and **4**[OTf]; i) +NFSI (1 equiv.), PhF, RT, 16 h; ii) +MeOTf (1 equiv.), RT, 4 h, −MeNSI; NSI^−^=[N(SO_2_Ph)_2_]^−^.

**Figure 2:**
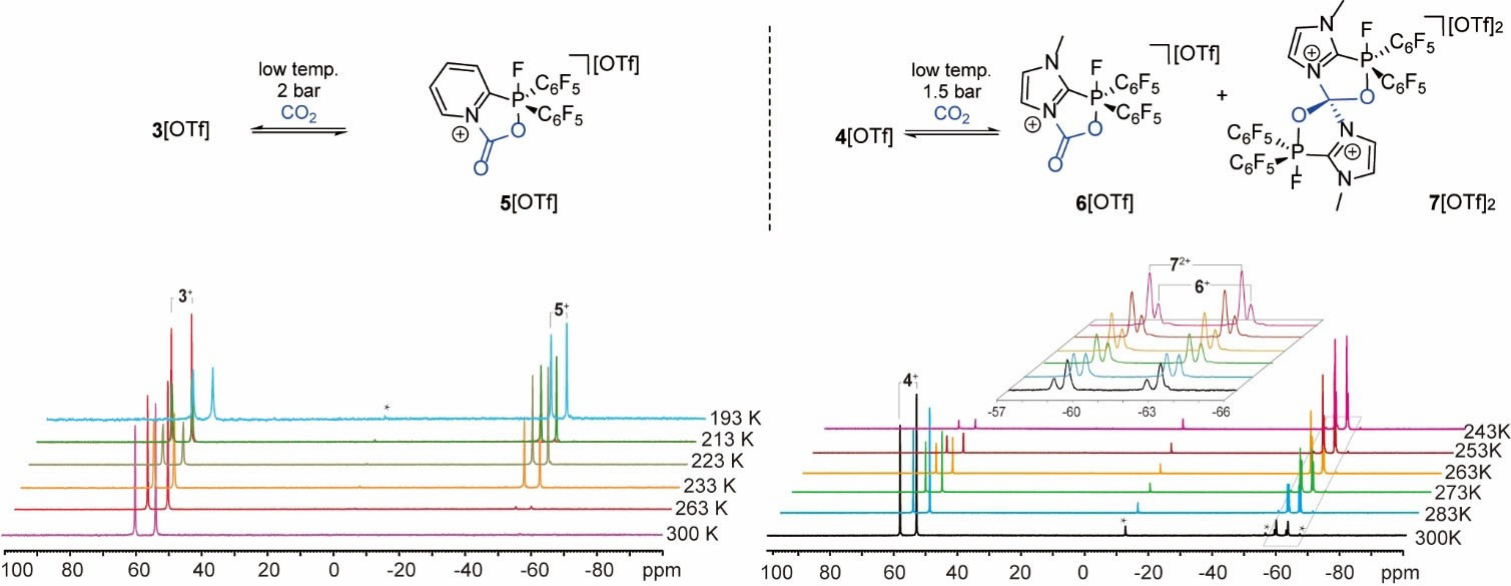
Equilibrium reaction of **3**[OTf] and **4**[OTf] with CO_2_ (top); variable‐temperature ^31^P NMR spectra of CO_2_ with **3**[OTf] (bottom left, 2 bar in CD_2_Cl_2_) and **4**[OTf] (bottom right, 1.5 bar in CD_3_NO_2_). Asterisks indicate small amounts of unidentified compounds.

The ^31^P NMR spectrum at 263 K shows, next to the dominant signal of the free cation **3** ^+^ (98 %), a small high field shifted doublet resonance at *δ*(^31^P)=−55.5 ppm (2 %, ^1^ *J*_PF_=769 Hz), which is well in the region for a penta‐coordinate phosphorus atom, thus, indicating the formation of the CO_2_ adduct **5**[OTf]. The corresponding resonance in the ^19^F NMR spectrum is detected as a downfield shifted doublet resonance at *δ*(^19^F)=−30.0 ppm. When the temperature is further decreased to 213 K the integral ratio of **5**[OTf] increases to 41 : 59. The carbon atom of the bound CO_2_ is observed as a doublet resonance at *δ*(^13^C)=141.8 ppm (^2^ *J*_PC_=3 Hz). Further decrease of the temperature to 193 K leads to the separation of a colorless precipitate, suggesting that **5**[OTf] exhibits a reduced solubility in CD_2_Cl_2_ at low temperature. Accordingly, gradually increase to RT leads to the dissolution of **5**[OTf] and its disappearance in the NMR spectrum. This process is reversible without decomposition of **3**[OTf]. We investigate this interaction of **3**[OTf] with CO_2_ theoretically with two different models (Figure 3). In **3** ^+^‐**CO_2_** _**A**, CO_2_ is activated by the incorporation of both the Lewis acidic and the Lewis basic site, whereas in model **3** ^+^‐**CO_2_** _**B** the activation exclusively proceeds through the Lewis acidic P atom. The CO_2_ binding energy is slightly stronger for model **3** ^+^ **‐CO_2_** _**A** (−20.4 kJ/mol) than model **3** ^+^‐**CO_2_** _**B** (−18.4 kJ/mol). Moreover, the calculated ^31^P NMR chemical shift change (Δ*δ*=*δ*(**3** ^+^‐**CO_2_** _**A**)−*δ*(**3** ^+^)) for model **3** ^+^‐**CO_2_** _**A** (Δ*δ*(^31^P)=−124 ppm) is in the range of the experimental result (Δ*δ*(^31^P)=−110.7 ppm), while the calculated Δ*δ*(^31^P) for model **3** ^+^‐**CO_2_** _**B** is only shifted by −17 ppm which is not observed in the respective ^31^P NMR spectrum. Thus, the CO_2_ activation is proposed to proceed via the P and pyridyl N atoms, affording **5**[OTf] in the configuration of model **3** ^+^‐**CO_2_** _**A**. As the DFT calculation reveals a slightly improved CO_2_ binding energy (−23.0 kJ/mol) for **4**[OTf], we also investigated the reactivity of **4**[OTf] towards CO_2_ (Figure 3). Pressurizing a degassed CH_2_Cl_2_ solution of **4**[OTf] with CO_2_ (0.5 bar) at ambient temperature results in the immediate formation of a colorless solid. However, the solid re‐dissolves immediately after releasing the pressure and all our separation attempts ended up with the recovery of **4**[OTf]. We therefore monitored the reaction of **4**[OTf] with CO_2_ (1.5 bar) by NMR spectroscopy in degassed CD_3_NO_2_. The ^31^P NMR spectrum at ambient temperature shows signals of two different adducts at *δ*(^31^P)=−60.0 ppm (^1^ *J*_PF_=737 Hz, 5 %) and *δ*(^31^P)=−61.5 ppm (^1^ *J*_PF_=740 Hz, 14 %) besides the signal of **4**[OTf] (*δ*(^31^P)=55.2 ppm, *δ*(^19^F)=−111.5 ppm, ^1^ *J*_PF_=1045 Hz, Figure 2, right). The corresponding signals in the ^19^F spectrum are detected at *δ*(^19^F)=−8.1 ppm (^1^ *J*_FP_=737 Hz) and *δ*(^19^F)=−16.9 ppm (^1^ *J*_FP_=740 Hz), respectively. Interestingly, the ^13^C NMR spectrum displays two signals for the bound CO_2_ moiety at *δ*(^13^C)=139.9 ppm (^2^ *J*_PC_=7 Hz) as a doublet resonance and at *δ*(^13^C)=105.5 ppm (^2^ *J*_PC_=8 Hz) as a triplet resonance suggesting a coupling to two phosphorus atoms.^13^ This strongly indicates the formation of a 1 : 1 FLP‐CO_2_ adduct **6**[OTf] (*δ*(^31^P)=−61.5 ppm, *δ*(^19^F)=−16.9 ppm, ^1^ *J*_PF_=740 Hz) and a 2 : 1 FLP‐CO_2_ adduct **7**[OTf]_2_ (*δ*(^31^P)=−60.0 ppm, *δ*(^19^F)=−8.1 ppm, ^1^ *J*_PF_=737 Hz). The ^31^P NMR chemical shift change of **6**[OTf] (Δ*δ*=*δ*(**6** ^+^)−*δ*(**4** ^+^), −116.7 ppm) is well in the range of calculated model **4** ^+^‐**CO_2_** _**A** (Δ*δ*(^31^P)=−108 ppm). Decreasing the temperature stepwise to 243 K, the integral ratio of **7**[OTf]_2_ gradually increases to 66 %, while the integral ratio of **6**[OTf] increases to 28 %. Variable‐temperature NMR studies at 0.5 or 2.0 bar CO_2_ pressure did not show significant difference on the integral ratio of **6**[OTf] and **7**[OTf]_2_. Although the calculated electrophilicities are similar for **3**[OTf] (GEI=3.164 eV, FIA=748.0 kJ/mol) and **4**[OTf] (GEI=3.121 eV, FIA=743.8 kJ/mol),^[10]^ in no case do we observe a double activation of CO_2_ with **3**[OTf]. This suggests that a certain nucleophilicity of the Lewis basic site is crucial for the formation of the 2 : 1 FLP‐CO_2_ adduct **7**[OTf]_2_.^[14]^

**Figure 3:**
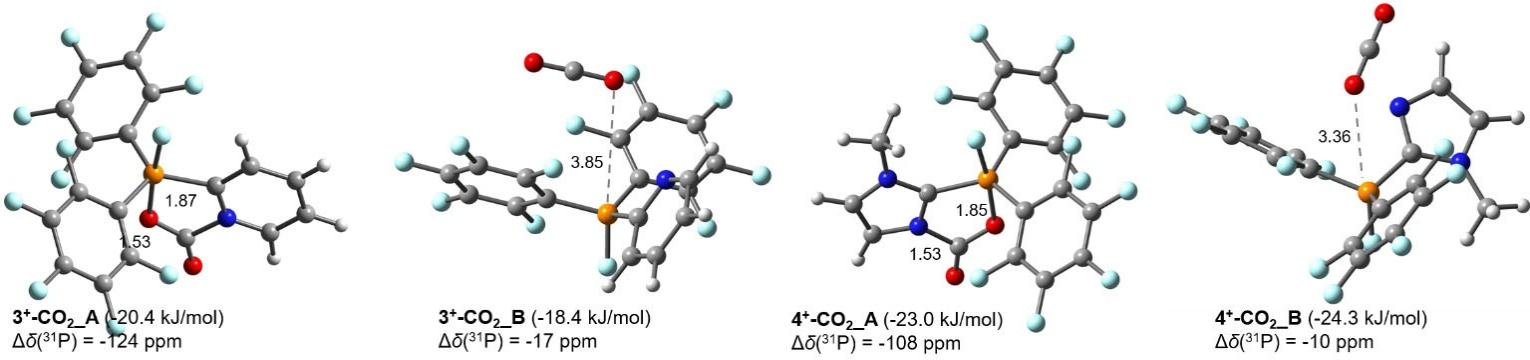
Optimized geometries and free energies of **3** ^+^‐ and **4** ^+^‐CO_2_ complexes (distances in Å, MP2/def2‐TZVP level of theory).

To our delight, we were able to obtain extremely sensitive but suitable co‐crystals containing both cations **6** ^+^ and **7** ^2+^ by vapor diffusion of CH_2_Cl_2_ into a CH_3_NO_2_ solution of **4**[OTf] under CO_2_ atmosphere (0.5 bar) at −30 °C (Figure 4). Although the double activation of CO_2_ has been reported with FLPs based on hafnium (**VI**) and aluminum (**VII**) complexes (Scheme 4),[^13^, ^15^] the tetrahedral cation **7** ^2+^ represents the only crystallographically characterized example of a metal‐free 2 : 1 FLP‐CO_2_ adduct.

**Figure 4:**
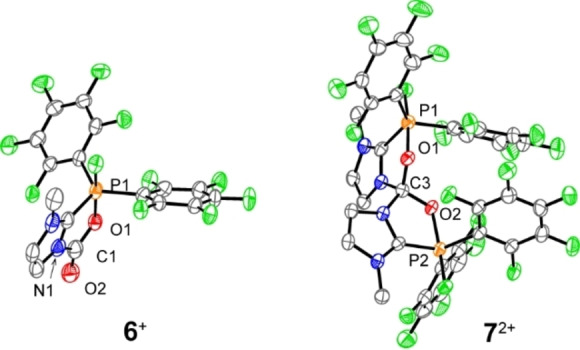
Molecular structure of co‐crystal **6**[OTf]⋅**7**[OTf]_2_⋅CH_3_NO_2_ (hydrogen atoms, CH_3_NO_2_, and non‐coordinating anions are omitted for clarity, ellipsoids are set at 50 % probability). Selected bond lengths (Å) and angles (°); for **6** ^+^: P1−O1 1.773(5), O1−C1 1.307(8), C1−N1 1.442(10), C1−O2 1.206(10), O1−C1−O2 128.1(7); for **7** ^2+^: P1−O1 1.771(4), P2−O2 1.744(4), O1−C3 1.352(7), O2−C3 1.366(6), O1−C3−O2 114.4(5).

**Scheme 4:**
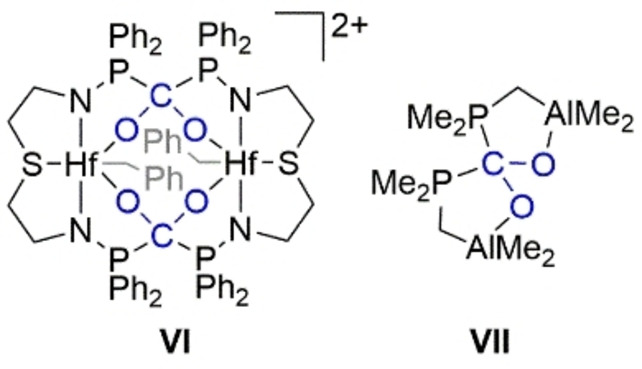
FLP‐CO_2_ adducts **VI** and **VII** containing a doubly activated CO_2_ molecule.

The molecular structure of **6** ^+^ which is consistent with the optimized structure **4^+^‐CO_2_** _**A**, shows that a CO fragment of the CO_2_ is integrated into a five‐membered PNC_2_O heterocycle while the other O atom is exocyclic. The CO_2_ moiety is bent, giving an O1−C1−O2 angle of 128.1(7)°. One oxygen is bound by the Lewis acidic P atom with a P1−O1 bond length of 1.773(5) Å and the carbon atom is stabilized by the Lewis basic N atom to give a N1−C1 bond length of 1.442(10) Å. The C1−O1 (1.307(8) Å) and C1=O2 (1.206(10) Å) bonds in the CO_2_ fragment are comparable to other FLP‐CO_2_ adducts (e. g., C−O 1.33 Å, C=O 1.21 Å).[^9a^, ^16^] In the case of the 2 : 1 FLP‐CO_2_ adduct **7** ^2+^, the CO_2_ molecule is captured by two cations **4** ^+^ resulting in a distorted tetrahedral geometry of the central carbon atom. The P−O bond lengths (P1−O1 1.771(4) Å, P2−O2 1.744(4) Å) are similar to those of **6** ^+^. The C−O bonds (O1−C3 1.352(7) Å, O2−C3 1.366(6) Å) are both significantly longer than those in **6** ^+^, but comparable to those reported for the hafnium based FLP‐CO_2_ adduct **VI** (1.383 and 1.369 Å, Figure 4).^[15]^

Inspired by these results, further studies concerned the 1,3‐dipolar cycloaddition of salts **3**,**4**[OTf] with carbonyls, nitriles, and acetylenes to the respective heterocycles (Scheme 5). At room temperature, the formation of acetone adducts **8 a**, **b**[OTf] (**8 a** ^+^/**8 b** ^+^: *δ*(^31^P)=−52.0/−60.0 ppm, *δ*(^19^F)=−6.5/9.6 ppm, ^1^ *J*_PF_=734/696 Hz, 97 and 84 % isolated yield) and acetonitrile adducts **9 a**, **b**[OTf] (**9 a^+^**/**9 b^+^**: *δ*(^31^P)=−49.3/−65.0 ppm, *δ*(^19^F)=3.0/19.9 ppm, ^1^ *J*_PF_=738/702 Hz, 71 and 95 % isolated yield) are indicated by multinuclear NMR spectroscopy (Table 1) and are isolated in good to excellent yields after workup. The carbonyl carbon atoms in **8 a**, **b**[OTf] (**8 a** ^+^/**8 b** ^+^: *δ*(^13^C)=101.8/96.7 ppm, ^2^ *J*_CP_=9/11 Hz) are significantly shifted to higher field compared to the free acetone (*δ*(^13^C)=207 ppm).^[17]^ The nitrile carbon atoms in **9 a**,**b**[OTf] are shifted to lower field (**9 a** ^+^/**9 b** ^+^: *δ*(^13^C)=151.0/150.2 ppm, ^2^ *J*_CP_=18/19 Hz). Interestingly, when compounds **9 a**, **b**[OTf] are reacted with acetone at room temperature, the formation of **8 a**, **b**[OTf] under release of CH_3_CN is observed after a reaction time of 1 h (Scheme 5, ii). The quantitative exchange is indicated by the clean shift of the corresponding doublet resonance in the ^31^P NMR spectrum of the reaction mixture.

**Scheme 5:**
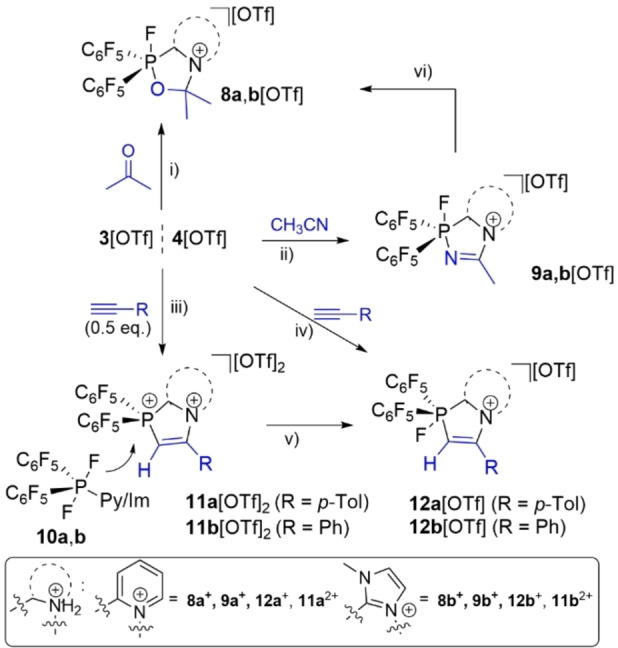
Reaction of **3**/**4**[OTf] with unsaturated compounds; i) +Me_2_CO (1 equiv.), CH_2_Cl_2_, RT, 1 h; ii) +MeCN (1 equiv.)_,_ CH_2_Cl_2_, RT, 1 h; iii) +acetylenes (0.5 equiv.), DCE, RT, 4 h; iv) +acetylenes (1 equiv.), DCE, 80 °C, 16 h; v) RT, 2 weeks, or 80 °C, 16 h, −**3**/**4**[OTf]; vi) +Me_2_CO (1 equiv.), −MeCN, CH_2_Cl_2_, RT, 1 h.

**Table 1:** Relevant ^19^F and ^31^P NMR data of the heterocycles **8**–**12**[OTf].

Compound^[a]^	Solvent	*δ*(^19^F) (ppm)	*δ*(^31^P) (ppm)	^1^ *J*_PF_ (Hz)
**8 a**[OTf]	CD_2_Cl_2_	−6.5	−52.0	734
**8 b**[OTf]	CD_2_Cl_2_	9.6	−61.0	696
**9 a**[OTf]	CD_3_CN	3.0	−49.3	738
**9 b**[OTf]	CD_3_NO_2_	19.9	−65.0	702
**11 a**[OTf]_2_	CD_3_CN	–	6.9	–
**11 a**[OTf]_2_	DCE^[a]^	–	−15.0	–
**12 a**[OTf]	DCE^[a]^	34.0	−88.8	707
**12 b**[OTf]	CD_2_Cl_2_	25.2	−98.8	718

[a] With C_6_D_6_ capillary.

When **3**, **4**[OTf] are reacted with acetylenes in dichloroethane (DCE) at room temperature for 4 h, the formation of **10 a**, **b** and **11 a**, **b**[OTf]_2_ is observed, while **12 a**, **b**[OTf] are cleanly formed by fluoride abstraction from **10 a**, **b** to **11 a**, **b** ^2+^ after reacting at 80 °C overnight or at ambient temperature for 2 weeks (Scheme 5, iii–v). Cation **11a** ^2+^(GEI=7.408 eV, FIA=1066.9 kJ/mol) is more Lewis acidic than **3** ^+^ (GEI=3.164 eV, FIA=748.0 kJ/mol), thus, low reaction tendency from **11 a**, **b** ^+^ to **12 a**, **b** ^+^ seems to be influenced by solvent effects, which significantly decrease the Lewis acidity compared to the gas phase calculations.[^10^, ^18^] **11 a**, **b**[OTf]_2_ are found as singlet resonances in the ^1^P NMR spectra at *δ*(^31^P)=6.9 and −15.0 ppm, while **12 a**, **b**[OTf] are observed as doublet resonances in the ^31^P and ^19^F NMR spectra (**12 a** ^+^/**12 b** ^+^: *δ*(^31^P)=−88.8/−98.8 ppm, *δ*(^19^F)=34.0/25.2 ppm, ^1^ *J*_PF_=707/718 Hz, Table 1). We were able to isolate **11 a**[OTf]_2_ ^[19]^ (78 %) and **12 b**[OTf] (66 %) as pure products for full characterization.

The molecular structures of compounds **8 a**, **b**[OTf], **9 a**, **b**[OTf] and **12 b**[OTf] are confirmed by X‐ray analysis of suitable single crystals which were obtained by slow vapor diffusion of *n*‐pentane into saturated CH_2_Cl_2_ solutions at −30 °C (Figure 5). All obtained structures show a distorted trigonal‐bipyramidal bonding environment at the P atom and display the expected five‐membered heterocycles with angle sum ranging from 536.8° to 540.0° (Table 2). In the structures of **8 a**, **b** ^+^ and **9 a**, **b** ^+^, the heteroatoms (O, N) and the fluorine atoms occupy the axial position. The P−F bonds (1.637(2) −1.673(13) Å) are elongated compared to those of the starting materials **3** ^+^ and **4** ^+^, but comparable to those in difluorophosphoranes (e. g., (C_6_F_5_)_3_PF_2_: 1.638(2) Å).^[20]^ The resulting P‐heteroatom distances in the acetone adducts (**8 a** ^+^/**8 b** ^+^: 1.695(2)/1.695(3) Å) are significantly shorter compared to the acetonitrile adducts (**9 a** ^+^/**9 b** ^+^: 1.777(2)/1.788(2) Å) while the C−N bonds are around 0.15 Å longer (**8 a** ^+^/**8 b** ^+^: 1.495(4) Å/1.411(5) Å, **9 a** ^+^/**9 b** ^+^: 1.250(3)/1.272(3) Å, Table 2).

**Figure 5:**
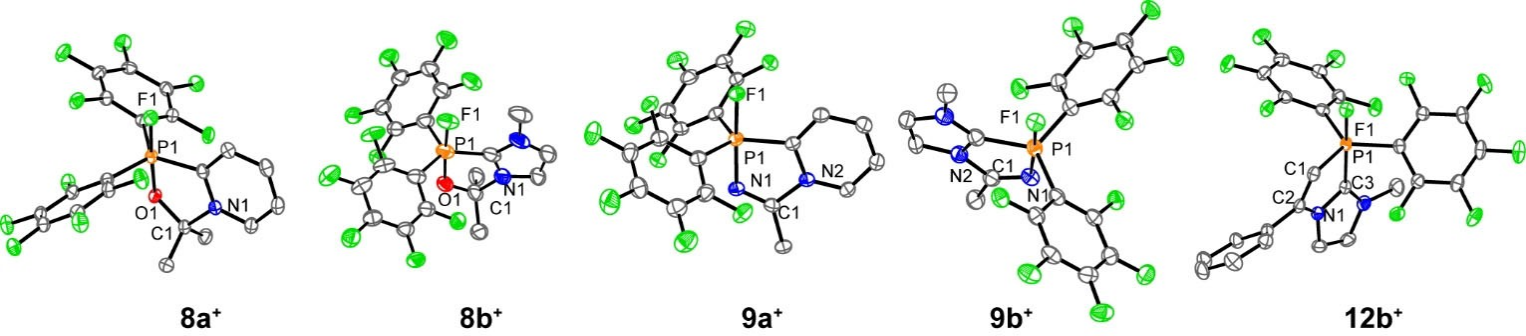
Molecular structures of selected FLP adducts (hydrogen atoms, solvent molecules, and non‐coordinating anions are omitted for clarity, ellipsoids are set at 50 % probability); selected structural parameters are included in Table 2.

**Table 2:** Selected geometrical parameters of the crystallographically characterized FLP adducts.

Compound	P−F (Å)	P−X^[a]^ (Å)	C−X^[a]^ (Å)	N−C (Å)	Angle sum^[b]^ (°)
**6**[OTf]	1.623(5)	1.773(5)	1.307(8)	1.442(10)	539.7
**8 a**[OTf]	1.673(13)	1.6989(15)	1.398(2)	1.519(3)	536.8
**8 b**[OTf]	1.637(2)	1.695(2)	1.411(5)	1.495(4)	537.2
**9 a**[OTf]	1.6427(16)	1.777(2)	1.250(3)	1.491(3)	540.0
**9 b**[OTf]	1.6426(16)	1.788(2)	1.272(3)	1.458(4)	539.9
**12 b**[OTf]	1.651(3)	1.773(5)	1.330(8)	1.415(6)	539.2

[a] X=O (**6** ^+^, **8 a**, **b** ^+^), N (**9 a**, **b** ^+^), C (**12 b** ^+^); [b] Angle sum of the newly formed five‐membered ring.

The C−O bond lengths of the carbonyl moiety **8 a** ^+^/**8 b** ^+^: 1.495(4)/1.411(5) Å and the C−N distances of the nitrile moiety (**9 a** ^+^/**9 b** ^+^: 1.250(3)/1.272(3) Å) are typical for C−O single bonds and C=N double bonds, respectively.^[11]^ The structure of **12 b** ^+^ shows that the pyridyl moiety is in the axial position, opposite to the fluorine atom (Figure 5). Thus, the terminal carbon atom of the alkyne occupies one of the equatorial positions adopting the minimum energy configuration due to steric restraints. The newly formed P1‐C1 bond distance (1.790(6) Å) and the N1−C2 bond distance (1.414(6) Å) are comparable to those in the acetonitrile adduct **9 b** ^+^ (Table 2). The C1−C2 bond length (1.330(8) Å) is well in the range of a typical C=C double bond.^[11]^

Deposition Numbers 2061970 (for **4**[OTf] ⋅ CH_2_Cl_2_), 2061971 (for **2**[OTf]), 2061972 (for **3**[OTf] ⋅ C_6_H_5_F), 2061973 (for **6**[OTf] ⋅ **7**[OTf]_2_ ⋅ CH_3_NO_2_), 2061974 (for **8 a**[OTf] ⋅ CH_2_Cl_2_), 2061975 (for **8 b**[OTf] ⋅ CH_2_Cl_2_), 2061976 (for **9 a**[OTf] ⋅ (CH_2_Cl_2_)_2_ ⋅ CH_3_CN), 2061977 (for **9 b**[OTf]), 2061978 (for **13**), 2061979 (for **12b**[OTf]), and 2061980 (for **14**) contain the supplementary crystallographic data for this paper. These data are provided free of charge by the joint Cambridge Crystallographic Data Centre and Fachinformationszentrum Karlsruhe Access Structures service.

In summary, electrophilic fluorophosphonium compounds bearing pyridyl (**3**[OTf]) or imidazolyl (**4**[OTf]) substituents, thus an additional Lewis basic site, represent a new type of intramolecular FLP that reacts with small molecules in a cooperative manner. The cyclizations of **3**[OTf] and **4**[OTf] with the 1,2‐dipolar compounds acetone, acetonitrile and acetylenes gave rise to the formation of the corresponding heterocyclic compounds, thus illustrating the cooperative reactivity of the new FLP derivatives. This is additionally demonstrated by the reversible formation of adducts with CO_2_ (**5** ^+^ and **6** ^+^) at low temperature, which was investigated by variable‐temperature NMR studies and X‐ray analysis. Surprisingly, the bifunctional phosphonium cation **4** ^+^ forms an adduct with CO_2_ (**7** ^2+^) comprising one molecule of CO_2_ and two molecules of **4** ^+^, resulting in a spirocyclic geometry at the central carbon atom, which is hitherto unreported for an N/P FLP system. These novel bifunctional phosphonium cations extend the diverse library of FLP systems, and the reversible CO_2_ adduct formation might provide new applications for in FLP chemistry, which we are currently investigating.

## Conflict of interest

The authors declare no conflict of interest.

## Supporting information

As a service to our authors and readers, this journal provides supporting information supplied by the authors. Such materials are peer reviewed and may be re‐organized for online delivery, but are not copy‐edited or typeset. Technical support issues arising from supporting information (other than missing files) should be addressed to the authors.

Supporting Information
