# Supplementary material for: Bifunctional Fluorophosphonium Triflates as Intramolecular Frustrated Lewis Pairs: Reversible CO2 Sequestration and Binding of Carbonyls, Nitriles and Acetylenes
Source: Chemistry. 2021 Aug 25;27(55):13709–14. doi: 10.1002/chem.202102382 (PMC8518062; doi:10.1002/chem.202102382)
Supplement: Supplementary file 1 — Supporting Information [file CHEM-27-13709-s001.pdf]

Chemistry–A European Journal

Supporting Information

Bifunctional Fluorophosphonium Triflates as Intramolecular Frustrated Lewis Pairs: Reversible CO_2 Sequestration and Binding of Carbonyls, Nitriles and Acetylenes

Chun-Xiang Guo, Kai Schwedtmann, Jannis Fidelius, Felix Hennersdorf, Arne Dickschat, Antonio Bauzá, Antonio Frontera, and Jan J. Weigand*

Content

1 Materials and Methods *2*
2 Preparation of heterocyclic substituted phosphanes *3*
3 Preparation of **3**-**4**[OTf] *5*
4 Synthesis of compound **2**[OTf] *6*
5 Reaction of **3**-**4**[OTf] with CO_2 .... *7*
6 Reaction of **3**-**4**[OTf] with dipolarophiles *9*
7 Crystallographic details *14*
8 Computational Methods *23*
9 Reference *30*
10 NMR spectra *32*

1 Materials and Methods

General Remarks: All manipulations are performed in a Glovebox (Pure lab HE GP-1SR) or using Schlenk techniques under an atmosphere of purified Nitrogen or Argon. Dry, oxygen-free solvents (CH_2Cl_2, CH_3CN, C_6H_5F, DCE (distilled from CaH_2), Et_2O (distilled from potassium/benzophenone) are employed. Deuterated benzene (C_6D_6) is purchased from Deutero and distilled from potassium. Anhydrous deuterated acetonitrile (CD_3CN), Nitromethane (CD_3NO_2), dichloromethane (CD_2Cl_2), and chloroform ($CDCl_3$) are purchased from Deutero, Sigma-Aldrich or Eurisotop. All distilled and deuterated solvents are stored over molecular sieves (4Å: CH_2Cl_2, Et_2O, *n*-pentane, C_6H_5F, C_6D_6, CD_2Cl_2, $CDCl_3$; 3Å: CH_3CN, CD_3CN, CD_3NO_2). All glassware is oven-dried at 160 °C prior to use. $(C_6F_5)_2PBr$[1], 2-(dichlorophosphaneyl)pyridine[2] and $[(C_6F_5)_3PF][OTf]$ (**1**[OTf])[3] are prepared according to reported procedures. All other chemicals are purchased by Sigma Aldrich, ABCR Chemicals, TCI, Manchester Organics or Acros Organics.

NMR spectra are measured on a Bruker AVANCE III HD Nanobay 400 MHz UltraShield (^{1}H (400.13 MHz), ^{13}C (100.61 MHz), ^{31}P (161.98 MHz), ^{19}F (376.50 MHz)), or on a Bruker AVANCE III HDX, 500 MHz Ascend (^{1}H (500.13 MHz), ^{13}C (125.75 MHz), ^{31}P (202.45 MHz) ^{19}F (470.59 MHz)). All ^{13}C NMR spectra are exclusively recorded with composite pulse decoupling. Reported numbers assigning atoms in the ^{13}C spectra are indirectly deduced from the cross-peaks in 2D correlation experiments (HMBC, HSQC). Chemical shifts are referenced to $\delta(Me_4Si)$ = 0.00 ppm (^{1}H, ^{13}C, externally), $\delta(CFCl_3)$ = 0.00 ppm (^{19}F, externally) and $\delta(H_3PO_4$, 85%) = 0.00 ppm (^{31}P, externally). Chemical shifts (δ) are reported in ppm. Coupling constants (J) are reported in Hz. Assignments of individual resonances are done using 2D techniques (HMBC, HSQC, HH-COSY) where necessary. ^{31}P NMR spectra are routinely recorded using a T_1 relexation delay of 5 seconds to ensure an adequate relaxation of the phosphorus nuclei and a reliable integrational ratio. Melting points are recorded on an electrothermal melting point apparatus (Büchi Switzerland, Melting point *M*-560) in sealed capillaries under Nitrogen atmosphere and are uncorrected. Infrared (IR) and Raman spectra are recorded at ambient temperature using a Bruker Vertex 70 instrument equipped with a RAM II module (Nd:YAG laser, 1064 nm). The Raman intensities are reported in percent relative to the most intense peak and are given in parenthesis. An ATR unit (diamond) is used for recording IR spectra. The intensities are reported relative to the most intense peak and are given in parenthesis using the following abbreviations: vw = very weak, w = weak, m = medium, s = strong, vs = very strong. Elemental analyses are performed on a Vario MICRO cube Elemental Analyzer by Elementar Analysatorsysteme 2 GmbH in CHNS modus.

2 Preparation of heterocyclic substituted phosphanes

2.1 Preparation of 13

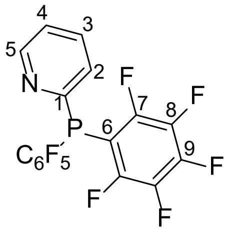

A freshly prepared solution of (C_6F_5)MgBr[1] (20 mmol) in Et_2O (30 ml) was added dropwise to a solution of 2-(dichlorophosphaneyl)pyridine[2] (1.80 g, 10 mmol) in Et_2O (10 mL) over a course of 30 min while maintaining the temperature at 0 °C. The resulting suspension was allowed to slowly warm up and react at ambient temperature overnight. After filtration through a pad of silica and washing with Et_2O (3 x 20 mL), all volatiles were removed *in vacuo*. The crude product was recrystallized from warm cyclohexane yielding **13** as an air-stable light yellow solid.

Yield: 1.64 g, 37%; **m.p.**: 100 – 101 °C; **Raman** (100 mW, 500 scans, 298 K, in cm^{-1}): 3059 (38), 1642 (100), 1574 (46), 1565 (13), 1393 (31), 1385 (6), 1277 (19), 1132 (22), 1044 (65), 990 (75), 834 (25), 720 (17), 586 (57), 506 (67), 447 (36), 391 (53), 328 (14), 283 (11), 240 (8), 224 (8), 203 (23); **IR** (ATR, 298 K, in cm^{-1}): 1641 (vw), 1573 (vw), 1514 (m), 1470 (vs), 1446 (m), 1425 (w), 1383 (w), 1286 (w), 1082 (s), 974 (vs), 835 (w), 773 (w), 754 (m), 729 (vw), 636 (w), 623 (vw); **^{1}H NMR** (CD_2Cl_2, 300K, in ppm): δ = 8.63 – 8.62 (1H, m, C5-H), 7.72 – 7.67 (1H, m, C3-H), 7.47 – 7.41 (1H, m, C2-H), 7.32 – 7.29 (1H, m, C4-H); **^{13}C{^{1}H} NMR** (CD_2Cl_2, 300K, in ppm): δ = 156.2 (d, $^1J_{CP}$ = 10 Hz, C1), 150.0 (d, $^2J_{CP}$ = 13 Hz, C5), 148.7 (dm, $^1J_{CF}$ = 249 Hz, C6), 143.5 (dm, $^1J_{CF}$ = 257 Hz, C8), 138.3 (dm, $^1J_{CF}$ = 253 Hz, C7), 136.8 (s, C3), 127.6 (d, $^2J_{CP}$ = 27.5 Hz, C2), 124.2 (s, C4), 107.5 (m, C9); **^{19}F NMR** (CD_2Cl_2, 300K, in ppm): δ = -128.5 (m, C7-F), -149.8 (m, C9-F), -161.1 (m, C8-F); **^{31}P{^{1}H} NMR** (CD_2Cl_2, 300K, in ppm): δ = 33.4 (m, P); **elemental analysis**: calculated for $C_{17}H_{14}F_{10}NP$: C 46.1, H 0.9, N 3.2, found: C 46.0, H 0.8, N 3.2; crystallographic data and details of the structure refinements are included in Table 7.2.1.

2.2 Preparation of 14

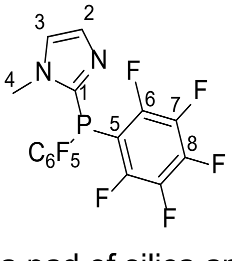

1-Methylimidazole (1.2 g, 15.0 mmol) was added to a solution of (C_6F_5)$_2$PBr (6.7 g, 15.0 mmol) in MeCN (20 mL). After stirring for 1 h at ambient temperature, Et_3N (2.3 g, 1.5 mL, 22.5 mmol) was added dropwise and the mixture was stirred for 16 h. After that all volatiles were removed *in vacuo*. The residue was suspended in Et_2O and filtered through a pad of silica and washed with Et_2O (3 x 20 mL). All volatiles of the filtrate were evaporated *in vacuo*. The crude product was recrystallized from warm cyclohexane yielding **14** as an air-stable colorless solid.

Yield: 3.0 g, 45%; **m.p.**: 96 – 97 °C; **Raman** (100 mW, 500 scans, 298 K, in cm^{-1}): 3147 (12), 3111 (16), 2959 (14), 1641 (54), 1504 (19), 1456 (6), 1416 (14), 1398 (23), 1382 (10), 1358 (21), 1344 (100), 1281 (20), 1144 (5), 1121 (12), 1081 (25), 918 (17), 846 (7), 831 (16), 754 (5), 700 (8), 684 (21), 636 (5), 588 (54), 513 (62), 483 (25), 445 (38), 415 (12), 391 (48), 374 (10), 336 (7), 283 (7), 251 (5), 236 (7); **IR** (ATR, 298 K, in cm^{-1}): 1640 (w), 1512 (s), 1471 (vs), 1415 (w), 1396 (w), 1381 (m), 1287 (w), 1280 (m), 1085 (vs), 1019 (w), 974 (vs), 917 (w), 845 (w), 831 (w), 752 (s), 727 (w), 698 (m), 683 (w), 635 (m), 623 (w), 511 (s), 481 (m), 439 (s), 413 (m); **^{1}H NMR** (CD_3CN, 300K, in ppm): δ = 7.28 (1H, d, $^{4}J_{HP}$ = 2.1 Hz, C2-H), 7.09 (1H, s, C3-H), 3.76 (3H, s, C4-H); **^{13}C{^{1}H} NMR** (CD_3CN, 300K, in ppm): δ = 148.8 (dm, $^{1}J_{CF}$ = 247 Hz, C6), 142.9 (dm, $^{1}J_{CF}$ = 255 Hz, C8), 138.8 (dm, $^{1}J_{CF}$ = 251.1 Hz, C7), 137.5 (dm, $^{1}J_{CP}$ = 11 Hz, C1), 132.2 (d, $^{3}J_{CP}$ = 4 Hz, C3), 127.3 (d, $^{3}J_{CP}$ = 2 Hz, C2), 107.4 (m, $^{1}J_{CP}$ = 27 Hz, C5), 34.8 (d, $^{3}J_{CP}$ = 13 Hz, C4);**^{19}F NMR** (CD_3CN, 300K, in ppm): δ = -132.3 (m, C6-F), -151.7 (m, C8-F), -162.8 (m, C7-F); **^{31}P{^{1}H} NMR** (CD_3CN, 300K, in ppm): δ = -78.6 (m, P); **elemental analysis**: calculated for $C_{17}H_{14}F_{10}NP$: C 43.1, H 1.1, N 6.3, found: C 43.0, H 0.9, N 6.0; crystallographic data and details of the structure refinements are included in Table 7.2.1.

3 Preparation of 3-4[OTf]

3.1 General procedure for the synthesis of Frustrated Lewis Pairs

Phosphane (1 mmol) and NFSI (315 mg, 1 mmol) were stirred in PhF (2 mL) at ambient temperature overnight. Subsequently, MeOTf (164 mg, 1 mmol) was added and the mixture was further stirred for 4 h. *n*-Pentane was added to the reaction mixture until a precipitate forms and the resulting suspension was filtered and washed with *n*-pentane (3 x 1 mL). The resulting solid was dried *in vacuo* to give the corresponding fluorophosphonium triflate as analytically pure, air sensitive, colorless solid.

3.2. Preparation of 3[OTf]

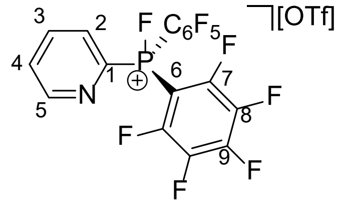

Following the general procedure in 3.1, **3**[OTf] was prepared from **13** (443 mg, 1.0 mmol).

Yield: 554 mg, 91%; **m.p.**: 113 – 114 °C; **3**[OTf] may change to purple after long-term storage. **Raman** (100 mW, 500 scans, 298 K, in cm^{-1}): 3083 (22), 1653 (100), 1579 (11), 1562 (85), 1407 (39), 1389 (24), 1312 (13), 1272 (24), 1225 (14), 1168 (11), 1133 (13), 1040 (13), 1032 (73), 988 (73), 955 (19), 893 (18), 870 (30), 755 (27), 638 (15), 615 (18), 588 (53), 574 (11), 530 (57), 473 (38), 441 (58), 415 (18), 391 (37), 350 (29), 314 (24), 281 (10), 227 (8), 203 (34); **IR** (ATR, 298 K, in cm^{-1}): 1651 (w), 1522 (m), 1491 (s), 1434 (vw), 1390 (w), 1311 (w), 1259 (s), 1225 (w), 1156 (m), 1109 (s), 1031 (s), 984 (vs), 954 (m), 869 (w), 767 (m), 731 (w), 637 (vs); **^{1}H NMR** (CD_2Cl_2, 300K, in ppm): δ = 8.95 – 8.94 (1H, m, C5-H), 8.77 – 8.74 (1H, m, C2-H), 8.25 – 8.20 (1H, m, C3-H), 7.93 – 7.90 (4H, m, C4-H); **^{13}C{^{1}H} NMR** (CD_2Cl_2, 300K, in ppm): δ = 152.4 (d, $^2J_{CP}$ = 29 Hz, C5), 149.3 (dm, $^1J_{CF}$ = 263 Hz, C9), 149.2 (dm, $^1J_{CF}$ = 263 Hz, C8), 142.3 (dd, $^1J_{CP}$ = 174 Hz, $^2J_{CF}$ = 14 Hz, C1), 139.3 (dm, $^1J_{CF}$ = 260 Hz , C7), 139.3 (d, $^3J_{CP}$ = 16 Hz, C3), 133.7 (d, $^2J_{CP}$ = 36 Hz, C2), 131.9 (d, $^4J_{CP}$ = 4 Hz, C4), 120.73 (q, $^1J_{CF}$ = 320 Hz, OTf), 96.5 (dm, $^1J_{CP}$ = 135 Hz, C6); **^{19}F NMR** (CD_2Cl_2, 300K, in ppm): δ = -79.4 (s, OTf), -114.0 (d, $^1J_{FP}$ = 996 Hz, P-F), -124.5 (m, C7-F), -133.2 (m, C9-F), -155.5 (m, C8-F): **^{31}P{^{1}H} NMR** (CD_2Cl_2, 300K, in ppm): δ = 54.8 (d, $^1J_{PF}$ = 996 Hz, P); **elemental analysis**: calculated for $C_{18}H_4F_{14}O_3NPS$: C 35.4, H 0.7, N 2.3, S 5.3, found: C 35.0, H 0.7, N 2.6, S 5.5; crystallographic data and details of the structure refinements are included in table 7.2.2.

3.3 Preparation of 4[OTf]

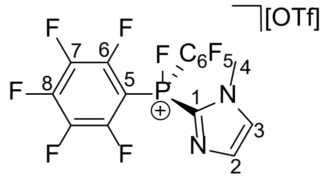

Following the general procedure in 3.1, **4**[OTf] was prepared from **14** (446 mg, 1.0 mmol).

Yield: 540 mg, 88%; **m.p.**: 83 – 84 °C; **Raman** (100 mW, 500 scans, 298 K, in cm^{-1}): 3098 (7), 2972 (13), 1653 (83), 1524 (7), 1501 (9), 1448 (29), 1406 (13), 1396 (33), 1349 (8), 1314 (100), 1286 (49), 1258 (5), 1227 (10), 1195 (40), 1153 (11), 1078 (48), 1032 (61), 947 (56), 91 (59), 898 (17), 867 (20), 758 (30), 683 (33), 592 (38), 574 (5), 565 (26), 547 (11), 492 (73), 443 (52), 419 (30), 391 (42), 364 (18), 350 (29), 333 (17), 315 (24), 252 (17), 220 (26); **IR** (ATR, 298 K, in cm^{-1}): 3097 (vw), 1652 (w), 1524 (m), 1497 (vs), 1407 (vw), 1395 (w), 1313 (s), 1274 (s), 1255 (vs), 1225 (m), 1171 (m), 1159 (m), 1113 (vs), 1078 (w), 1030 (s), 989 (vs), 939 (m), 917 (w), 866 (w), 831 (w), 769 (w), 758 (vw), 726 (w), 681 (w), 637 (vs), 591 (m), 565 (s), 545 (vs), 518 (m), 491 (w), 465 (s), 442 (vw); **^{1}H NMR** (CD_2Cl_2, 300K, in ppm): δ = 7.97 – 7.95 (1H, m, C2-H), 7.63 – 7.62 (1H, m, C3-H), 4.18 (3H, s, C4-H); **^{13}C{^{1}H} NMR** (CD_2Cl_2, 300K, in ppm): δ = 150.2 (dm, $^1J_{CF}$ = 276 Hz, C8), 149.4 (dm, $^1J_{CF}$ = 263 Hz, C7), 139.5 (dm, $^1J_{CF}$ = 260 Hz, C6), 137.9 (d, $^1J_{CP}$ = 26.7 Hz, C3), 136.7 (dd, $^3J_{CP}$ = 6.8 Hz, $^4J_{CF}$ = 2.6 Hz, C2), 123.9 (dd, $^1J_{CP}$ = 218 Hz, $^2J_{CF}$ = 25 Hz, C1), 120.0 (q, $^1J_{CP}$ = 320 Hz, OTf), 93.4 (dm, $^1J_{CP}$ = 141 Hz, C5), 36.9 (s, C4); **^{19}F NMR** (CD_2Cl_2, 300K, in ppm): δ = -79.4 (s, OTf), -111.5 (dm, $^1J_{FP}$ = 1045 Hz): δ = -126.3 (m, C7-F), -130.3 (m, C8-F), -154.5 (m, C6-F); **^{31}P{^{1}H}** NMR (CD_2Cl_2, 300K, in ppm): δ = 55.2 (d, $^1J_{FP}$ = 1045 Hz, P-F); **elemental analysis**: calculated for $C_{17}H_5F_{14}O_3N_2PS$: C 33.2, H 0.8, N 4.6, S 5.2, found: C 33.2, H 0.9, N 4.6, S 5.4; Crystallographic data and details of the structure refinements are included in Table 7.2.2.

4 Synthesis of compound 2[OTf]

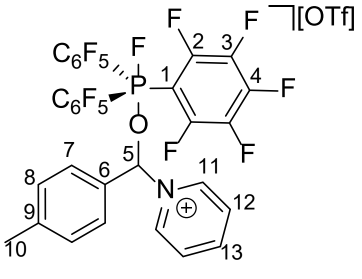

A solution of *p*-tolualdehyde (30.0 mg, 0.25 mmol) and pyridine (19.8 mg, 0.25 mmol) in CH_2Cl_2 (1 mL) was added dropwise to a suspension of **1**[OTf] (175 mg, 0.25 mmol) in CH_2Cl_2 (1 mL). The solution was then stirred at ambient temperature until a precipitate formed (ca. 4 h). The resulting suspension was filtrated, washed with CH_2Cl_2 (2 x 1 mL) and dried *in vacuo* to afford the adduct **2**[OTf] as a colorless solid.

Yield: 179 mg, 80%; **m.p.**: decomposition at approx. 143 °C; **Raman** (100 mW, 500 scans, 298 K, in cm^{-1}): 3100 (32), 2934 (14), 1647 (51), 1615 (32), 1583 (13), 1400 (8), 1384 (16),

1224 (10), 1201 (16), 1185 (25), 1032 (100), 865 (32), 830 (11), 815 (8), 791 (13), 757 (29), 646 (25), 638 (8), 585 (95), 493 (83), 474 (11), 445 (59), 387 (67), 348 (35), 313 (22), 286 (5), 273 (11), 232 (8), 149 (10), 135 (6); **IR** (ATR, 298 K, in cm^{-1}): 3128 (vw), 3058 (vw), 2967 (vw), 1646 (w), 1523 (m), 1482 (vs), 1398 (w), 1300 (w), 1263 (s), 1223 (w), 1210 (w), 1183 (w), 1152 (m), 1102 (vs), 1061 (m), 1031 (s), 981 (vs), 855 (vw), 811 (s), 790 (w), 769 (m), 763 (w), 743 (w), 728 (w), 717 (w), 682 (m), 657 (w), 646 (m), 635 (vs), 607 (m), 594 (m), 559 (s), 546 (s), 518 (m), 498 (w), 482 (w), 471 (m), 456 (w), 433 (m), 419 (w); **^{1}H NMR** (CD_3CN, 300K, in ppm): δ = 8.80 – 8.79 (2H, m, C12-H), 8.48 – 8.45 (1H, m, C13-H), 7.97 – 7.95 (2H, m, C11-H), 7.33 – 7.31 (2H, m, C7-H), 7.16 (1H, d, $^{3}J_{PH}$ = 13.0 Hz, C5-H), 7.14 (2H, m, C8-H), 2.26 (3H, s, C10-H); **^{13}C{^{1}H} NMR** (CD_3CN, 300K, in ppm): δ = 148.8 (s, C13), 146.3 (dm, $^{1}J_{CF}$ = 239 Hz, C4), 145.5 (dm, $^{1}J_{CF}$ = 255 Hz, C3), 142.4 (s, C9), 141.3 (s, C12), 134.2 (s, br, C6), 131.0 (s, C8), 130.3 (s, C11), 126.2 (s, C7), 122.2 (q, $^{1}J_{CF}$ = 199 Hz), 110.5 (dm, $^{1}J_{CP}$ = 199 Hz, C1), 94.2 (d, $^{2}J_{CP}$ = 11 Hz, C5), 21.3 (s, C10); **^{19}F NMR** (CD_3CN, 300K, in ppm): δ = 19.1 (d, $^{1}J_{FP}$ = 644 Hz, P-F), -79.4 (s, OTf), -132.3 (br, C2-F), -147.4 (br, C4-F), -159.9 (br, C3-F); **^{31}P{^{1}H} NMR** (CD_3CN, 300K, in ppm): δ = -53.2 (d, $^{1}J_{PF}$ = 644 Hz, P-F); **elemental analysis**: calculated for $C_{32}H_{13}F_{19}NO_4PS$: C 42.7, H 1.5, N 1.6, S 3.6, found: C 42.4, H 1.4, N 1.6, S 3.9; crystallographic data and details of the structure refinements are included in table 7.2.1.

5 Reaction of 3-4[OTf] with CO_2

5.1 Reaction of 3[OTf] with CO_2

A *J-Young* NMR tube was charged with a solution of **3**[OTf] (31 mg, 0.05 mmol) in CD_2Cl_2 (0.5 mL) in the glovebox. The system was then degassed and pressurized with CO_2 (0.5 – 2.0 bar). NMR spectra were measured at variable temperatures as shown in the manuscript.

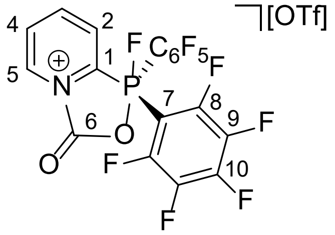

^{1}H NMR (CD_2Cl_2, 233K, in ppm): δ = 9.77 (1H, br, C5-H), 9.22 (1H, m, C3-H), 9.06 (1H, m, C2-H), 8.77 (1H, b, C4-H); **^{13}C{^{1}H} NMR** (CD_2Cl_2, 233K, in ppm): δ = 152.6 (d, $^{3}J_{CP}$ = 12 Hz, C3), 146.6 (dm, $^{1}J_{CF}$ = 263 Hz, C9), 145.3 (dm, $^{1}J_{CF}$ = 264 Hz, C10), 143.7 (d, $^{3}J_{CP}$ = 9 Hz, C5), 141.8 (d, $^{2}J_{CP}$ = 3 Hz, C6), 130.2 (d, $^{2}J_{CP}$ = 14 Hz, C2), 138.0 (dm, $^{1}J_{CF}$ = 273 Hz, C8), 135.4 (dd, $^{1}J_{CP}$ = 198 Hz, $^{2}J_{CF}$ = 44 Hz, C1), 134.5(s, C4), 119.9 (q, $^{1}J_{CF}$ = 320 Hz, OTf), 104.3 (dm, $^{1}J_{CP}$ = 185 Hz, C7); **^{19}F NMR** (CD_2Cl_2, 233K, in ppm): δ = -30.0 ($^{1}J_{FP}$ = 769 Hz), -79.8 (s, OTf), -130.5 (br, C8-F), -142.2 (br, C10-F), -157.6 (br, C9-F); **^{31}P{^{1}H} NMR** (CD_2Cl_2, 233K, in ppm): δ = -55.5 ($^{1}J_{PF}$ = 769 Hz), .

5.2 Reaction of 4[OTf] with CO_2

A *J-Young* NMR tube was charged with a solution of **4**[OTf] (30 mg, 0.05 mmol) in CD_3NO_2 (0.5 mL) in the glovebox. The system was then degassed and charged with CO_2 (0.5 - 2 bar). NMR spectra were measured with variable temperatures as shown in the manuscript.

Co-crystals containing both cations **6**$^+$ and **7**$^{2+}$ suitable for X-ray diffraction were obtained by vapor diffusion of CH_2Cl_2 into a solution of **4**[OTf] (20 mg) in CH_3NO_2 under CO_2 (0.5 bar) atmosphere and crystallographic details of the structure refinement are included in table 7.2.2.

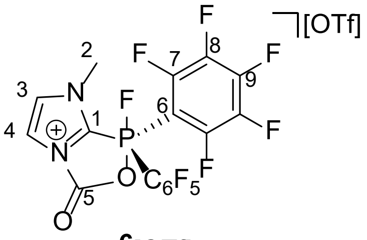

6[OTf]

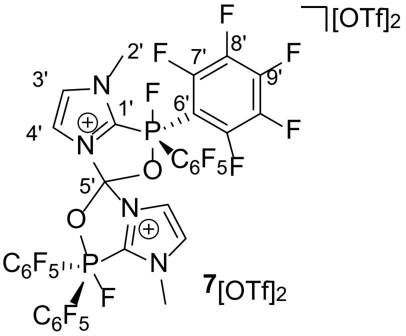

7[OTf]2

^{1}H NMR (CD_3NO_2, 243K, in ppm): δ = 8.54 (1H, s, C4-H), 8.35 (1H, s, C3-H), 8.20 (1H, s, C4'-H), 8.00 (1H, s, C3'-H), 4.44 (3H, s, C2-H), 4.40 (3H, s, C2'-H); **^{13}C{^{1}H} NMR** (CD_3NO_2, 243K, in ppm): δ = 146.7 (dm, $^1J_{CF}$ = 247 Hz, C8 and C8\`), 145.8 (dm, $^1J_{CF}$ = 261 Hz, C9 and C9\`), 140.2 (d, $^3J_{CP}$ = 10 Hz, C3'), 139.9 (d, $^2J_{CP}$ = 7 Hz, C5), 138.7 (d, $^3J_{CP}$ = 8 Hz, C3), 138.4 (dm, $^1J_{CF}$ = 254 Hz, C7 and C7\`), 132.6 (dd, $^1J_{CP}$ = 239 Hz, $^2J_{CF}$ = 50 Hz, C1), 131.6(dd, $^1J_{CP}$ = 239 Hz, $^2J_{CF}$ = 50 Hz, C1'), 122.6 (d, $^3J_{CP}$ = 9 Hz, C4), 122,1 (d, br, $^3J_{CP}$ = 9 Hz, C4'), 120.7 (q, $^1J_{CF}$ = 320 Hz, OTf), 106.8 (dm, $^1J_{CP}$ = 196 Hz, C6\`), 105.5 (t, $^2J_{CP}$ = 8 Hz, C5'), 103.7 (dm, $^1J_{CP}$ = 214 Hz, C6), 39.7 (d, $^3J_{CP}$ = 11 Hz, C2'), 39.2 (d, $^3J_{CP}$ = 11 Hz, C2); **^{19}F NMR** (CD_3NO_2, 243K, in ppm): δ = -8.1 (d, $^1J_{FP}$ = 737 Hz, P(**7**$^{2+}$)-F), -16.9 (d, $^1J_{FP}$ = 740 Hz, P(**6**$^+$)-F), -80.1 (s, OTf), -130.2 (br, C7\`-F), -132.5 (br, C7-F), -133.1 (br, C7\`-F), -143.2 (br, C9\`-F), -144.0 (br, C9-F), -144.5 (br, C9\`-F), -159.0 (br, C8\`-F), -159.5 (br, C8-F), -159.7 (br, C8\`-F), the ^{19}F NMR spectrum reveals five signal sets for the C_6F_5-groups in **7**$^{2+}$ which we attribute to a hindered rotation around the C6\`-P bonds; **^{31}P{^{1}H} NMR** (CD_3NO_2, 243K, in ppm): δ = -60.0 (d, $^1J_{PF}$ = 737 Hz, P(**7**$^{2+}$), integral ratio), -61.5 (d, $^1J_{PF}$ = 740 Hz, P(**6**$^+$), 28% integral ratio).

6 Reaction of 3-4[OTf] with dipolarophiles

6.1 General reaction conditions for the reaction of 3-4[OTf] with carbonyl and nitriles

In the glovebox, a solution of **3-4**[OTf] and substrate (1 eq.) (carbonyle or nitrile) was stirred in CH_2Cl_2 (2 mL) for a definite time. Upon addition of *n*-pentane a precipitate forms, which was filtered off, washed with *n*-pentane (3 x 1 mL) and dried *in vacuo* yielding the titled compound as solid material.

6.2 Preparation of 8a[OTf]

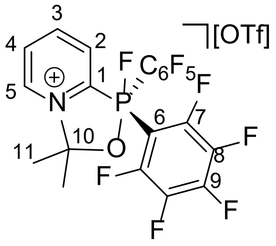

According to the general procedure in 6.1, **8a**[OTf] was prepared from the reaction of **3**[OTf] (611 mg, 1 mmol) with acetone (58 mg, 1 mmol) for 1 h.

Yield: 646 mg, 97%, colorless solid; **m.p.**: 143 – 144 °C; **Raman** (100 mW, 500 scans, 298 K, in cm^{-1}): 3087 (19), 3018 (12), 2987 (16), 2954 (23), 1648 (37), 1605 (33), 1576 (23), 1463 (9), 1399 (12), 1324 (12), 1300 (9), 1225 (16), 1168 (14), 1114 (28), 1048 (51), 1031 (100), 856 (19), 755 (33), 706 (33), 666 (19), 590 (65), 574 (19), 542 (21), 512 (26), 488 (74), 445 (42), 388 (51), 349 (42), 327 (16), 311 (35), 284 (19), 268 (19), 178 (53); **IR** (ATR, 298 K, in cm^{-1}): 3103 (vw), 3082 (vw), 1648 (vw), 1605 (vw), 1523 (m), 1486 (s), 1443 (vw), 1412 (vw), 1397 (vw), 1377 (vw), 1301 (w), 1262 (vs), 1224 (w), 1178 (w), 1155 (m), 1132 (m), 1104 (vs), 1049 (vw), 1029 (s), 1006 (m), 981 (vs), 876 (m), 804 (w), 766 (m), 756 (s), 737 (m), 724 (m), 705 (w), 665 (w), 637 (vs); **^{1}H NMR** (CD_2Cl_2, 300K, in ppm): δ = 10.12 (1H, m, C5-H), 8.97 – 8.91 (2H, m, C2-H, C3-H), 8.75 – 8.73 (1H, m, C4-H), 1.87 (6H, s, C11-H); **^{13}C{^{1}H} NMR** (CD_2Cl_2, 300K, in ppm): δ = 148.2 (d, $^3J_{CP}$ = 10 Hz, C3), 146.3 (dm , $^1J_{CF}$ = 254 Hz, C8), 145.1 (dm, $^1J_{CF}$ = 258 Hz, C9), 145.0 (d, $^3J_{CP}$ = 11 Hz, C5), 138.6 (dm, $^1J_{CF}$ = 257 Hz, C7), 137.8 (dd, $^2J_{CP}$ = 15 Hz, $^3J_{CF}$ = 9 Hz, C2), 136.4 (dd, $^1J_{CP}$ = 193 Hz, $^2J_{CF}$ = 53 Hz, C1), 135.2 (d, $^4J_{CP}$ = 2 Hz, C4), 121.7 (q, $^1J_{CF}$ = 320 Hz, OTf), 110.7 (dm, $^1J_{CP}$ = 198 Hz, C6), 103.6 (d, $^2J_{CP}$ = 10 Hz, C10), 29.3 (s, C11); **^{19}F NMR** (CD_2Cl_2, 300K, in ppm): δ = -6.5 (dm, $^1J_{PF}$ = 734 Hz, P-F), -79.1 (s, OTf), -131.8 (m, C7-F), -144.7 (m, C9-F), -158.1 (m, C8-F); **^{31}P{^{1}H} NMR** (CD_2Cl_2, 300K, in ppm): δ = -52.0 (d, $^1J_{PF}$ = 734 Hz, P-F); **elemental analysis**: calculated for $C_{21}H_{10}F_{14}NO_4PS$: C 37.7, H 1.5, N 2.1, S 4.8, found C 38.0, H 1.8, N 1.9, S 4.8; crystallographic data and details of the structure refinements are included in table 7.2.3.

6.3 Preparation of 8b[OTf]

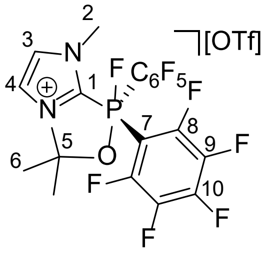

According to the general procedure in 6.1, **8b**[OTf] was prepared from the reaction of **4**[OTf] (92 mg, 0.15 mmol) and acetone (9 mg, 0.15 mmol) for 1 h.

Yield: 84 mg, 84%, colorless solid; **m.p.**: decomposed at approx. 168 °C; **Raman** (100 mW, 500 scans, 298 K, in cm^{-1}): 3009 (9), 2979 (9), 2955 (22), 1649 (43), 1465 (13), 1398 (35), 1372 (100), 1319 (20), 1227 (17), 1099 (9), 1034 (85), 854 (20), 756 (33), 713 (11), 602 (26), 583 (22), 574 (20), 558 (13), 544 (63), 491 (52), 445 (37), 418 (15), 388 (33), 350 (43), 331 (22), 314 (37); **IR** (ATR, 298 K, in cm^{-1}): 3124 (vw), 1648 (w), 1521 (m), 1487 (vs), 1398 (w), 1371 (w), 1300 (w), 1262 (vs), 1227 (m), 1151 (s), 1104 (vs), 1032 (vs), 987 (vs), 877 (m), 796 (vw), 768 (s), 753 (s), 729 (w), 670 (w), 637 (vs), 595 (s), 572 (m), 557 (vs), 543 (s), 518 (s), 490 (w), 477 (w), 462 (m), 444 (vw), 422 (w), 372 (vw); **^{1}H NMR** (CD_2Cl_2, 300K, in ppm): δ = 8.46 (1H, m, C4-H), 8.26(1H, m,C3-H), 4.23 (3H, d, $^{4}J_{HP}$ = 3.7 Hz, C2-H), 1.73(6H, s, C6-H); **^{13}C{^{1}H} NMR** (CD_2Cl_2, 300K, in ppm): δ = 146.1 (dm, $^{1}J_{CF}$ = 254 Hz, C9), 145.0 (dm, $^{1}J_{CF}$ = 264 Hz, C10), 138.5 (dm, $^{1}J_{CF}$ = 240 Hz, C8), 138.2 (d, $^{3}J_{CP}$ = 6 Hz, C3), 130.8 (dd, $^{1}J_{CP}$ = 234 Hz, $^{2}J_{CF}$ = 60 Hz, C1), 122.7 (d, $^{3}J_{CP}$ = 11 Hz, C4), 121.2 (q, $^{1}J_{CF}$ = 320 Hz, OTf), 110.4(dm, $^{1}J_{CP}$ = 201 Hz, C7), 96.7 (d, $^{2}J_{CP}$ = 11 Hz, C5), 35.6 (d, $^{3}J_{CP}$ = 12 Hz, C2), 28.1 (s, C6); **^{19}F NMR** (CD_2Cl_2, 300K, in ppm): δ = 9.6 (dm, $^{1}J_{FP}$ = 696 Hz, P-F), -79.1 (s, OTf), -132.1 (m, C8-F), -144.5 (m, C10-F), -158.1 (m, C9-F); **^{31}P{^{1}H} NMR** (CD_2Cl_2, 300K, in ppm): δ = -61.0 (d, $^{1}J_{PF}$ = 696 Hz, P-F). **elemental analysis**: calculated for $C_{20}H_{11}F_{14}N_2O_4PS$: C 35.7, H 1.7, N 4.2, S 4.8, found: C 35.4, H 1.6, N 4.1, S 5.0; crystallographic data and details of the structure refinements are included in table 7.2.3.

6.4 Preparation of 9a[OTf]

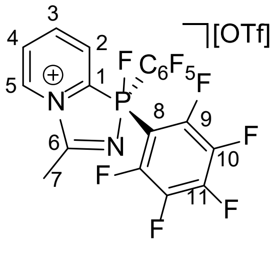

According to the general procedure in 6.1, **9a**[OTf] was prepared from the reaction of **3**[OTf] (611 mg, 1.0 mmol) and MeCN (44 mg, 1.1 mmol) in CH_2Cl_2 (2 mL) for 1 h.

Yield: 463 mg, 71%, colorless solid; **m.p.**: decomposition at approx. 128 °C**;** **Raman** (100 mW, 500 scans, 298 K, in cm^{-1}): 3118 (8), 3076 (19), 3043 (7), 2944 (27), 1731 (62), 1648 (44), 1609 (20), 1580 (12), 1407 (26), 1376 (13), 1340 (16), 1298 (14), 1145 (20), 1096 (56), 1044 (56), 1032 (100), 853 (14), 758 (33), 704 (24), 672 (15), 587 (55), 550 (13), 517 (18), 505 (14), 487 (73), 445 (41), 406 (12), 388 (41), 350 (31), 315 (18), 282 (14), 210 (33), 175 (56); **IR** (ATR, 298 K, in cm^{-1}): 3127 (vw), 3098 (vw), 3029 (vw), 1729 (w), 1649 (vw), 1608 (vw), 1520 (w), 1488 (vs), 1404 (w), 1377 (vw),

1295 (w), 1264 (m), 1248 (s), 1226 (w), 1158 (m), 1145 (m), 1101 (vs), 1095 (vs), 1030 (s), 980 (vs), 908 (vw), 878 (vw), 781 (w), 768 (s), 755 (s), 737 (w), 727 (w), 704 (w), 669 (m), 637 (vs), 614 (vs); **^{1}H NMR** (CD_3CN, 300K, in ppm): δ = 9.56 – 9.55 (1H, m, C5-H), 9.12 – 9.09 (1H, m, C2-H), 9.04 – 9.00 (1H, m, C3-H), 8.62 – 8.59 (1H, m, C4-H), 1.96 (3H, s, C7-H); **^{13}C{^{1}H} NMR** (CD_3CN, 300K, in ppm): δ = 151.0 (d, $^2J_{CP}$ = 18 Hz, C6), 150.6 (d, $^3J_{CP}$ = 9 Hz, C3), 147.2 (dm, $^1J_{CF}$ = 252 Hz, C9), 145.5 (dm, $^1J_{CF}$ = 255 Hz, C11), 144.1 (d, $^3J_{CP}$ = 13 Hz, C5), 140.0 (d, $^1J_{CP}$ = 224 Hz, C1), 139.3 (dm, $^1J_{CF}$ = 254 Hz, C10), 139.7 (dd, $^2J_{CP}$ = 14 Hz, $^3J_{CF}$ = 6 Hz, C2), 134.4 (dd, $^4J_{CP}$ = 2 Hz, $^5J_{CF}$ = 1 Hz, C4), 122.4 (q, $^1J_{CF}$ = 346 Hz, OTf), 106.5 (dm, $^1J_{CP}$ = 188 Hz, C8), 1.8 (s, C7); **^{19}F NMR** (CD_3CN, 300K, in ppm): δ = 3.0 (dm, $^1J_{FP}$ = 738 Hz, P-F), -79.4 (s, OTf), -131.2 (m, C9), -147.5 (m, C11), -160.5 (m, C10); **^{31}P{^{1}H} NMR** (CD_3CN, 300K, in ppm): δ = -49.3 (d, $^1J_{PF}$ = 738 Hz, P-F); **Elemental analysis**: calculated for $C_{20}H_7F_{14}N_2O_3PS$: C 36.8, H 1.1, N 4.3, S 4.9, found: C 36.6, H 0.9, N 4.2, S 5.2; crystallographic data and details of the structure refinements are included in Table 7.2.3.

6.5 Preparation of 9b[OTf]

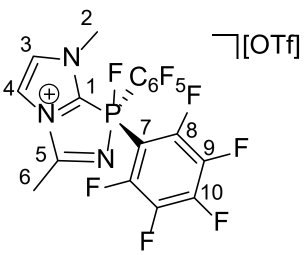

According to the general procedure in 6.1, **9b**[OTf] was prepared from the reaction of **4**[OTf] (615 mg, 1.0 mmol) and MeCN (40 mg, 1.0 mmol) in CH_2Cl_2 (2 mL) for 1 h.

Yield: 620 mg, 95%, colorless solid; **m.p.**: decomposition at approx. 153 °C; **Raman** (100 mW, 500 scans, 298 K, in cm^{-1}): 3182 (5), 3112 (6), 3033 (7), 2980 (22), 2933 (26), 1697 (29), 1650 (56), 1500 (9), 1428 (17), 1404 (56), 1387 (95), 1369 (100), 1307 (38), 1279 (10), 1266 (13), 1225 (17), 1098 (9), 1035 (91), 990 (13), 851 (16), 756 (40), 707 (14), 592 (47), 577 (28), 563 (13), 533 (43), 491 (82), 468 (14), 445 (52), 436 (26), 419 (23), 388 (46), 350 (25), 338 (13), 314 (28), 283 (17), 264 (13), 231 (18), 170 (59); IR (ATR, 298 K, in cm^{-1}): 3111 (vw), 1696 (w), 1650 (w), 1522 (m), 1497 (s), 1488 (vs), 1430 (vw), 1402 (w), 1385 (w), 1368 (w), 1296 (w), 1266 (vs), 1225 (m), 1200 (w), 1158 (s), 1102 (vs), 1032 (s), 979 (vs), 768 (s), 755 (s), 726 (w), 706 (vw), 674 (m), 637 (vs), 608 (vs); **^{1}H NMR** (CD_3NO_2, 300K, in ppm): δ = 8.25 (1H, s, C4-H), 8.13 (1H, s, C3-H), 4.33 (3H, together with CH_3NO_2 peak, C2-H), 2.64 (3H,s, C6-H); **^{13}C{^{1}H} NMR** (CD_3NO_2, 300K, in ppm): δ = 150.2 (d, $^2J_{CP}$ = 19 Hz, C5), 147.7 (dm, $^1J_{CF}$ = 252 Hz, C9), 145.9 (d, $^1J_{CF}$ = 259 Hz, C10), 139.7 (dm, $^1J_{CF}$ = 257 Hz, C8), 139.2 (dd, $^1J_{CP}$ = 187 Hz, $^2J_{CF}$ = 67 Hz, C1), 138.9 (dd, $^3J_{CP}$ = 4 Hz, $^4J_{CF}$ = 2 Hz, C3), 122.2 (q, $^1J_{CF}$ = 351 Hz, OTf), 121.7 (d, $^3J_{CP}$ = 13 Hz, C4), 106.4 (dm, $^1J_{CP}$ = 190 Hz, C7), 40.1 (d, 10 Hz, C2), 16.6 (dd, $^3J_{CP}$ = 11 Hz, $^4J_{CF}$ = 2 Hz, C6); **^{19}F NMR** (CD_3NO_2, 300K, in ppm): δ = 19.9 (dm, $^1J_{PF}$ = 702 Hz, P-F), -79.8 (s, OTf), -132.1 (br, C8-F), -147.8 (m, C10-F), -160.7 (m, C9-F);

$^{31}P\{^1H\}$ NMR (CD_3NO_2, 300K, in ppm): δ = -65.0 (d, $^1J_{PF}$ = 702 Hz, P-F); **Elemental analysis**: calculated for $C_{19}H_{18}F_{14}N_3O_3PS$: C 34.8, H 1.2, N 6.4, S 4.9, found: C 34.4, H 1.0, N 6.5, S 4.9; crystallographic data and details of the structure refinements are included in table 7.2.4.

6.6 Preparation of 11a[OTf]$_2$

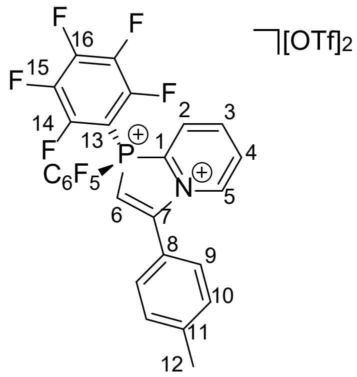

According to the general procedure in 6.1, **11a**[OTf]$_2$ was prepared from the reaction of **3**[OTf]) (306 mg, 0.5 mmol) and 4-ethynyltoluene (29 mg, 0.25 mmol) in DCE (2 mL) at r.t. for 4 h. The resulting dark green suspension was filtrated, washed with DCE (3 x 1 mL) and dried *in vacuo* to give the titled compound.

Yield: 168 mg, 78%, dark green solid; **m.p.**: decomposition at approx. 177 °C; **Raman:** decomposed under laser irradiation; **IR** (ATR, 298 K, in cm^{-1}): 3070 (vw), 1650 (w), 1628 (vw), 1522 (m), 1495 (vs), 1436 (vw), 1408 (vw), 1397 (vw), 1312 (w), 1258 (vs), 1226 (m), 1200 (vw), 1161 (m), 1109 (vs), 1079 (vw), 1031 (vs), 986 (s), 938 (vw), 878 (vw), 841 (vw), 817 (w), 790 (w), 760 (vw), 737 (vw), 725 (w), 637 (vs), 591 (w), 568 (w), 543 (m), 529 (w), 518 (m), 500 (vw), 478 (w), 448 (vw), 435 (m), 417 (vw); **^1H NMR** (CD_3CN, 300K, in ppm): δ = 9.36 – 9.34 (1H, ddd, C5-H), 9.18 – 9.14 (1H, t, C2-H), 9.06 – 9.00 (1H, ddt, C4-H), 8.57 – 8.53 (1H, qt, C3-H), 7.78 (1H, d, $^2J_{PH}$ = 22.0 Hz, C6-H), 7.75 – 7.73 (2H, m, C10-H), 7.62 – 7. 60 (2H, m, C9-H), 2.54 (3H, s, C12-H); **$^{13}C\{^1H\}$ NMR** (CD_3CN, 300K, in ppm): δ = 166.5 (s, C7), 152.1 (d, $^4J_{CP}$ = 8 Hz, C4), 150.3 (dm, $^1J_{CF}$ = 259 Hz, C15), 149.7 (dm, $^1J_{CF}$ = 261 Hz, C16), 148.0 (d, $^3J_{CP}$ = 6 Hz, C5), 147.0 (s, C11), 140.2 (dm, $^1J_{CF}$ = 256 Hz, C14), 136.8 (m, C2), 135.3 (s, C3), 135.1 (d, $^1J_{CP}$ = 117 Hz, C1), 131.7 (s, C9), 131.5 (s, C10), 123.0 (q, $^1J_{CF}$ = 342 Hz, OTf), 122.4 (d, $^3J_{CP}$ = 15 Hz, C8), 101.0 (d, $^1J_{CP}$ = 89 Hz, C6), 91.2 (dm, $^1J_{CP}$ = 102 Hz, C13), 21.5 (s, C12); **^{19}F NMR** (CD_3CN, 300K, in ppm): δ = -79.4 (s, OTf), -124.1 (m, C14-F), -135.7 (m, C16-F), -157.1 (m, C15-F); **$^{31}P\{^1H\}$ NMR** (CD_3CN, 300K, in ppm): δ = 6.9 (s, P); **elemental analysis**: calculated for $C_{28}H_{12}F_{16}NO_6PS_2$: C 39.2, H 1.4, N 1.6, S 7.5, found: C 38.9, H 1.2, N 1.8, S 7.3.

6.7 Preparation of 12b[OTf]

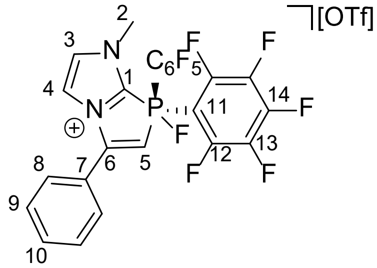

In a 10 mL Schlenk tube, the mixture of **4**[OTf] (615 mg, 1.0 mmol) and phenylacetylene (116 mg, 1.0 mmol) in DCE (2 mL) was allowed to reacted at 80 °C for 16 h. After cooling down to *r.t.*, Et_2O (5 mL) was added to the resulting yellow solution. The suspension was allowed to settle, and the upper layer was decanted off. The residual oil was washed with Et_2O

until it solidifies. The suspension was filtrated, washed with Et_2O (3 x 1 mL) and dried *in vacuo* to give the titled product as light yellow solid.

Yield: 485 mg, 66%; **m.p.**: decomposition at approx. 106 °C; **Raman** (100 mW, 500 scans, 298 K, in cm^{-1}): 3075 (13), 2975 (7), 2221 (11), 1629 (100), 1599 (75), 1577 (10), 1560 (12), 1498 (6), 1473 (7), 1450 (5), 1401 (20), 1374 (18), 1332 (5), 1263 (22), 1213 (14), 1195 (7), 1163 (7), 1032 (33), 1001 (54), 860 (7), 806 (12), 756 (13), 706 (5), 653 (6), 626 (8), 616 (10), 587 (16), 573 (7), 531 (9), 490 (20), 446 (15), 415 (10), 401 (20), 386 (20), 349 (26), 314 (24), 282 (22), 251 (32); **IR** (ATR, 298 K, in cm^{-1}): 3127 (vw), 1648 (vw), 1627 (vw), 1523 (m), 1487 (vs), 1450 (vw), 1399 (w), 1331 (vw), 1265 (s), 1224 (w), 1194 (vw), 1159 (m), 1150 (m), 1104 (s), 1052 (vw), 1031 (s), 982 (s), 936 (vw), 922 (vw), 860 (vw), 801 (w), 762 (m), 727 (vw), 697 (w), 678 (m), 651 (m), 637 (vs); **^{1}H NMR** (CD_2Cl_2, 300K, in ppm): δ = 7.80 – 7.78 (2H, m, C8-H), 7.77 – 7.73 (1H, m, C10-H), 7.69 – 7.66 (2H, m, C9-H), 7.51 (1H, m, C4-H), 7.49 (1H, m, C3-H), 6.06 (1H, d, $^2J_{PH}$ = 26.3 Hz, C5-H), 3.60 (s, 3H, C2-H); **^{13}C{^{1}H} NMR** (CD_2Cl_2, 300K, in ppm): δ = 159.1 (dd, $^1J_{CP}$ = 66 Hz, $^2J_{CF}$ = 24 Hz, C1), 156.6 (d, $^3J_{CP}$ = 13 Hz, C7), 147.0 (dm, $^1J_{CF}$ = 252 Hz, C13), 145.2 (dm, $^1J_{CF}$ = 266 Hz, C14), 139.0 (dm, $^1J_{CF}$ = 256 Hz, C12), 134.2 (s, C10), 130.8 (s, C3), 130.5 (s, C9), 129.2 (s, C8), 127.2 (d, $^2J_{CP}$ = 18 Hz, C6), 121.2 (q, $^1J_{CF}$ = 326 Hz, OTf), 117.63 (s, C4), 106.8 (dm , $^1J_{CP}$ = 182 Hz, C11), 104.0 (dd, $^1J_{CP}$ = 167 Hz, $^2J_{CF}$ = 52 Hz, C5), 38.3 (m, C2); **^{19}F NMR** (CD_2Cl_2, 300K, in ppm): δ = 25.2 (dm, $^1J_{FP}$ = 718 Hz, P-F), -79.2 (s, OTf), -131.0 (br, C13-F), -144.3 (m, C14-F), -157.1 (m, C12-F); **^{31}P{^{1}H} NMR** (CD_2Cl_2, 300K, in ppm): δ = -98.8 (dm, $^1J_{PF}$ = 718 Hz, P-F); **elemental analysis**: calculated for $C_{31}H_{14}F_{14}NO_4PS$: N 4.0, C 41.9, H 1.6, S 4.5. found: N 4.0, C 41.7, H 1.7, S 4.5. crystallographic data and details of the structure refinements are included in Table 7.2.4.

6.8 Aceton-acetonitrile exchange reaction

In the glovebox, a solution of acetone (6 mg, 0.1 mmol) in CH_2Cl_2 (0.5 mL) was added to the suspension of **9a/b**[OTf] (0.1 mmol) in CH_2Cl_2 (0.5 mL). The mixture was stirred at room temperature till the suspension changed to a colorless solution (ca. 1 h) and measured NMR spectra at 300 K with C_6D_6-Capillary.

7 Crystallographic details

7.1 X-ray Diffraction refinements

Single crystals suitable for X-ray analysis are obtained by vapor diffusion of poor solvent into a saturated solution of the target compound, coated with Paratone-N oil or Fomblin Y25 PFPE oil, mounted using a nylon loop and frozen in the cold nitrogen stream. Crystals are measured at low temperatures on a Rigaku Oxford Diffraction SuperNova diffractometer using Cu Kα radiation (λ = 1.54184 Å) generated by a Nova micro-focus source. Crystal and data collection details are given in Table 7.2.1 – 7.2.4. Data reduction and absorption correction is performed with CrysaAlisPro software. Using Olex2[4], the structures are solved with SHELXS/T[5] by direct methods and refined with SHELXL[6] by least-square minimization against F^2 using first isotropic and later anisotropic thermal parameters for all non-hydrogen atoms. Hydrogen atoms bonded to carbon atoms are added to the structure models on calculated positions using the riding model. All other hydrogen atoms are localized in the difference Fourier map. If necessary, disorders of solvent molecules, anions or both are treated with appropriate restraints (SIMU, DFIX, RIGU, SAME). Due to heavily disordered solvent molecules in **6**[OTf]·**7**[OTf]$_2$·CH_3NO_2, the SQUEEZE[7] algorithm was applied. The volume of 583 A^3 and and the electron count of 224 accounts for 5 molecules of dichloromethane per void or 10 molecules per unit cell. **6**[OTf]·**7**[OTf]$_2$·CH_3NO_2 was refined as a non-merohedral twin related by a 180° rotation around [010] with two components in the ratio 54:46. **12b**[OTf] was refined as an inversion twin in the ratio 60:40. Images of the structures are produced with Olex2 software. All structures have been deposited with the Cambridge Crystallographic Data Centre (CCDC) and can be accessed free of charge under the numbers 2061970-2061980.

Molecular structures of compounds with Selected bond lengths and angles:

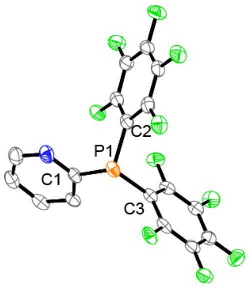

Figure S**1.** Molecular structure of **13** (hydrogen atoms are omitted for clarity, ellipsoids are set at 50% probability); Selected bond lengths (Å) and angles (˚): P1-C1 1.833(15), P1-C2 1.8445(15), P1-C3 1.8389(16); C1-P1-C2 101.61(7), C2-P1-C3 102.15(6), C1-P1-C3 103.75(7).

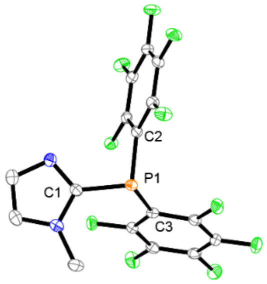

Figure S**2.** Molecular structure of **14** (hydrogen atoms are omitted for clarity, ellipsoids are set at 50% probability); Selected bond lengths (Å) and angles (˚): P1-C1 1.8168(13), P1-C2 1.8347(13), P1-C3 1.8504(14); C1-P1-C2 98.75(6), C2-P1-C3 100.36(6), C1-P1-C3 104.84(6).

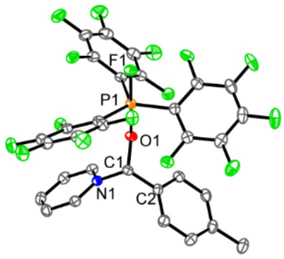

Figure **S3**. Molecular structure of **2**[OTf]·CH_2Cl_2 (hydrogen atoms, CH_2Cl_2, and non-coordinating anions are omitted for clarity, ellipsoids are set at 50% probability); Selected bond lengths (Å) and angles (˚): F1-P1 1.6643(9), P1-O1 1.7038(10), O1-C1 1.3974(16), C1-N1 1.5206(17), C1-C2 1.5124(19), F1-P1-O1 175.88 (5), P1-O1-C1 130.64(9), O1-C1-C2 112.21(11), O1-C1-N1 105.62(11), C2-C1-N1 109.65(11).

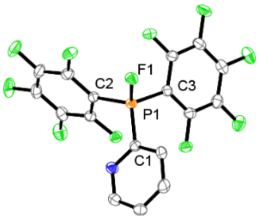

Figure S**4.** Molecular structure of **3**[OTf]·C_6H_5F (hydrogen atoms, C_6H_5F and non-coordinating anions are omitted for clarity, ellipsoids are set at 50% probability); Selected bond lengths (Å) and angles (˚): F1-P1 1.5282(11), C1-P1 1.7866(18), C2-P1 1.7726(17), C3-P1 1.7761(18), C1-P1-C2 113.35(8), C2-P1-C3 108.72(8), C3-P1-C1 113.35(8).

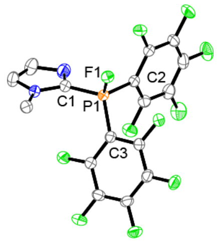

Figure S**5.** Molecular structure of **4**[OTf] (hydrogen atoms and non-coordinating anions are omitted for clarity, ellipsoids are set at 50% probability); Selected bond lengths (Å) and angles (˚): F1-P1 1.5338(11), C1-P1 1.7356(19), C2-P1 1.7722(19), C3-P1 1.7713(18), C1-P1-C2 108.70(9), C2-P1-C3 110.01(8), C3-P1-C1 116.85(9).

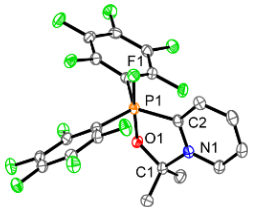

Figure **S6.** Molecular structure of **8a**[OTf]·CH_2Cl_2 (hydrogen atoms, CH_2Cl_2 and non-coordinating anions are omitted for clarity, ellipsoids are set at 50% probability); Selected bond lengths (Å) and angles (˚): F1-P1 1.673(13), P1-O1 1.6989(15), N1-C1 1.519(3), P1-C2 1.8204(19), O1-C1 1.398(2), P1-O1-C1 119.57(13), O1-C1-N1 103.54(15), C1-N1-C2 114.71(15), N1-C2-P1 111.88(14), C2-P1-O1 87.07(8), F1-P1-O1 176.71(7).

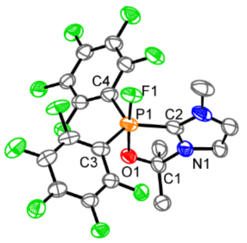

Figure S7. Molecular structure of **8b**[OTf]·CH_2Cl_2 (hydrogen atoms, CH_2Cl_2 and non-coordinating anions are omitted for clarity, ellipsoids are set at 50% probability); Selected bond lengths (Å) and angles (˚): F1-P1 1.637(2), P1-O1 1.695(2), O1-C1 1.411(5), N1-C1 1.495(4), P1-C3 1.818(4), P1-C2 1.811(4), P1-C4 1.817(4), P1-O1-C1 120.5(19), O1-C1-N1 102.0(3), C1-N1-C2 115.9(3), N1-C2-P1 112.9(3), C2-P1-O1 85.93(15), C2-P1-C3 126.16(17), C3-P1-C4 117.02(18), C4-P1-C2 116.75(18), F1-P1-O1 175.49(12).

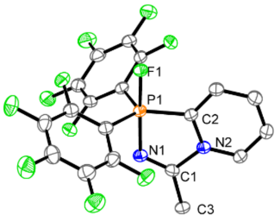

Figure S8. Molecular structure of **9a**[OTf]·$(CH_2Cl_2)_2$·CH_3CN (hydrogen atoms, CH_2Cl_2, CH_3CN and non-coordinating anions are omitted for clarity, ellipsoids are set at 50% probability); Selected bond lengths (Å) and angles (˚): F1-P1 1.6427(16), P1-N1 1.777(2), N1-C1 1.250(3), C1-N2 1.491(3), N2-C2 1.353(3), C2-P1 1.832(2), F1-P1-N1 177.56(10), C2-P1-N1 88.69(10), P1-N1-C1 114.50(18), N1-C1-N2 114.8(2), C1-N2-C1 111.96(19), N2-C2-P1 110.08(16).

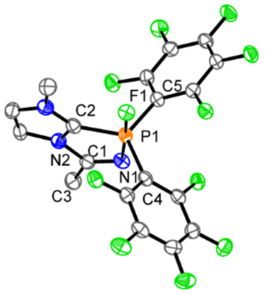

Figure S9. Molecular structure of **9b**[OTf] (hydrogen atoms and non-coordinating anions are omitted for clarity, ellipsoids are set at 50% probability); Selected bond lengths (Å) and angles (˚): F1-P1 1.6426(16), P1-N1 1.788(2), P1-C2 1.816(3), N1-C1 1.272(3), C1-N2 1.458(4), P1-C4 1.820(2), P1-C5 1.812(2), F1-P1-N1 176.63(9), N1-P1-F2 88.06(11), P1-N1-C1 113.83(19), N1-C1-N2 114.4(2), C1-N2-C2 112.7(2), N2-C2-P1 110.86(18), C2-P1-C4 122.68(10), C2-P1-C5 117.05(11), C4-P1-C5 120.27(11).

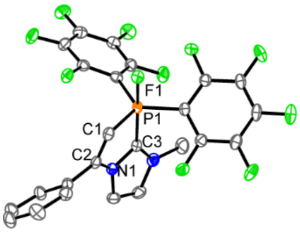

Figure S**10.** Molecular structure of **12b**[OTf] (hydrogen atoms and non-coordinating anions are omitted for clarity, ellipsoids are set at 50% probability); Selected bond lengths (Å) and angles (˚): F1-P1 1.651(3), P1-C3 1.970(5), P1-C1 1.773(5), C1-C2 1.330(8), C2-N1 1.415(6), F1-P1-C3 175.61(19), C1-P1-C3 84.2(2), P1-C3-N1 108.9(3), C3-N1-C2 115.5(5), N1-C2-C1 113.0(5), C2-C1-P1 117.6(4).

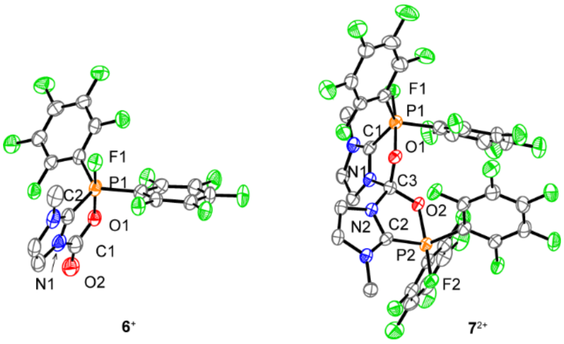

Figure **S11**. Molecular structure of **6**[OTf]·**7**[OTf]$_2$·CH_3NO_2 (hydrogen atoms, CH_3NO_2, and non-coordinating anions are omitted for clarity, ellipsoids are set at 50% probability). Selected bond lengths (Å) and angles (˚); for **6**$^+$: F1-P1 1.623(5), P1-C2 1.803(5), P1-O1 1.773(5), O1-C1 1.307(8), C1-N1 1.442(10), C1-O2 1.206(10), F1-P1-O1 176.3(2), C2-P1-O1 94.3(3), P1-O1-C1 118.2(5), O1-C1-O2 128.1(7), O1-C1-N1 109.2(6), O2-C1-N1 122.7(6), C1-N1-C2 144.5(5), N1-C2-P1 122.1(5); for **7**$^{2+}$: P1-F1 1.609(3), P2-F2 1.618(3), P1-O1 1.771(4), P2-O2 1.744(4), P1-C1 1.804(6), P2-C2 1.805(5), O1-C3 1.352(7), N1-C3 1.452(8), O2-C3 1.366(6), N2-C3 1.486(6), F1-P1-O1 176.1(2), F2-P2-O2 173.65(19), O1-C3-O2 114.4(5), N1-C3-N2 109.8(4), N1-C3-O1 105.6(4), N2-C3-O2 103.3(4), P1-O1-C3 120.5(4), P2-O2-C3 117.5(3).

7.2 Crystallographic details.

7.2.1. Crystallographic data and details of the structure refinements of compounds Py(C_6F_5)$_2$P **13**, MeIm(C_6F_5)$_2$P **14** and **2**[OTf].

	**13**	**14**	**2**[OTf]
Empirical formula	$C_{17}H_4F_{10}NP$	$C_{16}H_5F_{10}N_2P$	$C_{34}H_{17}Cl_4F_{19}NO_4PS$
Formula weight	443.18	446.19	1069.32
Temperature/K	100.01(10)	100.01(10)	100.0(5)
Crystal system	monoclinic	monoclinic	monoclinic
Space group	$P2_1/c$	$P2_1/c$	$P2_1/n$
a/Å	10.18410(15)	7.56361(7)	18.03115(11)
b/Å	15.2605(2)	18.20379(16)	12.61146(6)
c/Å	11.33089(16)	11.74482(10)	19.53512(12)
α/°	90	90	90
β/°	113.1366(17)	97.5328(8)	116.5999(8)
γ/°	90	90	90
Volume/Å^3	1619.34(5)	1603.15(2)	3972.08(5)
Z	4	4	4
ρ_{calc}g/cm^3	1.818	1.849	1.788
μ/mm^{-1}	2.609	2.657	4.806
F(000)	872.0	880.0	2120.0
Crystal size/mm^3	0.25×0.197×0.035	0.486×0.362×0.266	0.112×0.107×0.091
Radiation	CuKα (λ = 1.54184)	CuKα (λ = 1.54184)	CuKα (λ = 1.54184)
2Θ range for data collection/°	9.444 to 153.266	9.016 to 153.408	5.55 to 153.404
Index ranges	-12≤h≤12, -19≤k≤18, -14≤l≤13	-9≤h≤8, -22≤k≤22, -14≤l≤14	-22≤h≤22, -13≤k≤15, -22≤l≤24
Reflections collected	11711	8182	26207
Independent reflections	3367 [R_{int} = 0.0223, R_{sigma} = 0.0184]	3345 [R_{int} = 0.0155, R_{sigma} = 0.0166]	8292 [R_{int} = 0.0195, R_{sigma} = 0.0182]
Data/restraints/parameters	3367/0/278	3345/0/283	8292/0/588
Goodness-of-fit on F^2	1.058	1.074	1.034
Final R indexes [I> = 2σ (I)]	R_1 = 0.0325, wR_2 = 0.0829	R_1 = 0.0280, wR_2 = 0.0703	R_1 = 0.0298, wR_2 = 0.0744
Final R indexes [all data]	R_1 = 0.0339, wR_2 = 0.0840	R_1 = 0.0290, wR_2 = 0.0709	R_1 = 0.0316, wR_2 = 0.0758
Largest diff. peak/hole / e Å^{-3}	0.32/-0.31	0.32/-0.25	0.64/-0.69
CCDC	2061978	2061980	2061971

7.2.2. Crystallographic data and details of the structure refinements of compounds [Py(C_6F_5)$_2$PF][OTf] **3**[OTf], [(MeIm)(C_6F_5)$_2$PF][OTf] **4**[OTf] and [MeIm(C_6F_5)$_2$PF][OTf]-CO_2 adducts (**6**[OTf] and **7**[OTf]$_2$)

	**3**[OTf]·C_6H_5F	**4**[OTf]·CH_2Cl_2	**6**[OTf]·**7**[OTf]$_2$·CH_3NO_2
Empirical formula	$C_{18}H_4F_{14}NO_3PS$	$C_{104}H_{50}F_{62}N_8O_{12}P_4S_4$	$C_{54}H_{18}F_{42}N_7O_{15}P_3S_3$
Formula weight	611.25	3033.64	1991.84
Temperature/K	100.01(10)	100.01(10)	99.95(13)
Crystal system	monoclinic	monoclinic	monoclinic
Space group	$P2_1/c$	$P2_1/c$	$P2_1/n$
a/Å	11.44421(17)	14.6911(2)	15.5301(4)
b/Å	11.74021(17)	10.59320(10)	20.2798(4)
c/Å	15.6924(2)	18.3942(2)	25.3597(5)
α/°	90	90	90
β/°	95.6979(13)	97.2900(10)	101.443(2)
γ/°	90	90	90
Volume/Å^3	2097.97(5)	2839.47(6)	7828.2(3)
Z	4	1	4
ρ_{calc}g/cm^3	1.935	1.774	1.690
μ/mm-^{1}	3.533	2.842	2.969
F(000)	1200.0	1508.0	3928.0
Crystal size/mm^3	0.216×0.068×0.026	0.425×0.099×0.025	0.736 × 0.04 × 0.026
Radiation	CuKα (λ = 1.54184)	CuKα (λ = 1.54184)	CuKα (λ = 1.54184)
2Θ range for data collection/°	7.764 to 153.088	6.066 to 153.028	5.624 to 140.202
Index ranges	-12≤h≤14, -14≤k≤14, -19≤l≤18	-18≤h≤18, -12≤k≤13, -23≤l≤22	-18 ≤ h ≤ 18, -18 ≤ k ≤ 24, -30 ≤ l ≤ 30
Reflections collected	11020	23856	31861
Independent reflections	4359 [R_{int} = 0.0206, R_{sigma} = 0.0243]	5923 [R_{int} = 0.0251, R_{sigma} = 0.0193]	31861 [Rint = ?, Rsigma = 0.0789]
Data/restraints/parameters	4359/0/343	5923/94/499	31861/0/1121
Goodness-of-fit on F^2	1.011	1.060	1.019
Final R indexes [I> = 2σ (I)]	R_1 = 0.0319, wR_2 = 0.0781	R_1 = 0.0435, wR_2 = 0.1316	R1 = 0.0698, wR2 = 0.2040
Final R indexes [all data]	R_1 = 0.0387, wR_2 = 0.0815	R_1 = 0.0464, wR_2 = 0.1355	R1 = 0.1042, wR2 = 0.2138
Largest diff. peak/hole / e Å^{-3}	0.50/-0.37	1.20/-0.51	0.83/-0.47
CCDC	2061972	2061970	2061973

7.2.3. Crystallographic data and details of the structure refinements of compounds and [Py(C_6F_5)$_2$PF(Me_2CO)][OTf] **8a**[OTf], [MeIm(C_6F_5)$_2$PF(Me_2CO)][OTf] **8b**[OTf] and [Py(C_6F_5)$_2$PF(MeCN)][OTf] **9a**[OTf].

	**8a**[OTf]·CH_2Cl_2	**8b**[OTf]·CH_2Cl_2	**9a**[OTf]·$(CH_2Cl_2)_2$·CH_3CN
Empirical formula	$C_{21.5}H_{11}ClF_{14}NO_4PS$	$C_{20.5}H_{12}ClF_{14}N_2O_4PS$	$C_{21.24}H_{8.49}Cl_{1.3}F_{14}N_{2.58}O_3PS$
Formula weight	711.79	714.80	722.98
Temperature/K	100.01(10)	100.01(10)	100.01(10)
Crystal system	monoclinic	monoclinic	monoclinic
Space group	$P2_1/c$	$P2_1/n$	$C2/c$
a/Å	8.90719(10)	9.0162(4)	34.458(2)
b/Å	10.01123(9)	9.9408(3)	8.4701(3)
c/Å	28.6690(3)	29.4074(9)	25.1477(18)
α/°	90	90	90
β/°	95.1292(9)	97.671(3)	131.606(11)
γ/°	90	90	90
Volume/Å^3	2546.23(4)	2612.15(16)	5488.1(9)
Z	4	4	8
ρ_{calc}g/cm^3	1.857	1.818	1.750
μ/m*m*$^{-1}$	3.989	3.901	3.969
F(000)	1412.0	1420.0	2857.0
Crystal size/mm^3	0.313×0.204×0.132	0.262×0.135×0.097	0.323×0.098×0.025
Radiation	CuKα (λ = 1.54184)	Cu Kα (λ = 1.54184)	CuKα (λ = 1.54184)
2Θ range for data collection/°	6.19 to 153.596	6.066 to 136.476	6.862 to 153.648
Index ranges	-9≤h≤11, -7≤k≤12, -35≤l≤36	-10 ≤ h ≤ 10, -11 ≤ k ≤ 11, -35 ≤ l ≤ 21	-33≤h≤43, -10≤k≤10, -31≤l≤30
Reflections collected	13764	13706	14788
Independent reflections	5301 [R_{int} = 0.0202, R_{sigma} = 0.0234]	4778 [R_{int} = 0.0514, R_{sigma} = 0.0504]	5701 [R_{int} = 0.0264, R_{sigma} = 0.0311]
Data/restraints/parameters	5301/0/408	4778/31/409	5701/68/454
Goodness-of-fit on F^2	1.081	1.027	1.051
Final R indexes [I> = 2σ (I)]	R_1 = 0.0398, wR_2 = 0.1017	R_1 = 0.0642, wR_2 = 0.1767	R_1 = 0.0534, wR_2 = 0.1478
Final R indexes [all data]	R_1 = 0.0408, wR_2 = 0.1025	R_1 = 0.0830, wR_2 = 0.1953	R_1 = 0.0585, wR_2 = 0.1527
Largest diff. peak/hole / e Å^{-3}	0.44/-0.56	0.65/-0.63	0.74/-0.65
CCDC	2061974	2061975	2061976

7.2.4. Crystallographic data and details of the structure refinements of compounds [(MeIm)(C_6F_5)$_2$PF(MeCN)][OTf] **9b**[OTf] and [(MeIm)(C_6F_5)$_2$PF(PhCCH)][OTf] **12b**[OTf].

	**9b**[OTf]	**12b**[OTf]
Empirical formula	$C_{19}H_8F_{14}N_3O_3PS$	$C_{200}H_{88}F_{112}N_{16}O_{24}P_8S_8$
Formula weight	655.31	5731.10
Temperature/K	100.01(10)	100.01(10)
Crystal system	monoclinic	orthorhombic
Space group	$P2_1/n$	$Pna2_1$
a/Å	10.1745(3)	18.74170(10)
b/Å	16.3686(4)	8.58340(10)
c/Å	13.6402(4)	33.7232(3)
α/°	90	90
β/°	93.848(3)	90
γ/°	90	90
Volume/Å^3	2266.56(11)	5424.97(8)
Z	4	1
ρ_{calc}g/cm^3	1.920	1.754
μ/m*m*$^{-1}$	3.347	2.851
F(000)	1296.0	2848.0
Crystal size/mm^3	0.222×0.18×0.108	0.132 × 0.095 × 0.051
Radiation	CuKα (λ = 1.54184)	Cu Kα (λ = 1.54184)
2Θ range for data collection/°	8.45 to 152.916	5.242 to 153.874
Index ranges	-9≤h≤12, -20≤k≤18, -17≤l≤15	-23 ≤ h ≤ 17, -10 ≤ k ≤ 10, -42 ≤ l ≤ 41
Reflections collected	11420	35899
Independent reflections	4665 [R_{int} = 0.0244, R_{sigma} = 0.0226]	10860 [R_{int} = 0.0428, R_{sigma} = 0.0317]
Data/restraints/parameters	4665/0/372	10860/1/832
Goodness-of-fit on F^2	1.068	1.032
Final R indexes [I> = 2σ (I)]	R_1 = 0.0497, wR_2 = 0.1390	R_1 = 0.0559, wR_2 = 0.1459
Final R indexes [all data]	R_1 = 0.0546, wR_2 = 0.1440	R_1 = 0.0578, wR_2 = 0.1487
Largest diff. peak/hole / e Å^{-3}	0.65/-0.59	1.56/-0.46
CCDC	2061977	2061979

8 Computational Methods

All geometry optimizations have been carried out using Turbomole 7.2[7] in the gas phase at the BP86-D3/def2-TZVP level of theory[8]. Energies were subsequently recalculated on the optimized structures at the MP2/def2-TZVP level of theory[9]. NMR calculations were computed using Gaussian 09[10] calculation package at the PB86/def2-TZVP level of theory. Solvents effects were taken into account by means of the PCM (Polarized Continum Model)[11] module using chloroform as solvent. The global electrophilicity index (GEI) and fluoride ion affinity (FIA) are calculated based on the literature reported.[12]

The GEI (ω/eV) values are calculated based on the chemical potential μ, and chemical hardness η, as depicted in Scheme 1 **I-III**.

GEI:

(I) $\omega = \mu^2/2\eta$

(II) $\mu = -(E_{HOMO} + E_{LUMO})/2$

(III) $\eta = (E_{LUMO} - E_{LUMO})$

FIA:

(IV) $R_3PF^+ + F^- \longrightarrow R_3PF_2 \quad \Delta H = -FIA$

(V) $F_2CO + F^- \longrightarrow F_3CO^- \quad \Delta H = -209$ KJ/mol

Scheme **1.** Method used for calculating GEI and FIA values.

The FIA value is defined as the negative of energy change for reaction **IV**. Due to the difficulties of obtaining accurate energies for the F^- anion, the energy of the F^- anion (-261834.54 kJ/mol) is obtained by the combination of the experimentally obtained energy change for reaction **V** (-209 kJ/mol) with the calculation energies of F_2CO (-820803.50 kJ/mol) and F_3CO^- (-1082847.04 kJ/mol). The calculated GEI and FIA are shown in Table 1 and the optimized structure of **3**$^+$-CO_2_A/B and **4**$^+$-CO_2_A/B, **11a**$^{2+}$, and **12a**$^+$ are listed below.

Table 1. Results of DFT calculations.

	**Energy** (kJ/mol)	**Energy of F⁻ adduct** (kJ/mol)	**HOMO** (eV)	**LUMO** (eV)	**GEI** (eV)	**FIA** (kJ/mol)
**F_2CO**	-820803.50	-1082847.04				
**3**$^+$	-5621533.46	-5884116.01	-13.728	-2.859	3.164	748.0
**4**$^+$	-5666783.67	-5929362.05	-13.127	-2.874	3.121	743.8
**11a**$^{2+}$	-6269759.7	-6532612.02	-14.662	-6.852	7.408	1017.8

Optimazed structures of **3**$^+$-CO_2_A/B and **4**$^+$-CO_2_A/B:

3$^+$-CO_2_A

nmr=giao scrf=(solvent=chloroform) bp86/def2tzvp *ΔE* = -4.9 kcal/mol *Δδ*(^{31}P) = -124 ppm

P	-0.01563500	0.45030100	-0.37704900
F	-0.11701900	0.30412900	-2.00140400
N	0.10151900	2.90903700	0.68918200
C	0.03241700	2.29019600	-0.50811000
C	0.01718000	3.04641600	-1.67127800
H	-0.04860100	2.55085400	-2.63680500
C	0.08540400	4.44218900	-1.56026200
H	0.08003500	5.05562500	-2.46152500
C	0.16107200	5.04513000	-0.30163300
H	0.21704400	6.12706300	-0.19375600
C	0.16525600	4.24452800	0.83476200
H	0.21910800	4.60373100	1.86293000
C	-1.59086100	-0.37575300	-0.07214400
C	-1.69610300	-1.48338200	0.78361800
C	-2.91556500	-2.13354900	0.97329100
C	-4.06060400	-1.66042100	0.31883600
C	-3.98206900	-0.54701500	-0.52802200
C	-2.75100100	0.07801100	-0.71727400
C	1.54437000	-0.45069000	-0.27115000
C	1.63716500	-1.75112500	-0.79219900
C	2.83715900	-2.46193700	-0.75874400
C	3.97770300	-1.86221000	-0.20984000
C	3.91274500	-0.56081800	0.30530200
C	2.70207200	0.12624700	0.26814000
F	2.68076000	1.39680400	0.72461800
F	5.00327200	0.01701600	0.80603300
F	5.12712200	-2.52279800	-0.18642200
F	2.90215600	-3.69912400	-1.24429000
F	0.55626000	-2.34855300	-1.31014100
F	-2.70118800	1.16735200	-1.50779300

F	-5.07585700	-0.08801100	-1.13485600
F	-5.22544400	-2.26689900	0.50523100
F	-2.99488700	-3.20052200	1.76454700
F	-0.61336900	-1.96425500	1.40686100
C	0.12339700	1.97288100	1.90990800
O	0.16470400	2.44813400	3.00527100
O	0.08300000	0.75643100	1.47472400

3^{+}-CO_2_B

nmr=giao scrf=(solvent=chloroform) bp86/def2tzvp ΔE = -4.8 kcal/mol Δδ(^{31}P) = -17 ppm

P	-0.6303477	0.0656197	-0.8742735
F	-1.6611836	0.2648504	-2.0453316
N	1.5859972	-1.3613248	-0.9779781
C	0.2874721	-1.4221141	-1.3008510
C	-0.3531855	-2.5098603	-1.9020257
H	-1.4154761	-2.4881274	-2.1471379
C	0.4377699	-3.6253227	-2.1881315
H	-0.0001613	-4.5024677	-2.6644366
C	1.7924979	-3.5922622	-1.8563722
H	2.4390965	-4.4442858	-2.0656299
C	2.3243823	-2.4429503	-1.2538863
H	3.3816443	-2.3844023	-0.9885670
C	0.4237891	1.4898118	-0.8398040
C	0.5729691	2.3538753	0.2640758
C	1.4563836	3.4289257	0.2244216
C	2.2111596	3.6630396	-0.9330729
C	2.0872115	2.8198402	-2.0477648
C	1.2038729	1.7477202	-1.9880601
C	-1.6358952	-0.0956007	0.5826005
C	-2.6360737	0.8696988	0.8229819
C	-3.4513344	0.8104862	1.9468802
C	-3.2635720	-0.2293548	2.8717196
C	-2.2746873	-1.2010684	2.6633470
C	-1.4716069	-1.1289794	1.5259277
F	-0.5169725	-2.0554037	1.3695283
F	-2.1010832	-2.1736955	3.5509135
F	-4.0222446	-0.2862286	3.9529441
F	-4.3858450	1.7307208	2.1582102
F	-2.7878472	1.8867778	-0.0348755
F	1.0876828	0.9477372	-3.0560037
F	2.8057121	3.0521872	-3.1417504
F	3.0444906	4.6890628	-0.9754811

F	1.5844818	4.2331345	1.2765397
F	-0.1205573	2.1690225	1.3956598
C	1.3410023	-0.0922385	2.8981261
O	1.9885644	0.1401656	1.9487925
O	0.6718935	-0.3269888	3.8287655

4^{+}-CO_2_A

nmr=giao scrf=(solvent=chloroform) bp86/def2tzvp ΔE = -5.5 kcal/mol Δδ(^{31}P) = -108 ppm

P	0.0793985	-0.0276601	-0.0144131
F	1.6691356	0.1357625	0.3711977
F	-0.8292900	-2.9036797	-0.5787250
F	-0.2888110	-4.3991407	-2.7698053
F	1.2725113	-3.3875319	-4.7681365
F	2.2874749	-0.8622427	-4.5821079
F	1.7466464	0.6597684	-2.4079239
F	0.6539303	2.1431598	1.9794884
F	0.3232866	4.8146585	1.6975191
F	-0.7053781	5.8300898	-0.6220519
F	-1.4124586	4.1616041	-2.6619721
F	-1.0969315	1.4891783	-2.4022094
N	0.4255686	-1.0539511	2.7428570
N	-1.5423093	-1.0853295	1.8235261
C	-0.2566859	-0.7557347	1.6161126
C	-0.4694837	-1.5817488	3.6583003
H	-0.1494956	-1.8976153	4.6448421
C	-1.7167921	-1.6025201	3.0767301
H	-2.6834128	-1.9312232	3.4404288
C	1.8605147	-0.8473754	3.0013641
H	2.4497213	-1.4211636	2.2794662
H	2.0730731	-1.1898067	4.0178007
H	2.0952107	0.2167169	2.9018731
C	0.4189362	-1.0669327	-1.4497459
C	-0.0906132	-2.3673874	-1.5710774
C	0.1888622	-3.1588143	-2.6834231
C	0.9964109	-2.6428492	-3.7062547
C	1.5238995	-1.3491896	-3.6065198
C	1.2412302	-0.5797591	-2.4782559
C	-0.1923996	1.7435684	-0.2151523
C	0.1670365	2.6241703	0.8150956
C	-0.0060270	4.0008504	0.6950312
C	-0.5429081	4.5205549	-0.4905504
C	-0.9133398	3.6623211	-1.5342675

C	-0.7489684	2.2849531	-1.3844673
C	-2.4622790	-0.8168246	0.6877904
O	-3.6289230	-1.0615819	0.7551124
O	-1.7363405	-0.2972942	-0.2774761

4^{+}-CO_2_B

nmr=giao scrf=(solvent=chloroform) bp86/def2tzvp ΔE = -5.8 kcal/mol Δδ(^{31}P) = -10 ppm

P	0.8150593	0.3149874	0.3006723
F	2.2919040	0.6394129	0.7524742
F	-0.4455322	-2.4781001	-0.1585188
F	-0.1357163	-4.0372520	-2.3279498
F	1.4756318	-3.2453996	-4.3734939
F	2.7980257	-0.8646328	-4.2606337
F	2.4794634	0.7503639	-2.0933615
F	0.4884969	2.6243204	2.0760582
F	-0.8368175	4.9435863	1.6179265
F	-1.9471206	5.4083029	-0.8253143
F	-1.7521341	3.5581279	-2.8141536
F	-0.4582670	1.2478254	-2.3953773
N	0.4305696	-1.3969768	2.5029279
N	-1.2291175	0.0530275	2.0362798
C	-0.0059080	-0.3696745	1.6902393
C	-0.5748371	-1.6180046	3.3997721
H	-0.4867458	-2.3724014	4.1749047
C	-1.5905867	-0.7164260	3.0955171
H	-2.5495780	-0.5938380	3.5886982
C	1.6846616	-2.1458351	2.4055628
H	1.6561076	-2.8157248	1.5363743
H	1.8100138	-2.7446030	3.3127068
H	2.5305340	-1.4543649	2.3190726
C	1.0241877	-0.8124920	-1.0583277
C	0.3506742	-2.0476820	-1.1462389
C	0.5004883	-2.8705220	-2.2614977
C	1.3333096	-2.4661842	-3.3143213
C	2.0189176	-1.2424539	-3.2528768
C	1.8560663	-0.4342226	-2.1329978
C	0.0397418	1.8467225	-0.1289031
C	-0.0697060	2.8317028	0.8789822
C	-0.7397417	4.0298066	0.6571854
C	-1.3130536	4.2693851	-0.6003820
C	-1.2100824	3.3168058	-1.6234542
C	-0.5360905	2.1210432	-1.3876150

C	-3.2331186	-0.4075321	-0.0576004
O	-4.0780524	-0.5464812	0.7348327
O	-2.3916472	-0.2746169	-0.8671691

Optimazed structure of **11a**$^{2+}$ and **12a**$^{+}$:

11a^{2+}

MP2/def2-TZVP

P	-0.1171860	0.0478775	1.3765420
C	-0.5642334	1.6559704	2.0116600
F	-1.7757542	-0.7131011	3.6950349
F	0.6891080	1.4140518	4.0332990
F	-1.5702236	5.4635308	3.2772535
C	-3.0339028	-1.9005105	-1.5382244
H	-3.6194499	-2.3960656	-2.3114509
C	0.6579158	0.5178389	-4.8331952
H	0.0628433	0.9548463	-5.6361456
N	-1.0222762	-0.7913352	-0.9230046
F	2.5149555	-1.4840017	1.7829203
F	-1.7920410	2.0769731	0.0138140
C	0.3393449	-1.0781113	2.6797314
C	-1.7643628	-1.4293601	-1.8585947
H	-1.3070277	-1.5532646	-2.8380234
F	-2.4716365	4.5603261	0.8680711
C	2.2052374	-0.5247106	-2.7507007
H	2.8041968	-0.9501995	-1.9442529
C	-1.4701069	-0.6682718	0.3696276
C	-0.5675474	-1.3129380	3.7315299
C	1.9963660	0.1551393	-5.0835427
C	-3.5241388	-1.7446220	-0.2432061
H	-4.5180982	-2.1068981	0.0207766
C	2.7523061	-0.3717373	-4.0147527
H	3.7870271	-0.6704761	-4.1892522
C	0.0862673	0.3491306	-3.5803813
H	-0.9349098	0.6967149	-3.4116079
C	0.9507940	0.1286730	-0.0208642
H	1.9257026	0.6062574	-0.0057631
F	0.0046192	3.8882537	4.8450922
C	-1.2496282	4.2516993	2.8745381
F	-1.1168499	-2.3290500	5.7896384
C	1.9565410	-2.5018464	3.8249673
F	3.1547154	-3.0635364	3.8832906
F	1.3546286	-3.4792403	5.8799861

C	-0.2464827	-2.1223685	4.8126560
C	1.0290220	-2.7165194	4.8579490
C	-1.7141206	3.7855734	1.6318828
C	-1.3594100	2.5077879	1.2158244
C	0.8539273	-0.1806498	-2.5145813
C	-0.4398000	3.4372102	3.6823121
C	0.3313263	-0.2641463	-1.1675701
C	-0.1009338	2.1552692	3.2486326
C	2.6129160	0.3445153	-6.4336065
H	1.8626227	0.5424220	-7.2075501
H	3.2068143	-0.5325328	-6.7268293
H	3.3076361	1.2006885	-6.4116953
C	1.6081179	-1.6898987	2.7445743
C	-2.7167313	-1.1354764	0.7325570
H	-3.0580999	-1.0298811	1.7606338

12a$^+$

MP2/def2-TZVP

P	0.6759990	0.1218966	11.4556906
C	-0.3191321	1.5603252	11.9922776
F	-1.1953044	-0.7788623	13.8013917
F	0.4391765	1.4032424	14.2496435
F	-2.5055840	4.9001122	13.1458074
C	-2.5635133	-2.0271130	8.7627718
H	-3.2042818	-2.5663380	8.0677584
C	0.9105586	0.0850998	5.0793309
H	0.2878371	0.5168707	4.2941153
N	-0.5044560	-0.9342048	9.2014322
F	2.7373896	-2.0685390	11.4302365
F	-1.1455975	1.9096718	9.7768838
C	0.7587810	-1.3540670	12.5514670
C	-1.3106955	-1.6175442	8.3454502
H	-0.9133207	-1.8082417	7.3517891
F	-2.5585758	4.0696209	10.5407224
C	2.5131083	-0.9675826	7.1061764
H	3.1363017	-1.3858492	7.8975864
C	-0.8393084	-0.6867273	10.5026825
C	-0.1497382	-1.6072984	13.5857285
C	2.1788399	-0.4266718	4.7525616
C	-2.9761603	-1.7329696	10.0656153
H	-3.9696793	-2.0252081	10.4063574
C	2.9661989	-0.9526247	5.7899585

H	3.9520425	-1.3600742	5.5605871
C	0.4395811	0.0662095	6.3872374
H	-0.5327191	0.5072642	6.6154573
C	1.4864740	0.1043041	9.8511425
H	2.4665223	0.5566054	9.7355141
F	-0.9990364	3.5528068	14.9836772
C	-1.8025212	3.8344907	12.7767301
F	-0.8940215	-2.8996584	15.4246694
C	1.9821113	-3.3589568	13.2388214
F	2.9988674	-4.2042513	13.0619817
F	1.1961488	-4.6289385	15.0730285
C	-0.0123504	-2.6975322	14.4437156
C	1.0592451	-3.5803213	14.2676559
C	-1.8369084	3.4033145	11.4462669
C	-1.1032204	2.2782680	11.0779886
C	1.2376187	-0.4668790	7.4199458
C	-1.0257429	3.1452209	13.7146286
C	0.8047815	-0.4163623	8.8168747
C	-0.2847143	2.0306853	13.3159059
C	2.6856866	-0.3776613	3.3385736
H	1.8704364	-0.5018639	2.6135005
H	3.4423779	-1.1495014	3.1505676
H	3.1539102	0.5991475	3.1373526
C	1.8262513	-2.2522388	12.4021285
C	-2.1016882	-1.0757962	10.9343940
H	-2.4014114	-0.8570722	11.9562206
F	2.0429299	0.7502650	12.1644684

9 Reference

1. Brinkmann, Y.; Madhushaw, R. J.; Jazzar, R.; Bernardinelli, G.; Kündig, E. P., Chiral ruthenium Lewis acid-catalyzed nitrile oxide cycloadditions. *Tetrahedron* **2007,** *63* (35), 8413-8419.
2. Schoemaker, R.; Schwedtmann, K.; Franconetti, A.; Frontera, A.; Hennersdorf, F.; Weigand, J. J., Controlled scrambling reactions to polyphosphanes via bond metathesis reactions. *Chem Sci* **2019,** *10* (48), 11054-11063.
3. Guo, C.-X.; Yogendra, S.; Gomila, R. M.; Frontera, A.; Hennersdorf, F.; Steup, J.; Schwedtmann, K.; Weigand, J. J., A Convenient Access to Fluorophosphonium Triflate Salts by Electrophilic Fluorination and Anion Exchange. *Inorg. Chem. Front.* **2021**, DOI: 10.1039/D1QI00322D.

4. Dolomanov, O. V.; Bourhis, L. J.; Gildea, R. J.; Howard, J. A. K.; Puschmann, H., OLEX2: a complete structure solution, refinement and analysis program. *J Appl Crystallogr* **2009,** *42* (2), 339-341.
5. Sheldrick, G., A short history of SHELX. *Acta Crystallogr A* **2008,** *64* (1), 112-122.
6. Sheldrick, G., SHELXT - Integrated space-group and crystal-structure determination. *Acta Crystallogr A* **2015,** *71* (1), 3-8.
7. Spek, A., PLATON SQUEEZE: a tool for the calculation of the disordered solvent contribution to the calculated structure factors. *Acta Crystallographica Section C* **2015,** *71* (1), 9-18.
8. a: Becke, A. D., Density-functional exchange-energy approximation with correct asymptotic behavior. *Phys Rev A Gen Phys* **1988,** *38* (6), 3098-3100; b: Perdew, J. P., Density-functional approximation for the correlation energy of the inhomogeneous electron gas. *Phys Rev B Condens Matter* **1986,** *33* (12), 8822-8824; c: Weigend, F.; Ahlrichs, R., Balanced basis sets of split valence, triple zeta valence and quadruple zeta valence quality for H to Rn: Design and assessment of accuracy. *Phys Chem Chem Phys* **2005,** *7* (18), 3297-305; d: Weigend, F., Accurate Coulomb-fitting basis sets for H to Rn. *Phys Chem Chem Phys* **2006,** *8* (9), 1057-65.
9. a: Frisch, M. J.; Headgordon, M.; Pople, J. A., A Direct Mp2 Gradient-Method. *Chem Phys Lett* **1990,** *166* (3), 275-280; b: Sæbø, S.; Almlöf, J., Avoiding the integral storage bottleneck in LCAO calculations of electron correlation. *Chem Phys Lett* **1989,** *154* (1), 83-89; c: Head-Gordon, M.; Pople, J. A.; Frisch, M. J., MP2 energy evaluation by direct methods. *Chem Phys Lett* **1988,** *153* (6), 503-506.

10 NMR spectra

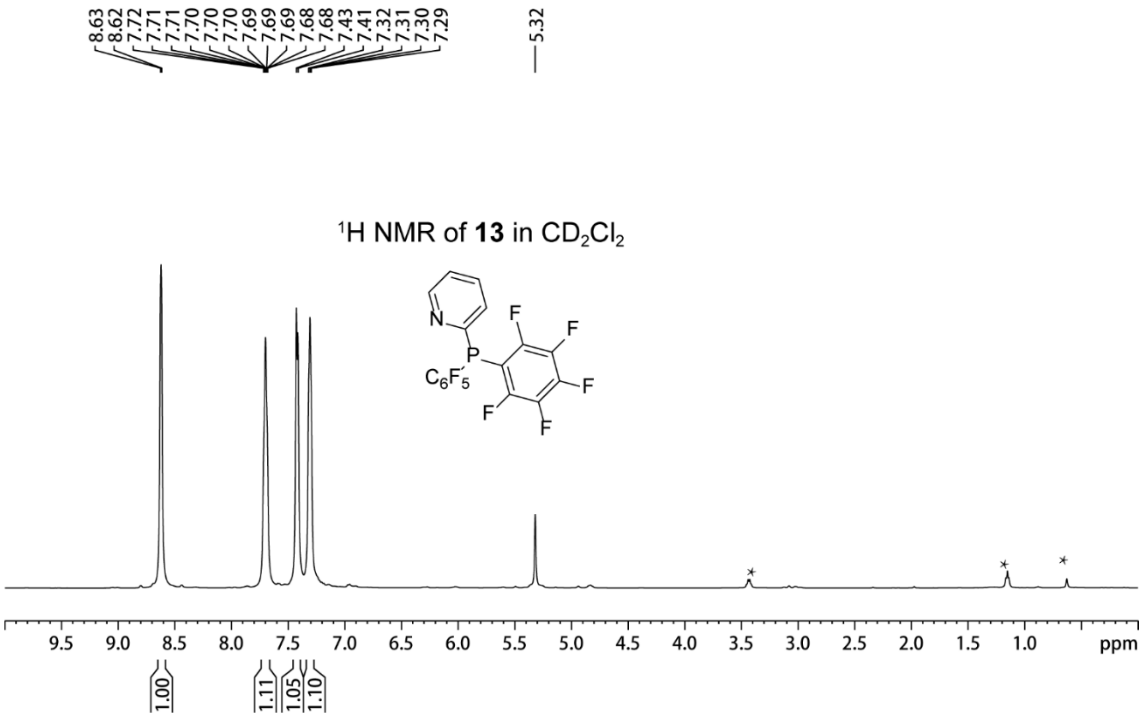

Figure **S12**. ^{1}H NMR spectrum of **13** (CD_2Cl_2, 300 K); Asterisks indicate small amounts of solvent residue.

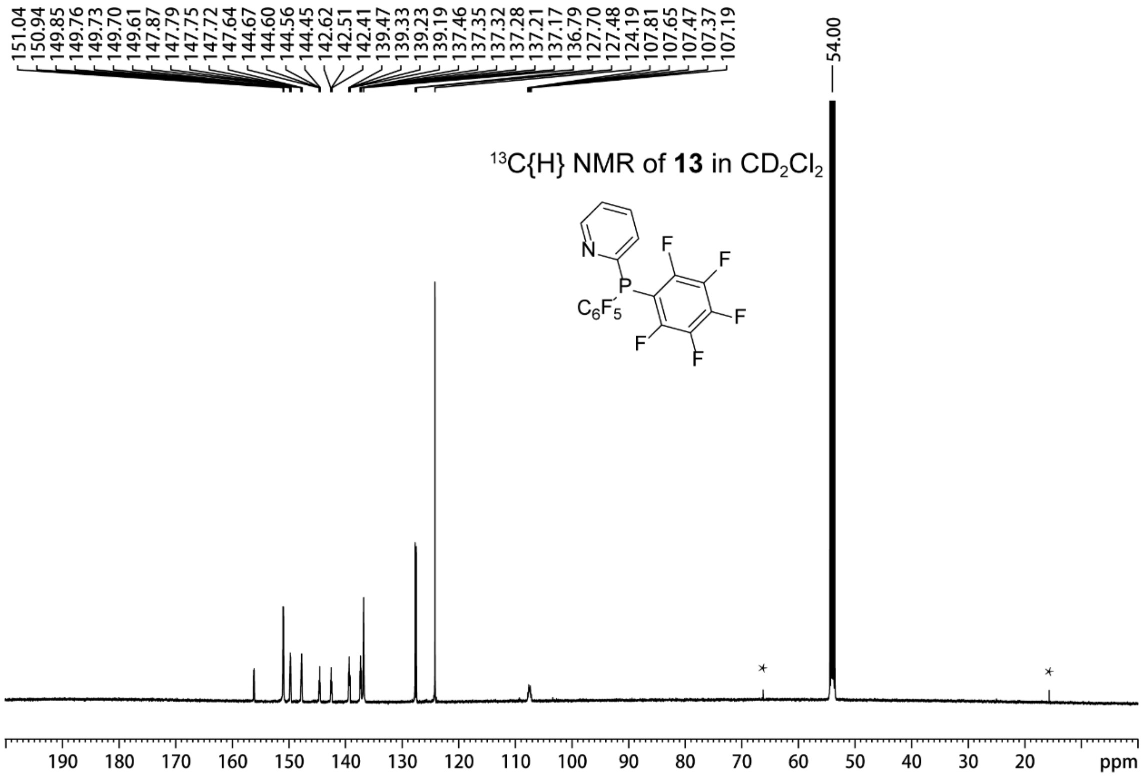

Figure **S13**. $^{13}C\{^{1}H\}$ NMR spectrum of **13** (CD_2Cl_2, 300 K); Asterisks indicate small amounts of solvent residue.

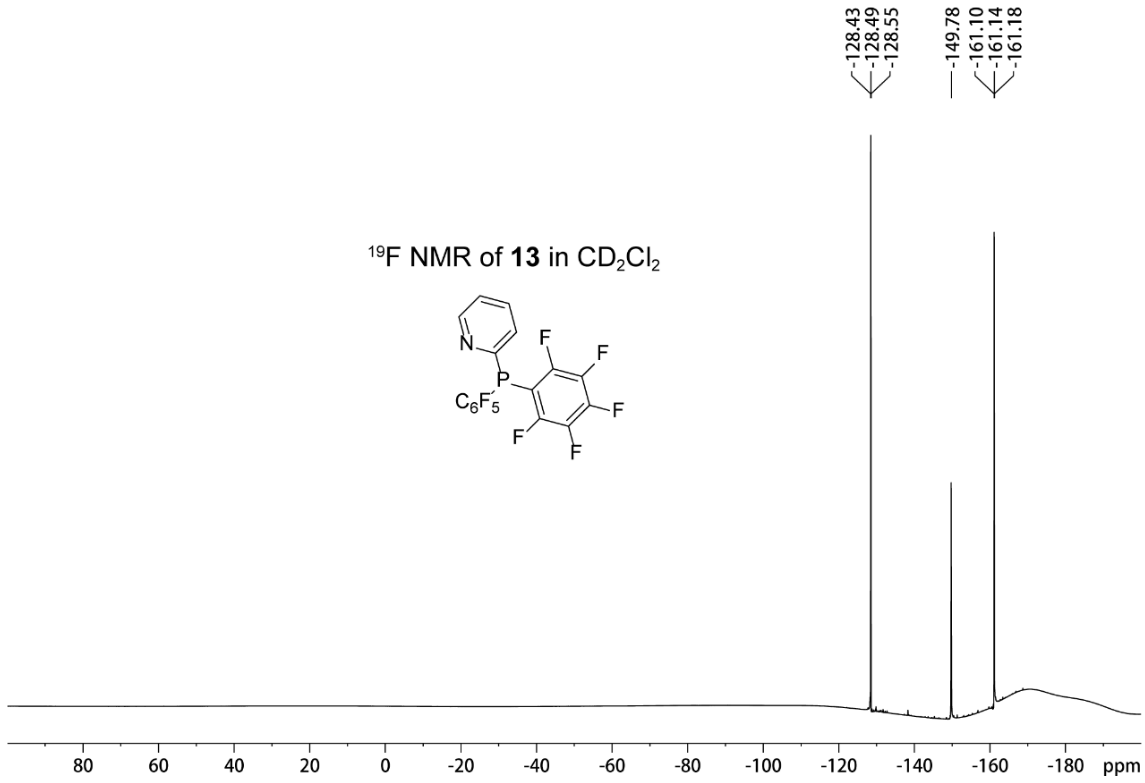

Figure **S14**. ${}^{19}F$ NMR spectrum of **13** (CD_2Cl_2, 300 K).

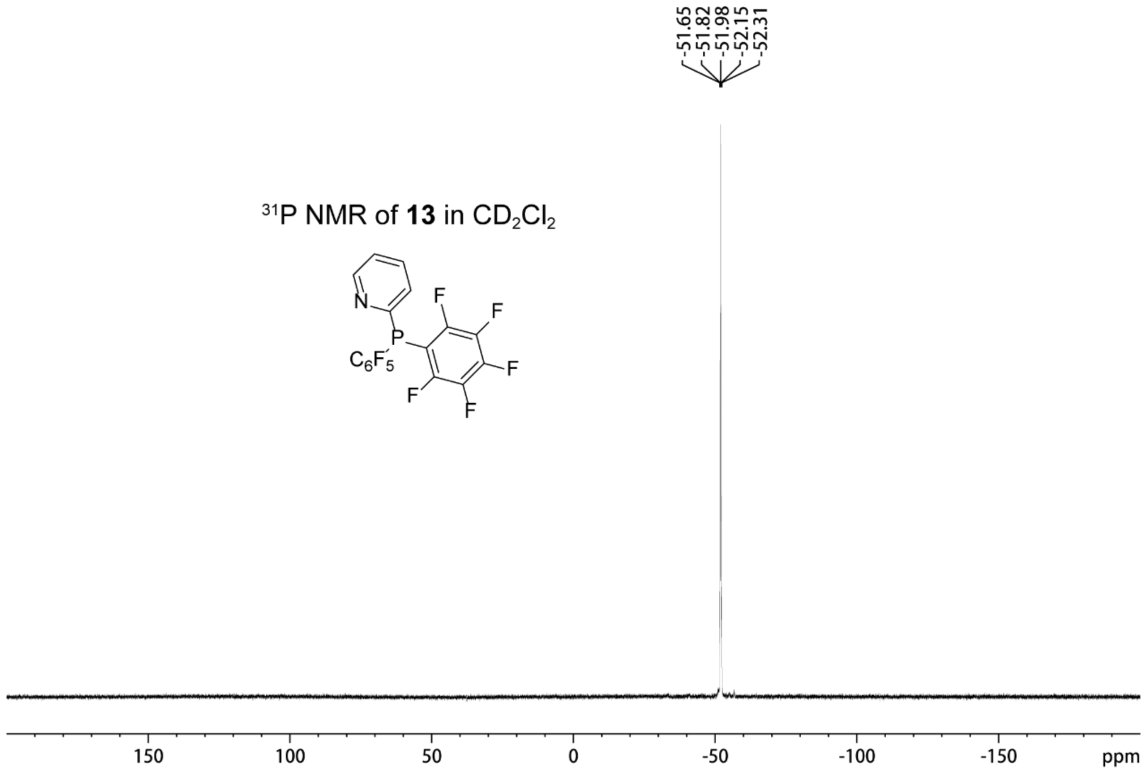

Figure **S15**. ${}^{31}P\{H\}$ NMR spectrum of **13** (CD_2Cl_2, 300 K).

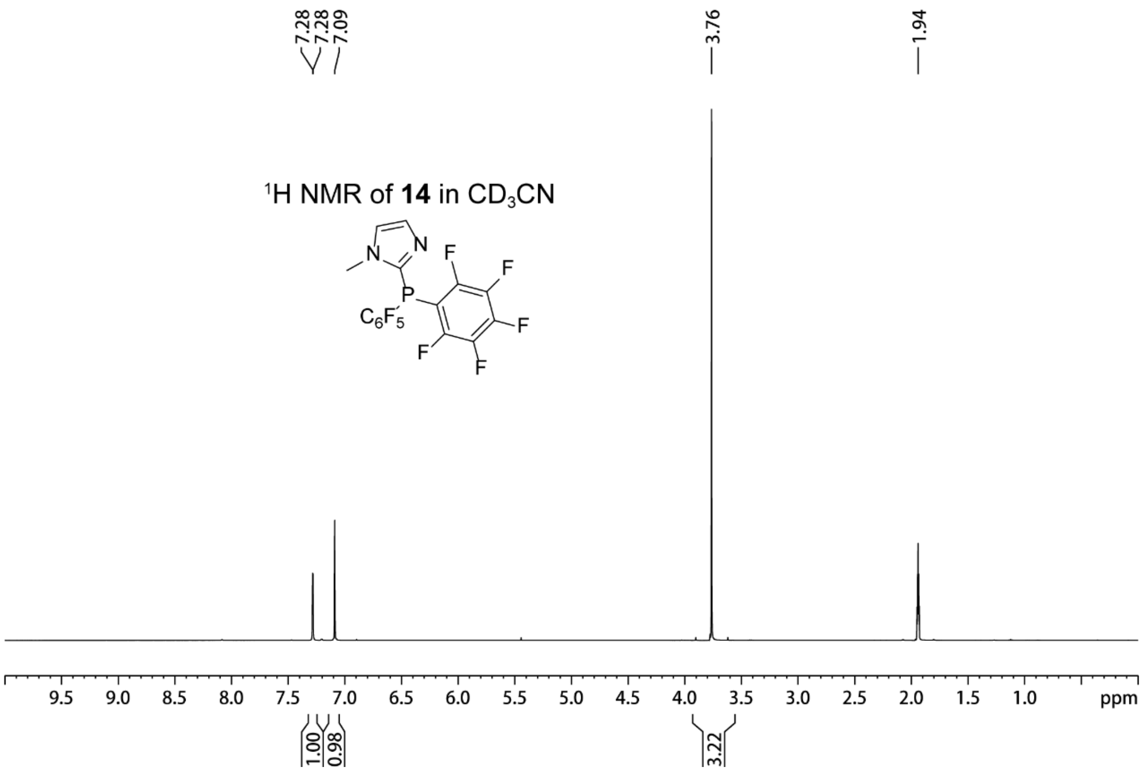

Figure **S16**. ^{1}H NMR spectrum of **14** (CD_3CN, 300 K).

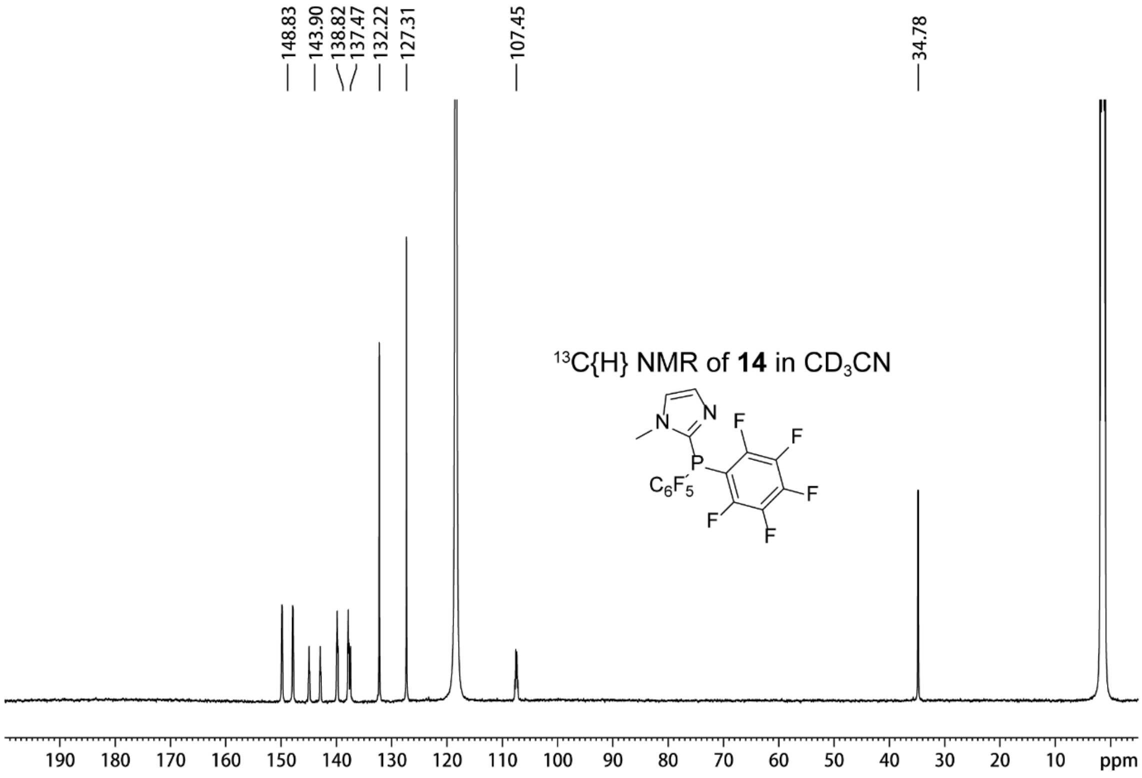

Figure **S17**. $^{13}C\{^1H\}$ NMR spectrum of **14** (CD_3CN, 300 K).

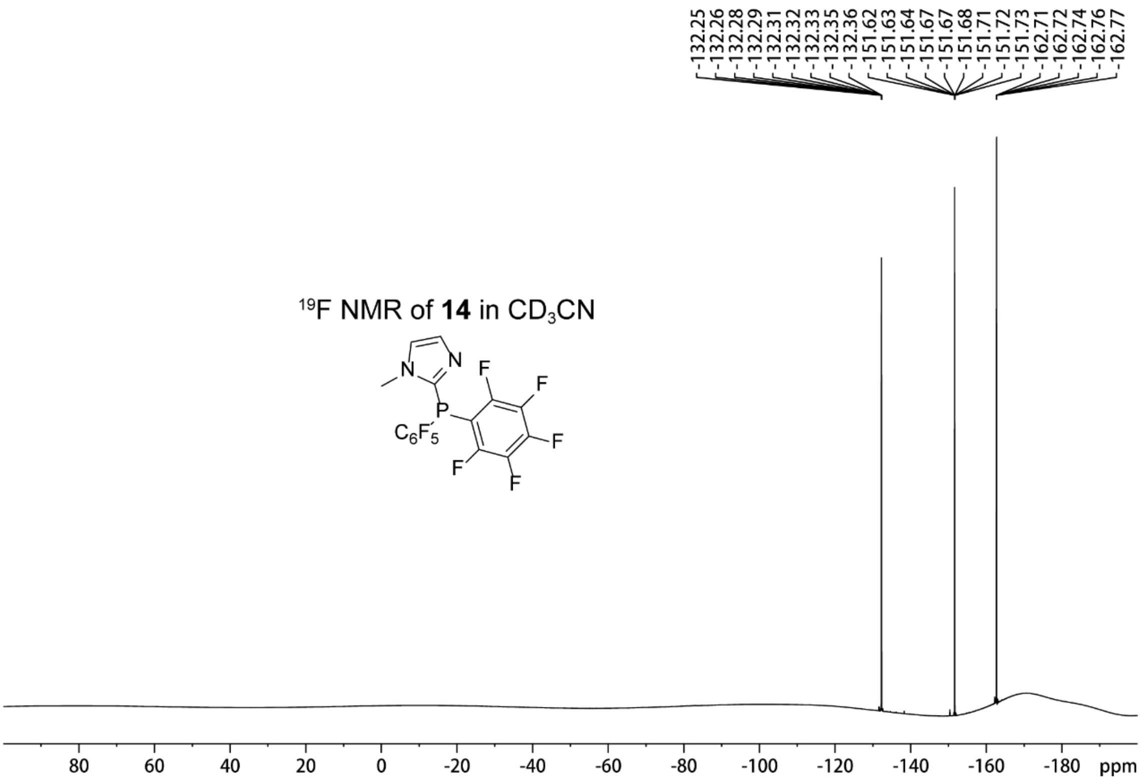

Figure **S18**. ^{19}F NMR spectrum of **14** (CD_3CN, 300 K).

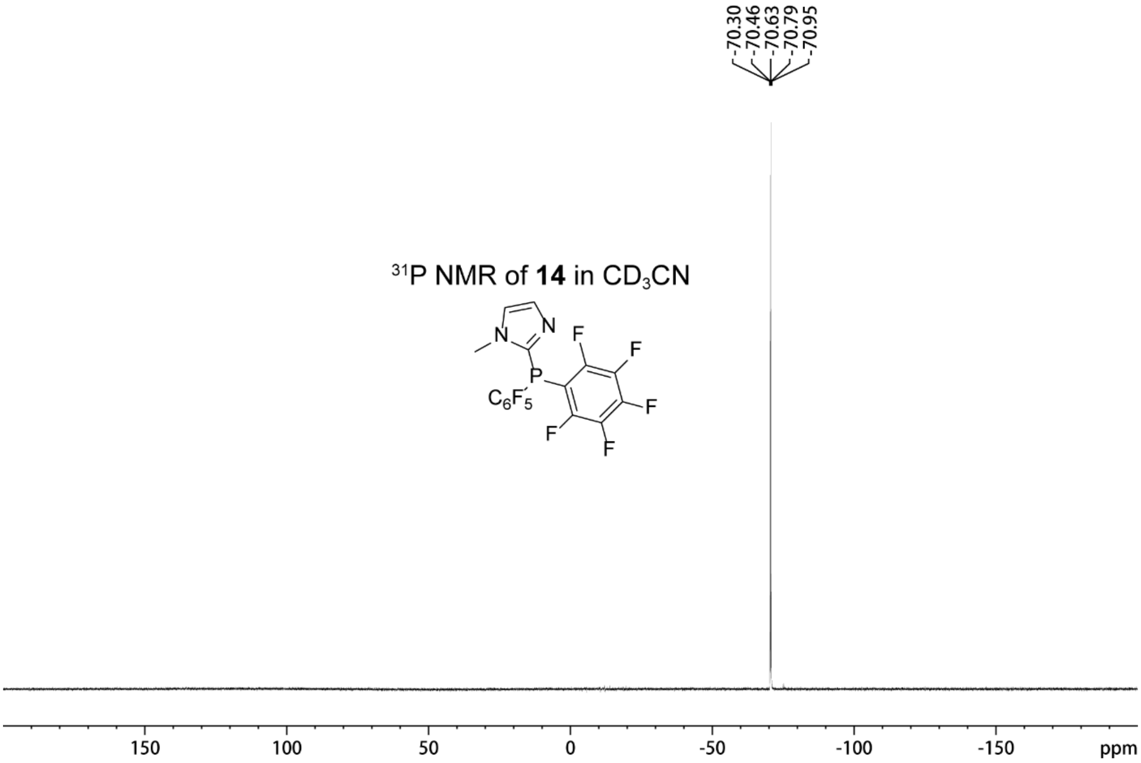

Figure **S19**. $^{31}P\{H\}$ NMR spectrum of **14** (CD_3CN, 300 K).

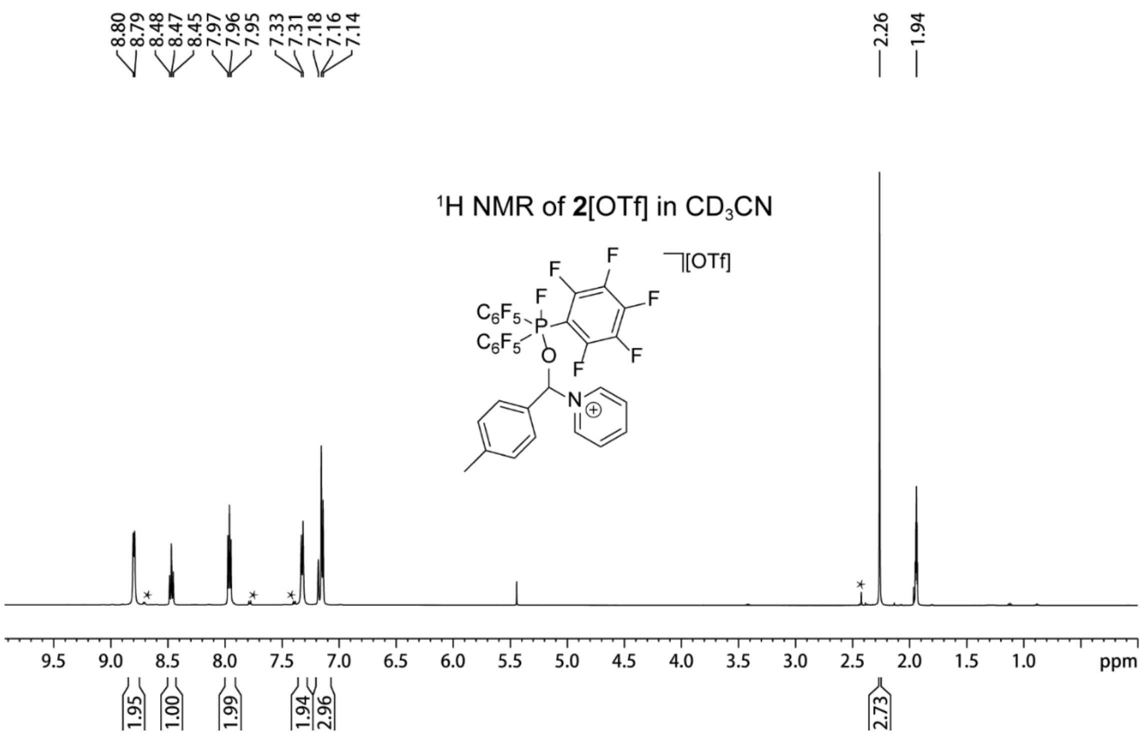

Figure **S20**. ¹H NMR spectrum of **2**[OTf] (CD_3CN, 300 K); Asterisks indicate minor amounts of unidentified side products.

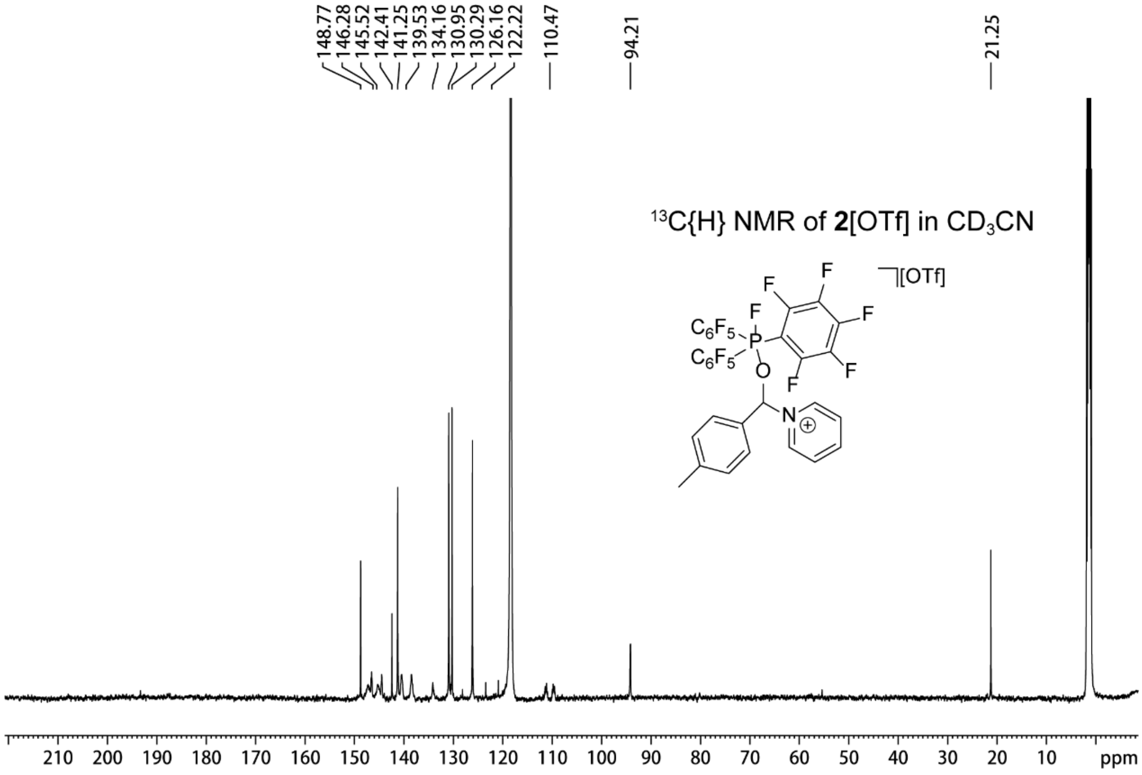

Figure **S21**. ¹³C{H} NMR spectrum of **2**[OTf] (CD_3CN, 300 K).

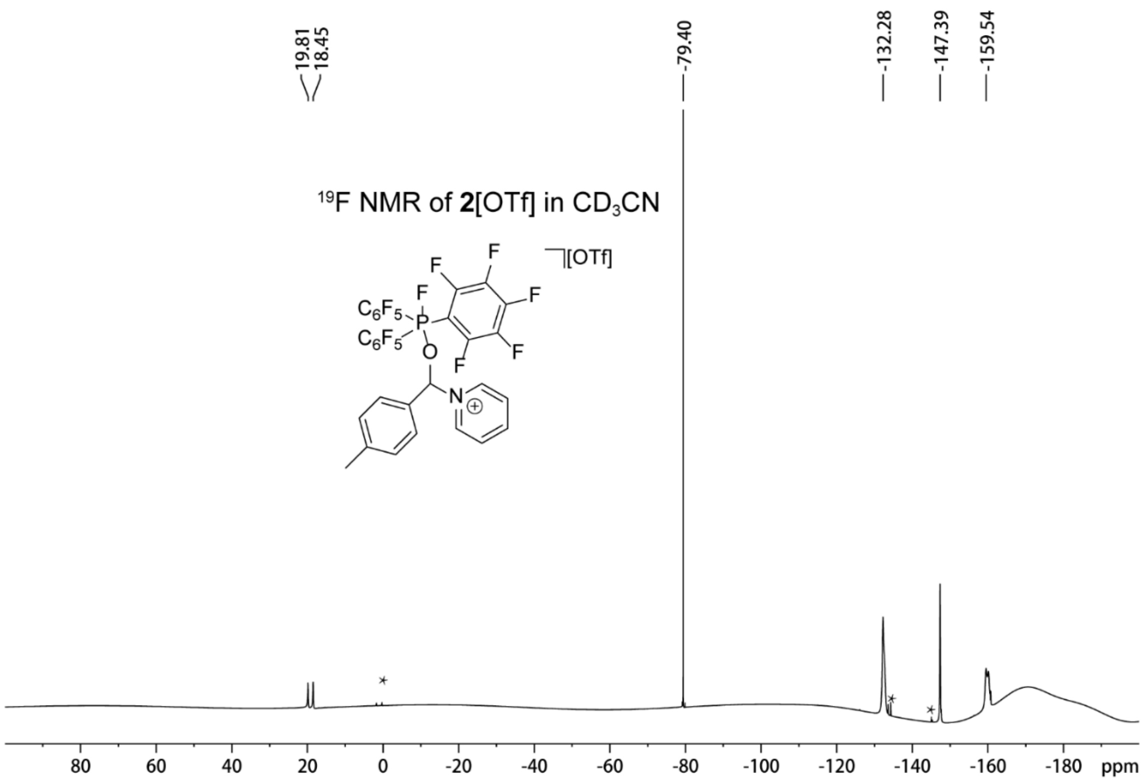

Figure **S22**. ^{19}F NMR spectrum of **2**[OTf] (CD_3CN, 300 K); Asterisks indicate minor amounts of unidentified side products.

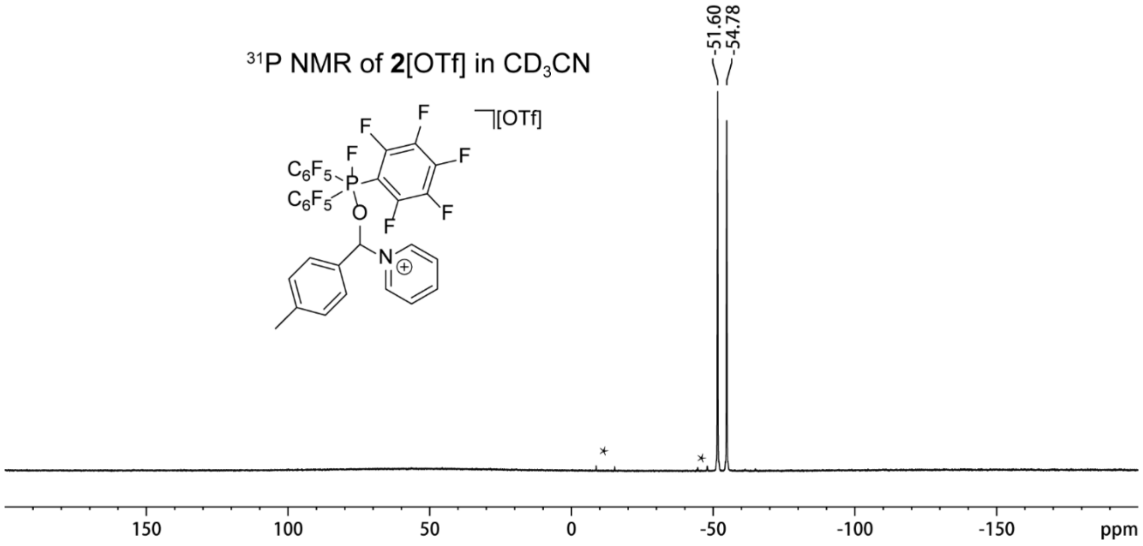

Figure **S23**. ^{31}P{H} NMR spectrum of **2**[OTf] (CD_3CN, 300 K); Asterisks indicate minor amounts of unidentified side products .

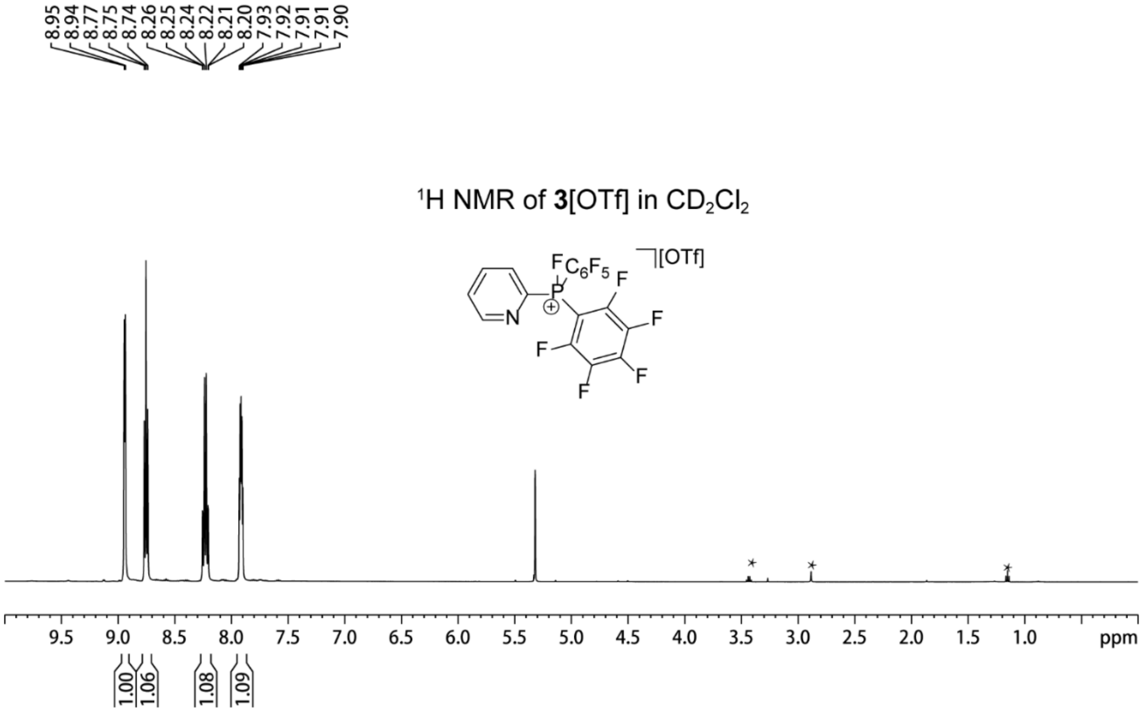

Figure **S24**. ^{1}H NMR spectrum of **3**[OTf] (CD_2Cl_2, 300 K); Asterisks indicate minor amounts of solvent residue.

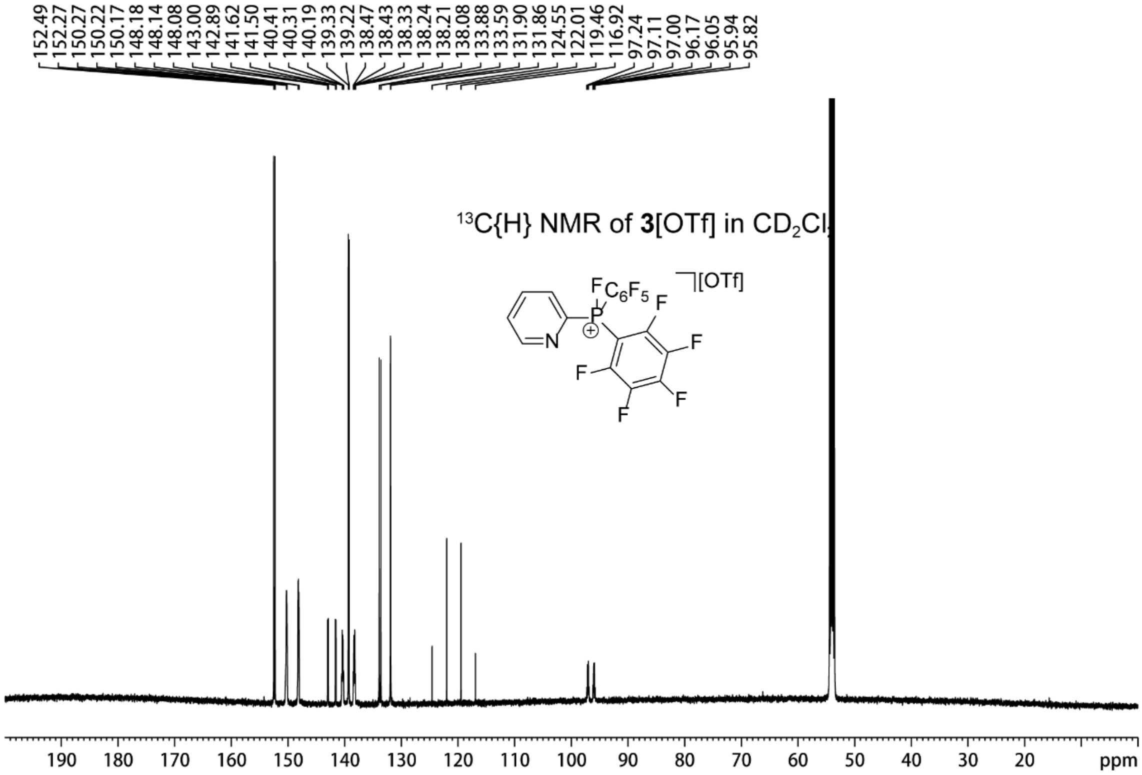

Figure **S25**. $^{13}C\{H\}$ NMR spectrum of **3**[OTf] (CD_2Cl_2, 300 K).

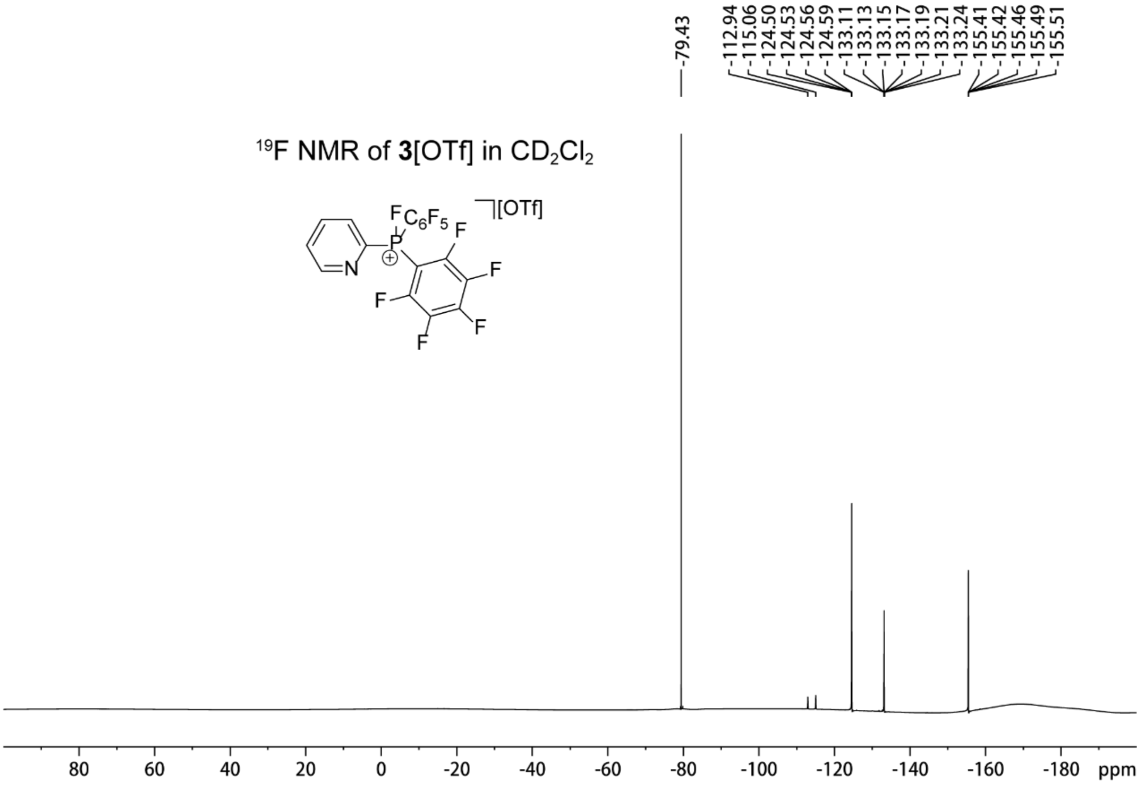

Figure **S26**. ^{19}F NMR spectrum of **3**[OTf] (CD_2Cl_2, 300 K).

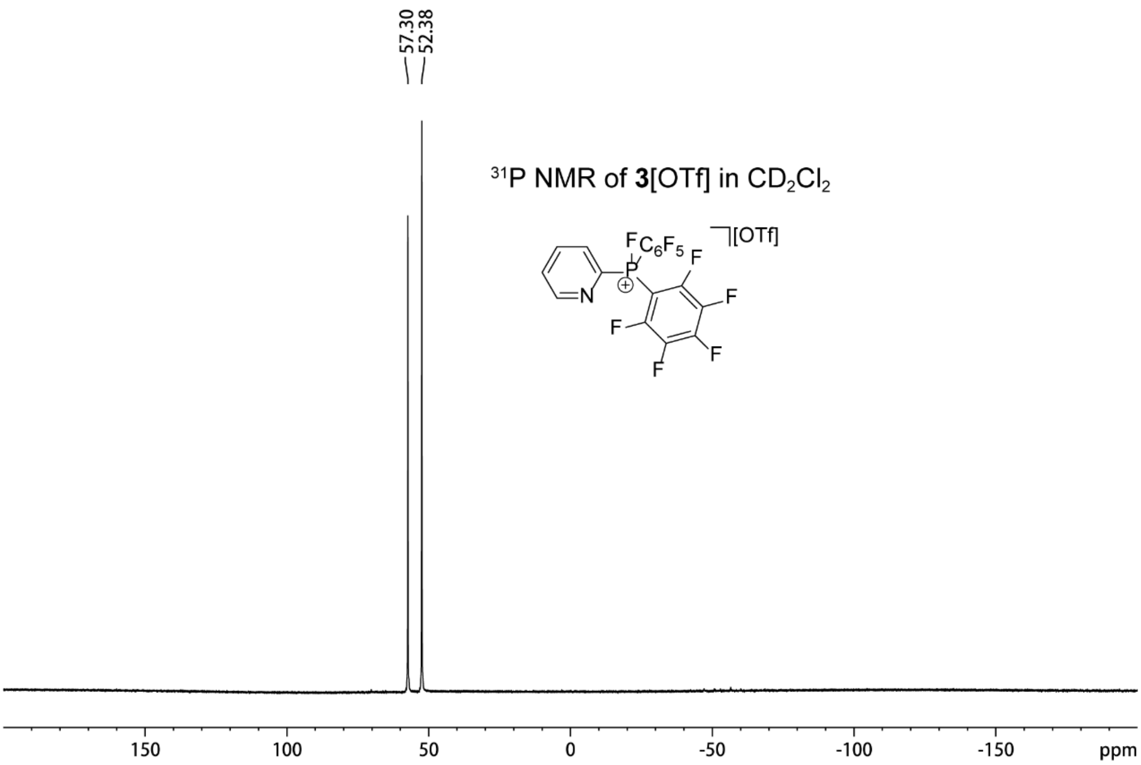

Figure **S27**. ^{31}P{H} NMR spectrum of **3**[OTf] (CD_2Cl_2, 300 K).

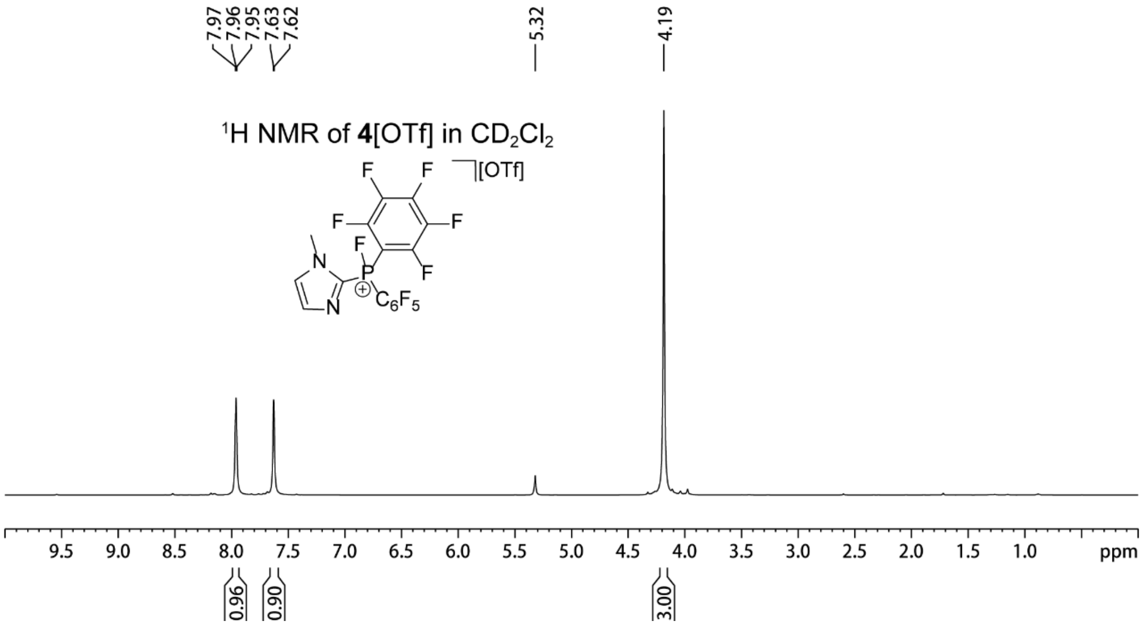

Figure **S28**. ^{1}H NMR spectrum of **4**[OTf] (CD_2Cl_2, 300 K).

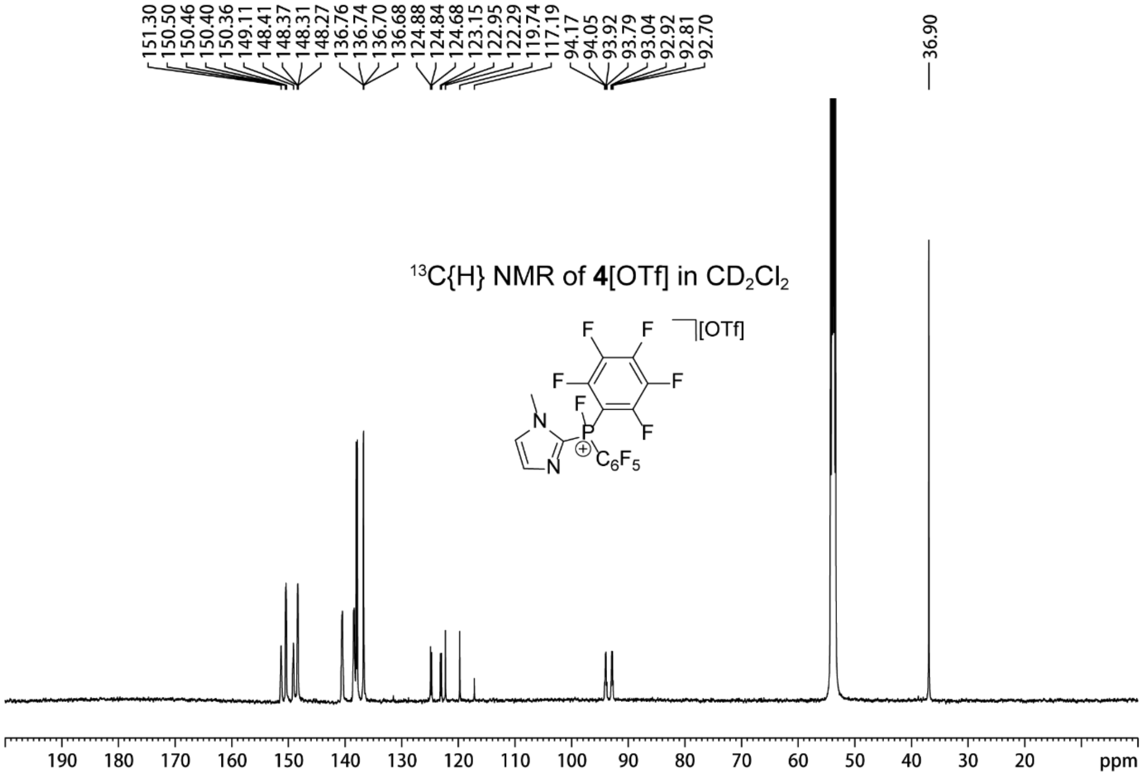

Figure **S29**. ^{13}C{H} NMR spectrum of **4**[OTf] (CD_2Cl_2, 300 K).

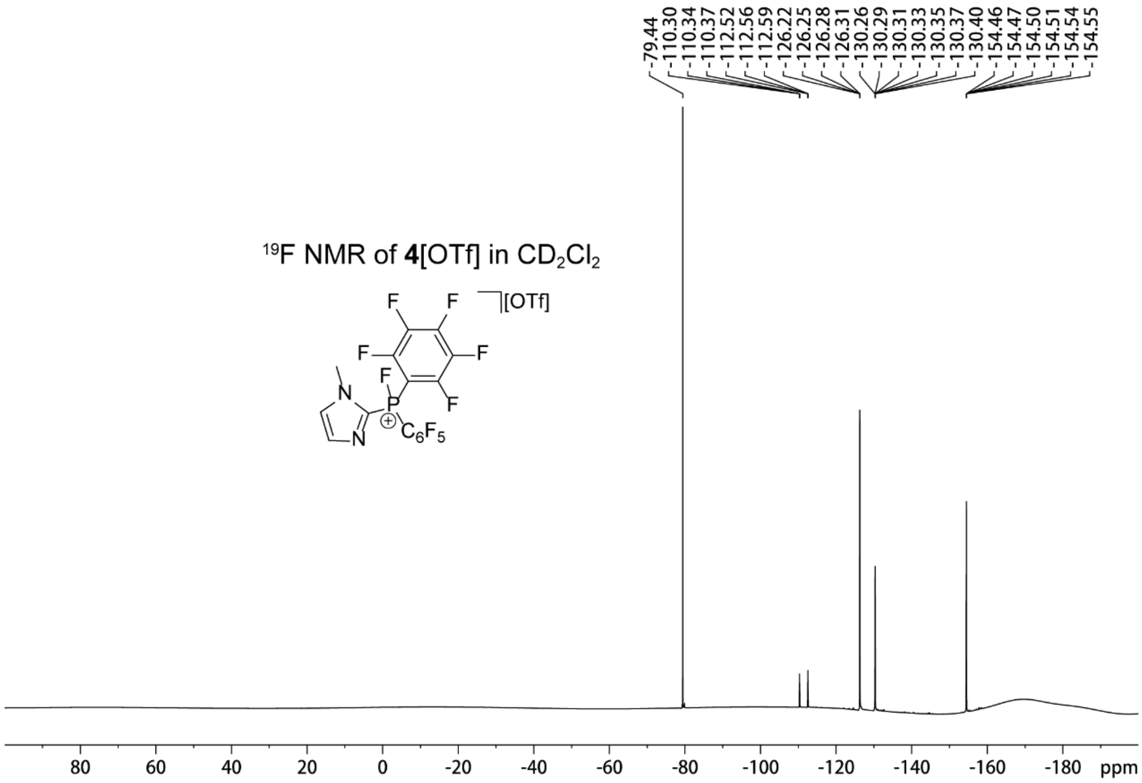

Figure **S30**. ^{19}F NMR spectrum of **4**[OTf] (CD_2Cl_2, 300 K).

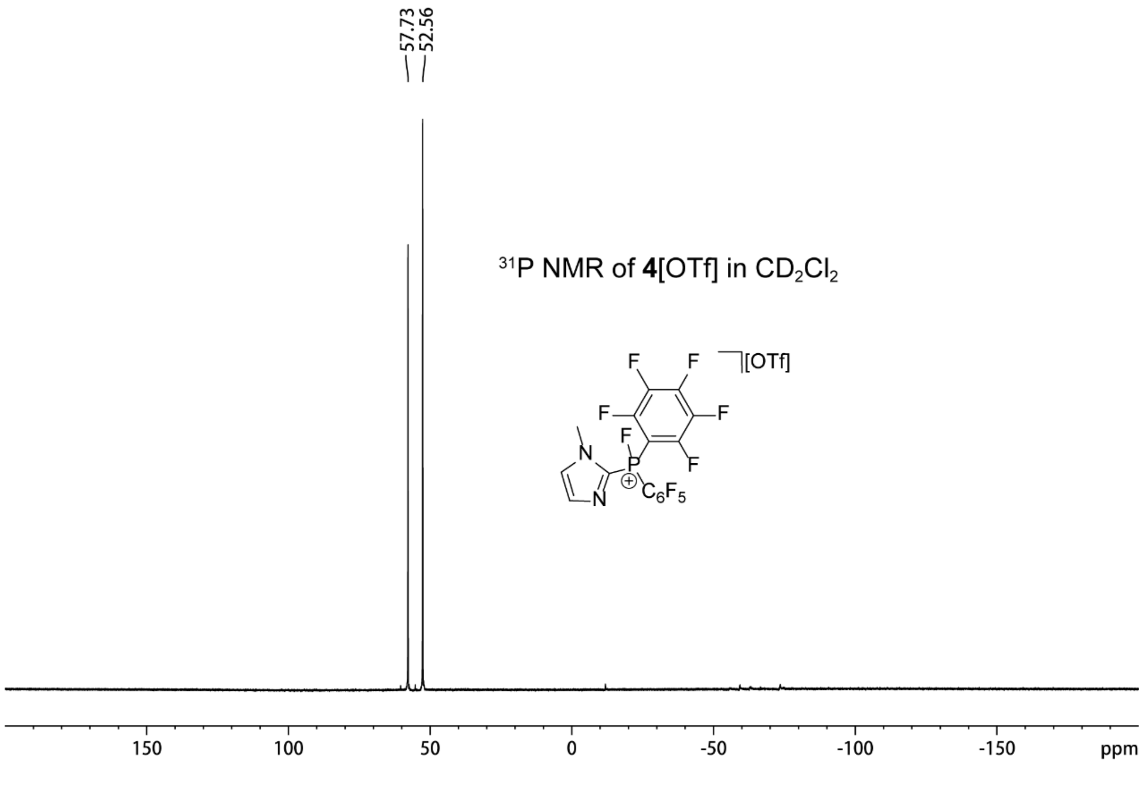

Figure **S31**. ^{31}P{H} NMR spectrum of **4**[OTf] (CD_2Cl_2, 300 K).

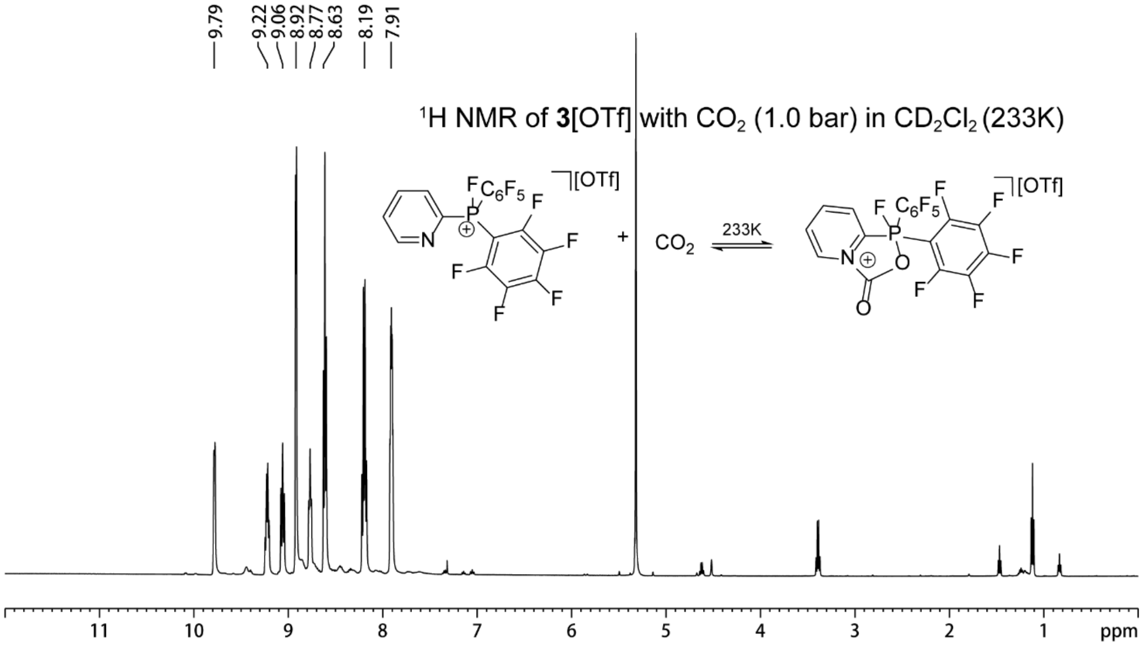

Figure **S32.** *In situ* ^{1}H NMR spectrum of **3**[OTf] under pressurized CO_2 (1.0 bar, CD_2Cl_2, 233 K); stepwise decrease the temperature from 300 K to 233 K.

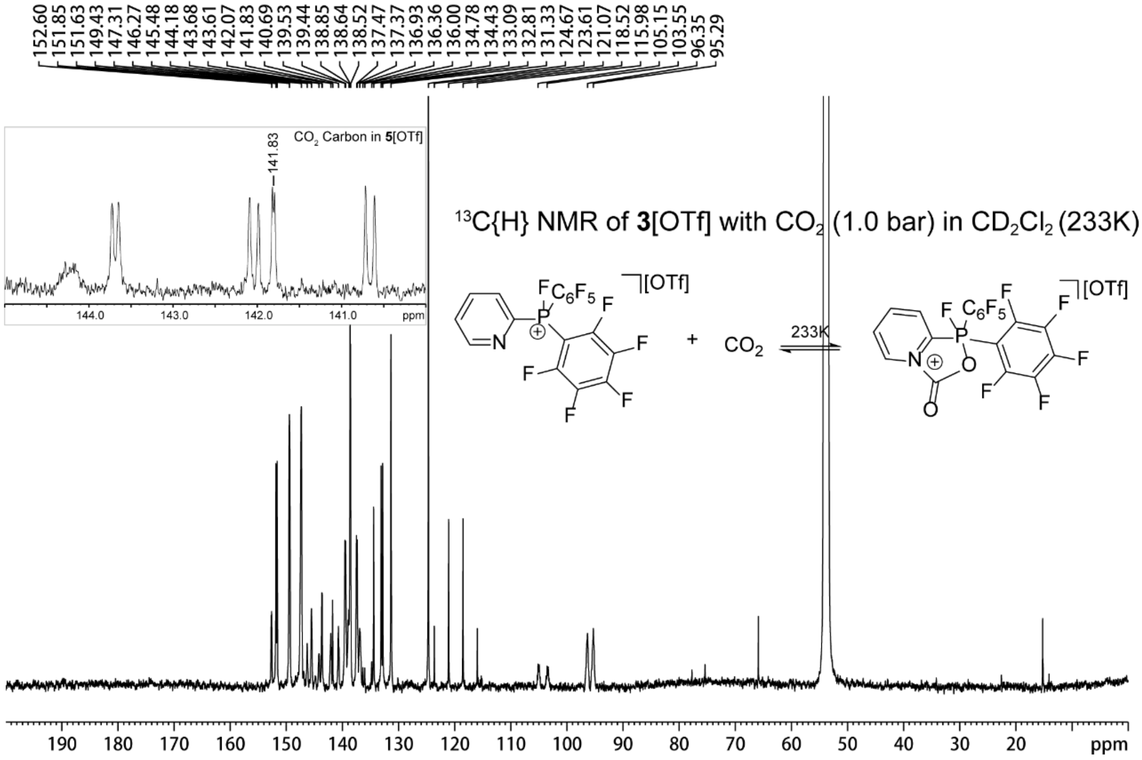

Figure **S33.** *In situ* ^{13}C{H} NMR spectrum of **3**[OTf] under pressurized CO_2 (1.0 bar, CD_2Cl_2, 233 K); stepwise decrease the temperature from 300 K to 233 K.

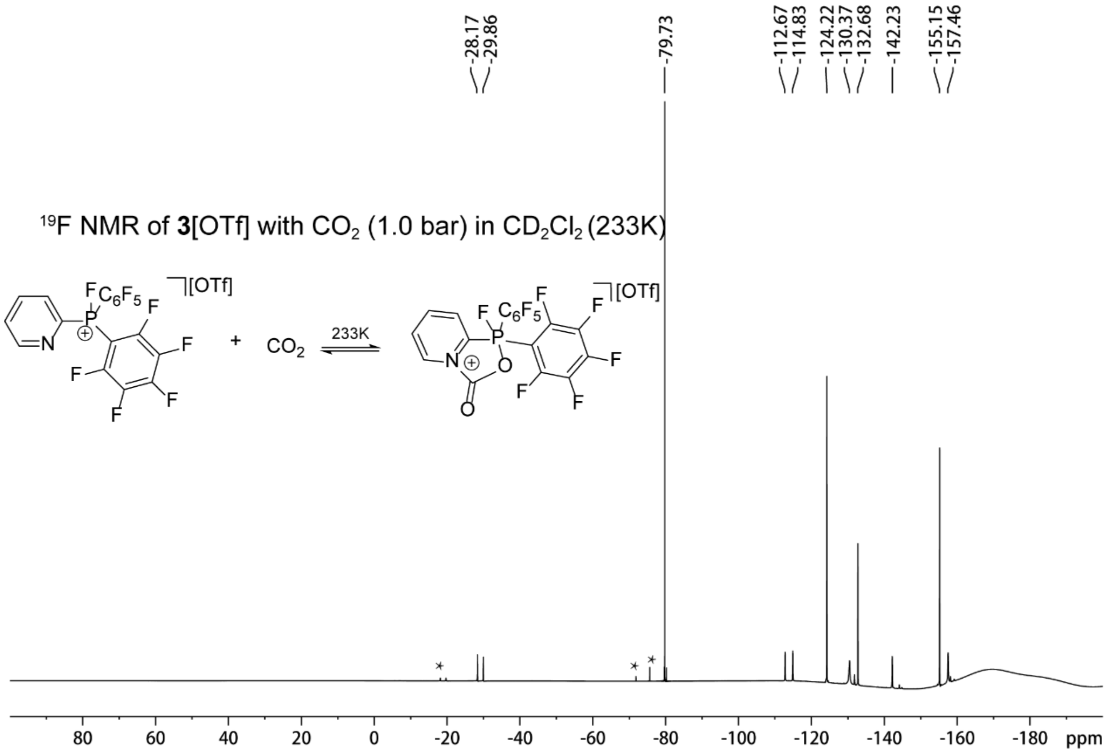

Figure **S34.** *In situ* ^{19}F NMR spectrum of **3**[OTf] under pressurized CO_2 (1.0 bar, CD_2Cl_2, 233 K); Stepwise decrease the temperature from 300 K to 233 K; Asterisks indicate small amounts of unidentified side products.

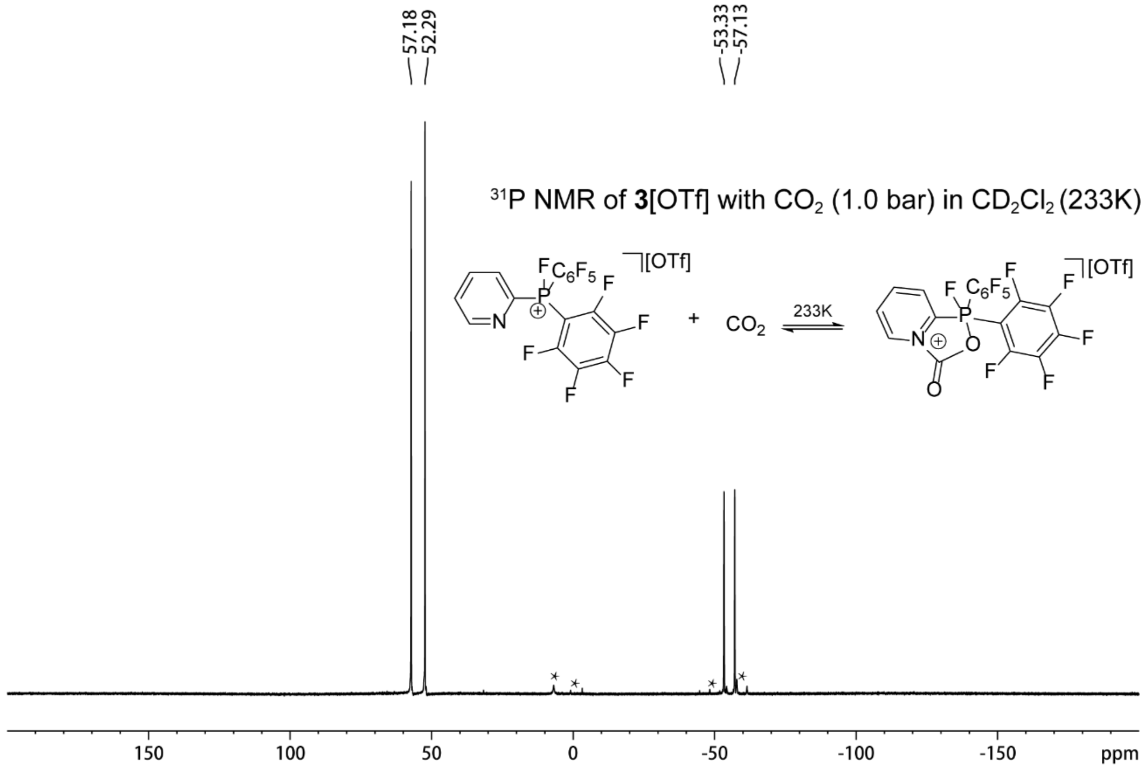

Figure **S35.** *In situ* ^{31}P{H} NMR spectrum of **3**[OTf] under pressurized CO_2 (1.0 bar, CD_2Cl_2, 233 K); stepwise decrease the temperature from 300 K to 233 K; Asterisks indicate small amounts of unidentified side products.

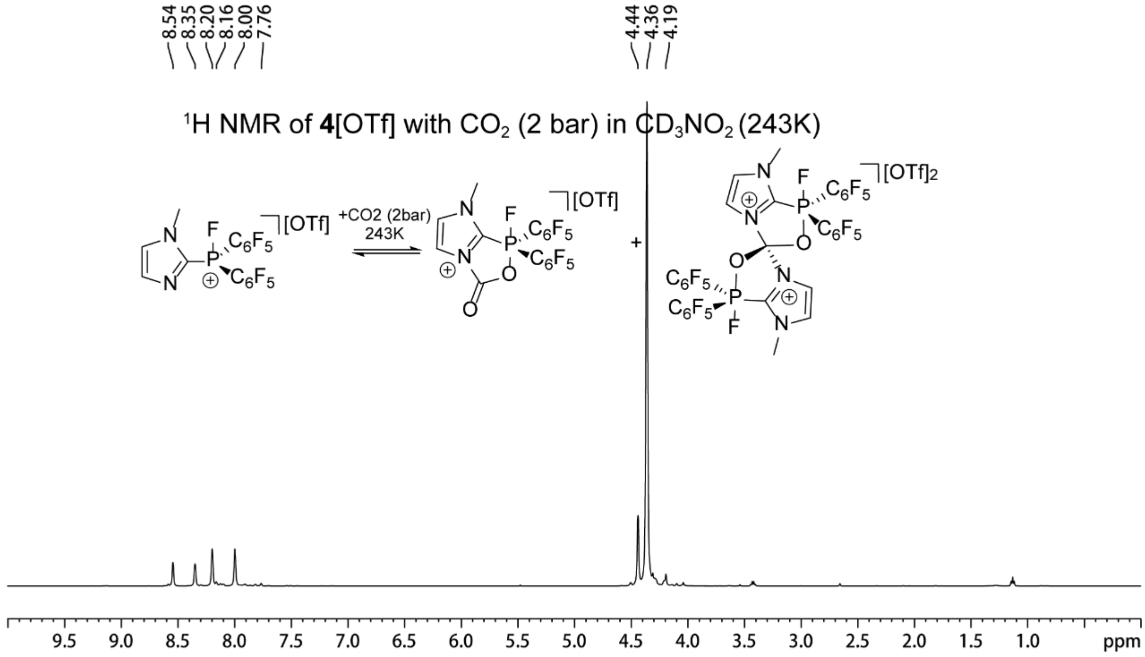

Figure **S36.** *In situ* ^1H NMR spectrum of **4**[OTf] under pressurized CO_2 (2 bar, CD_3NO_2, 243 K); Stepwise decrease the temperature from 300 K to 243 K.

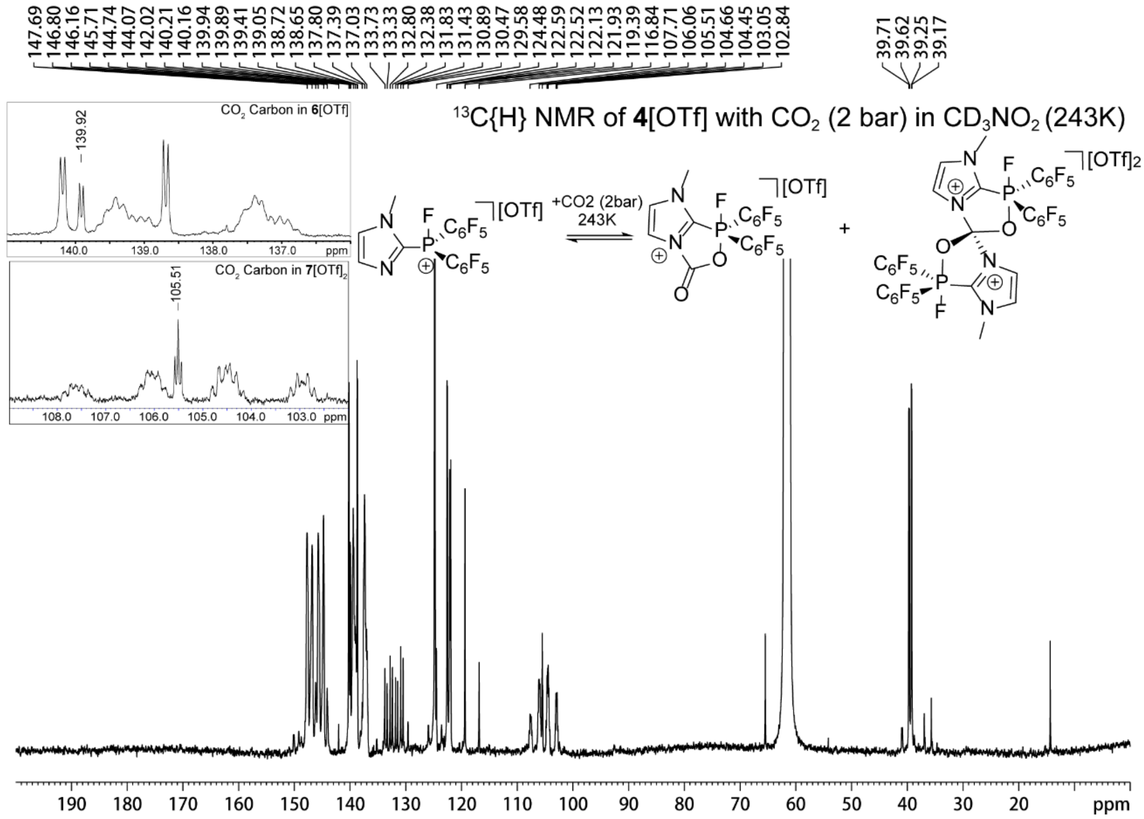

Figure **S37.** *In situ* $^{13}C\{H\}$ NMR spectrum of **4**[OTf] under pressurized CO_2 (2 bar, CD_3NO_2, 243 K); Stepwise decrease the temperature from 300 K to 243 K.

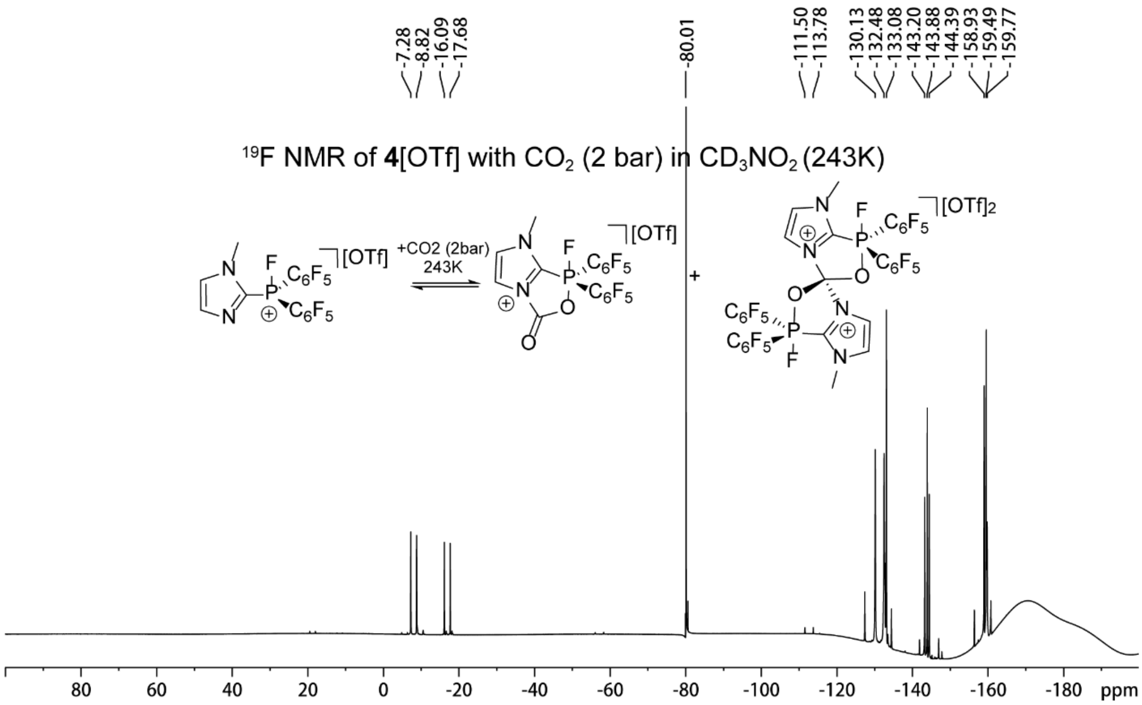

Figure **S38.** *In situ* ^{19}F NMR spectrum of **4**[OTf] under pressurized CO_2 (2 bar, CD_3NO_2, 243 K); Stepwise decrease the temperature from 300 K to 243 K; Asterisks indicate small amounts of unidentified side products.

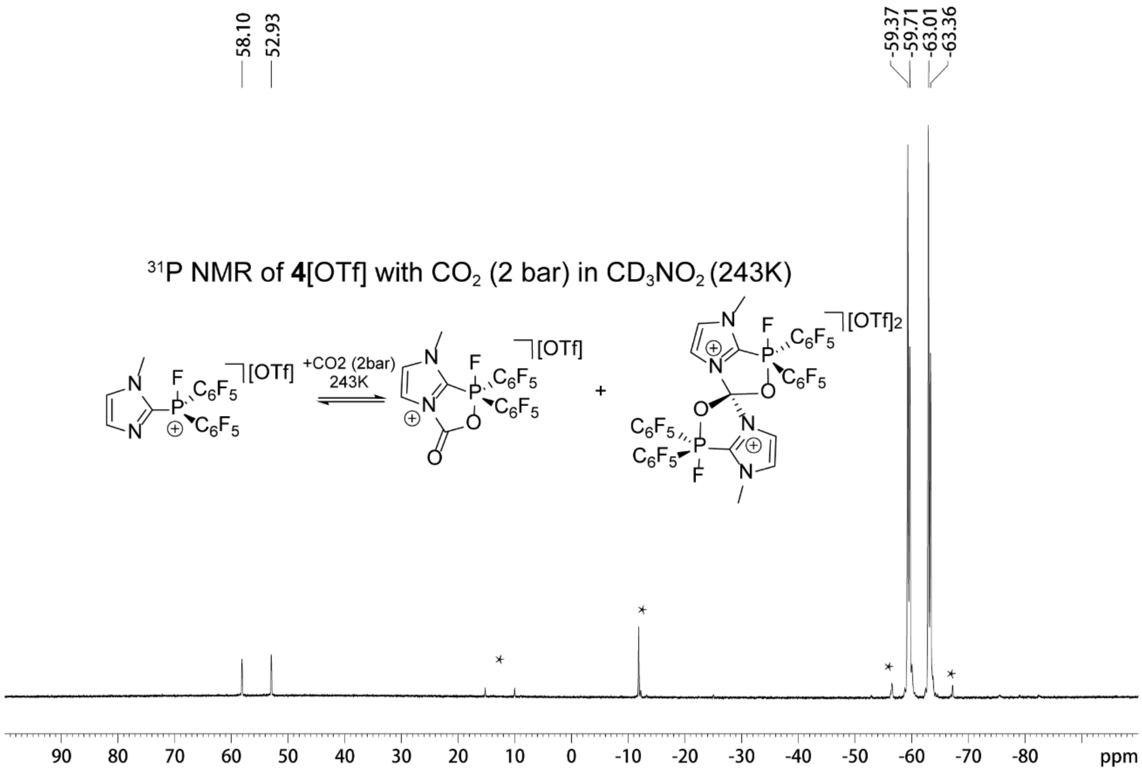

Figure **S39.** *In situ* ^{31}P NMR spectrum of **4**[OTf] under pressurized CO_2 (2 bar, CD_3NO_2, 243 K); Stepwise decrease the temperature from 300 K to 243 K; Asterisks indicate small amounts of unidentified side products.

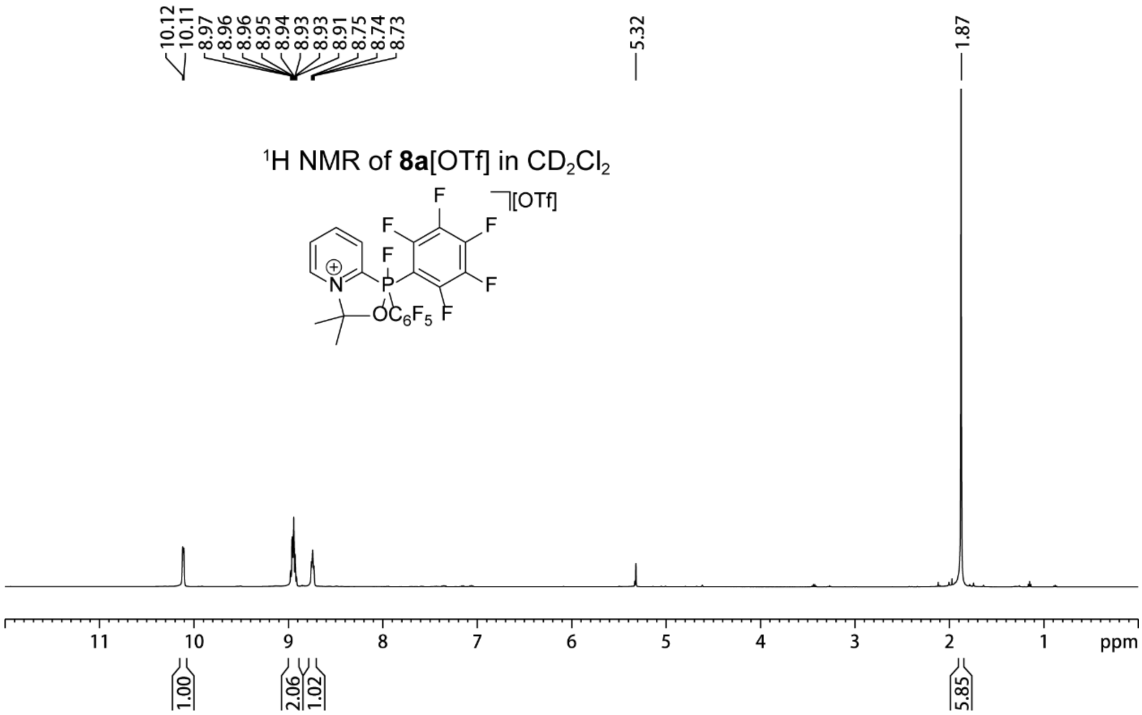

Figure **S40**. ^{1}H NMR spectrum of **8a**[OTf] (CD_2Cl_2, 300 K).

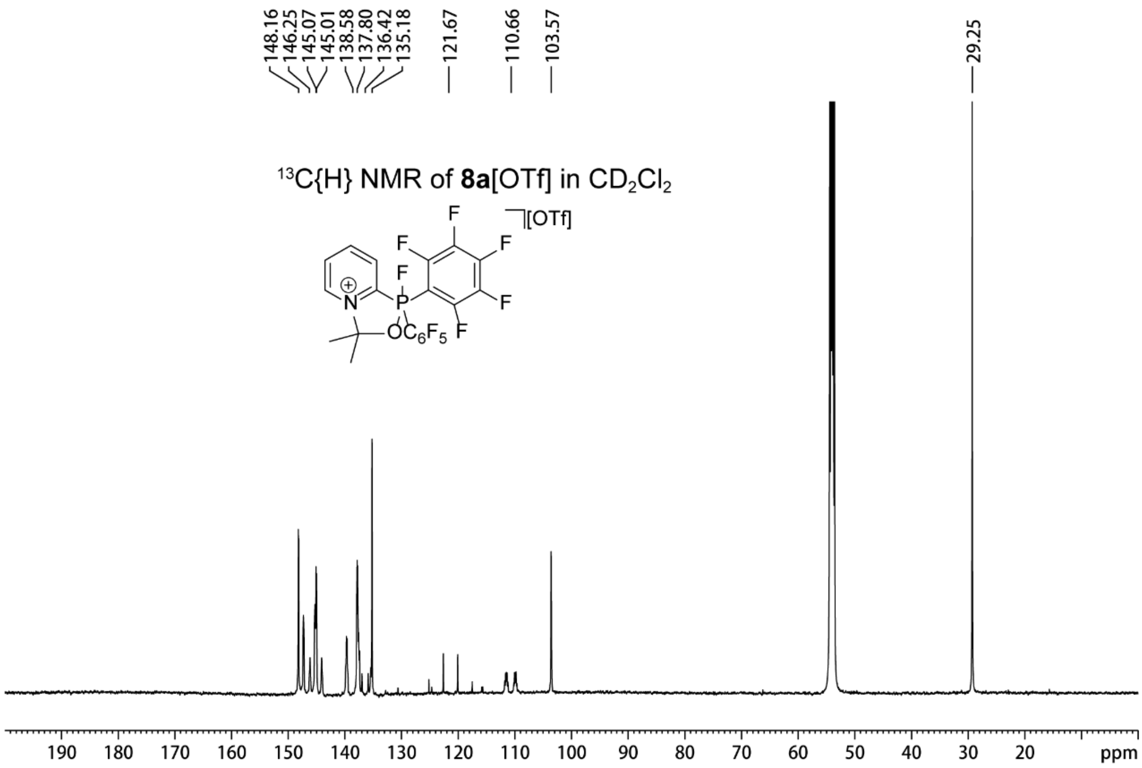

Figure **S41**. ^{13}C{H} NMR spectrum of **8a**[OTf] (CD_2Cl_2, 300 K).

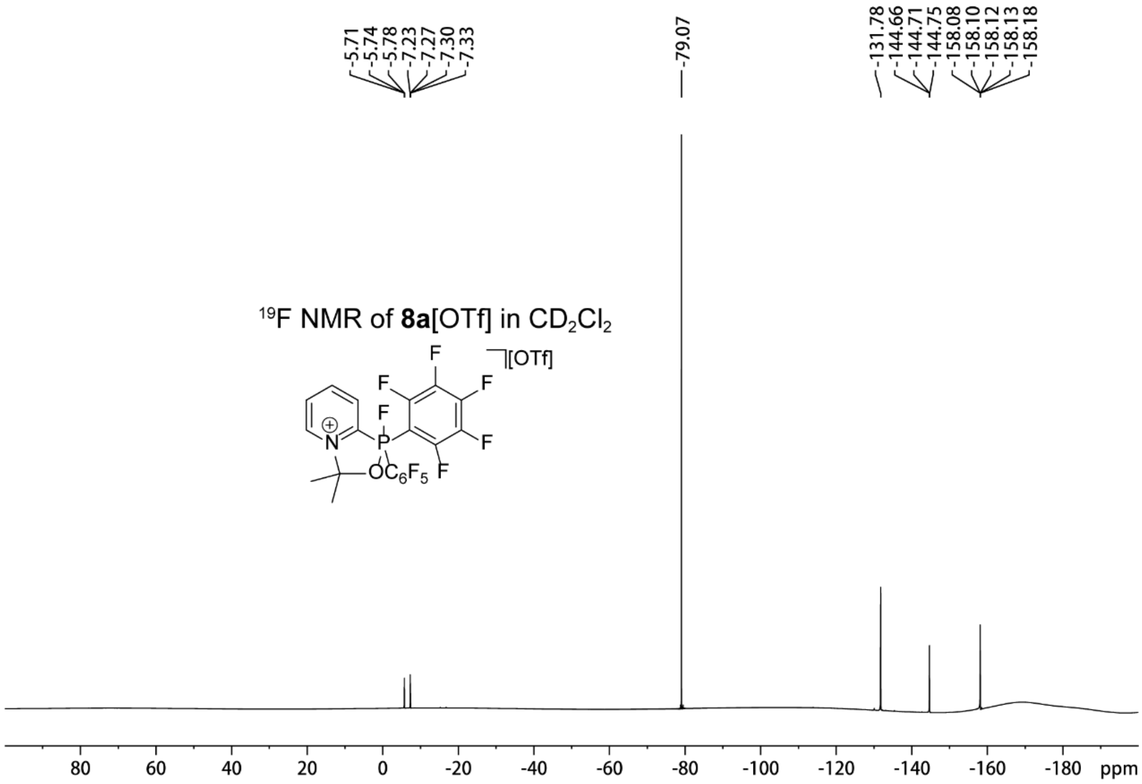

Figure **S42**. ^{19}F NMR spectrum of **8a**[OTf] (CD_2Cl_2, 300 K).

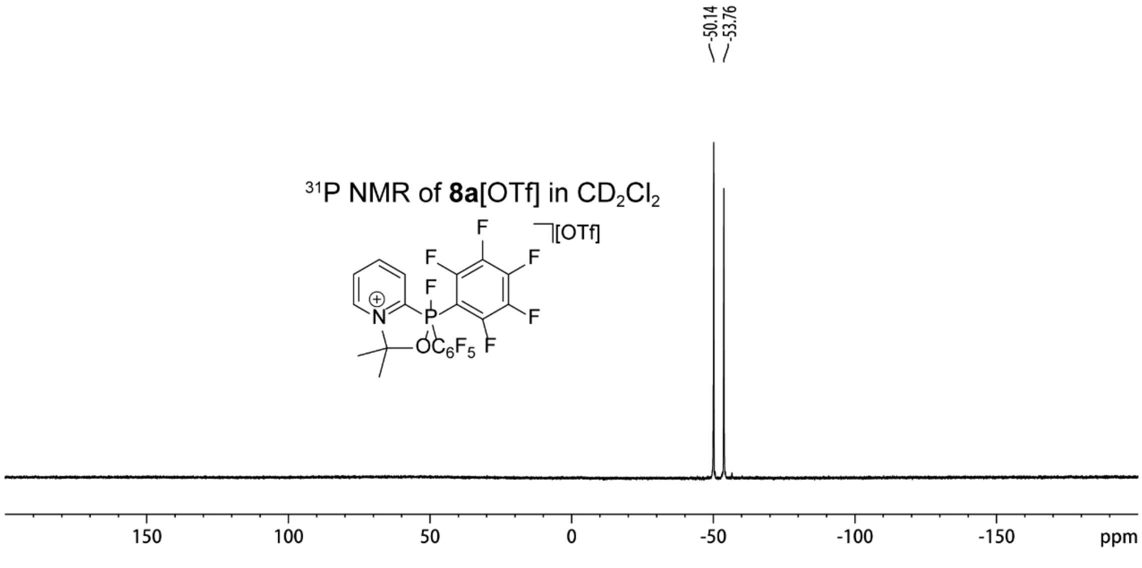

Figure **S43**. ^{31}P{H} NMR spectrum of **8a**[OTf] (CD_2Cl_2, 300 K).

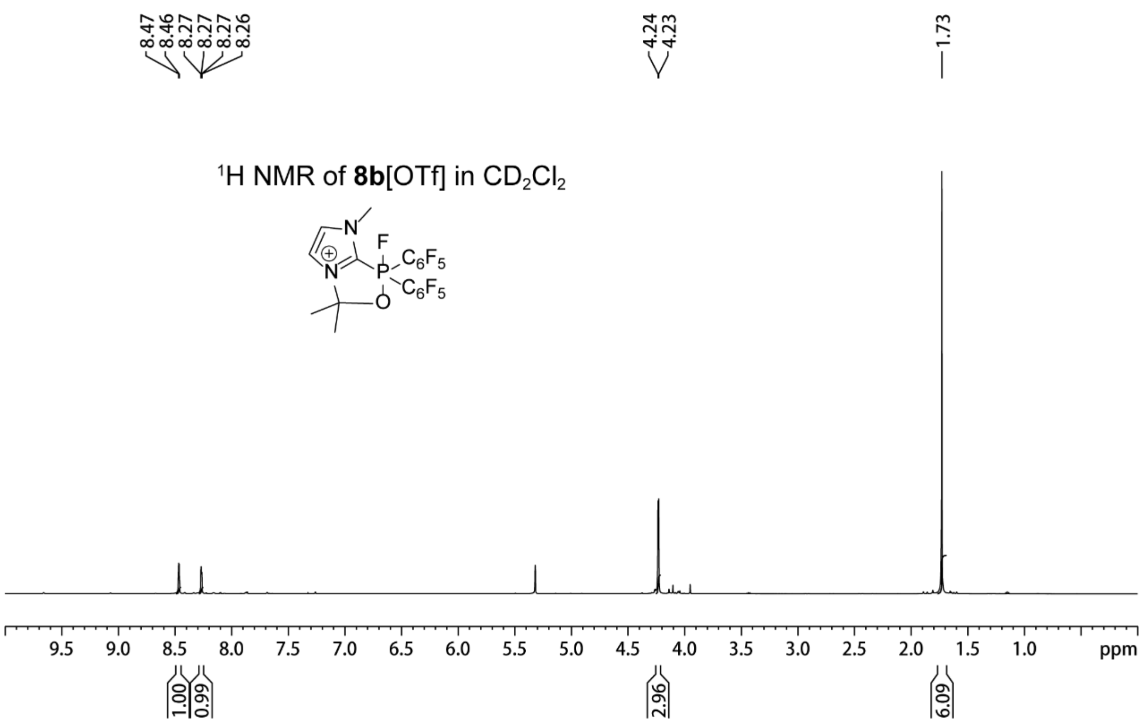

Figure **S44**. ^{1}H NMR spectrum of **8a**[OTf] (CD_2Cl_2, 300 K).

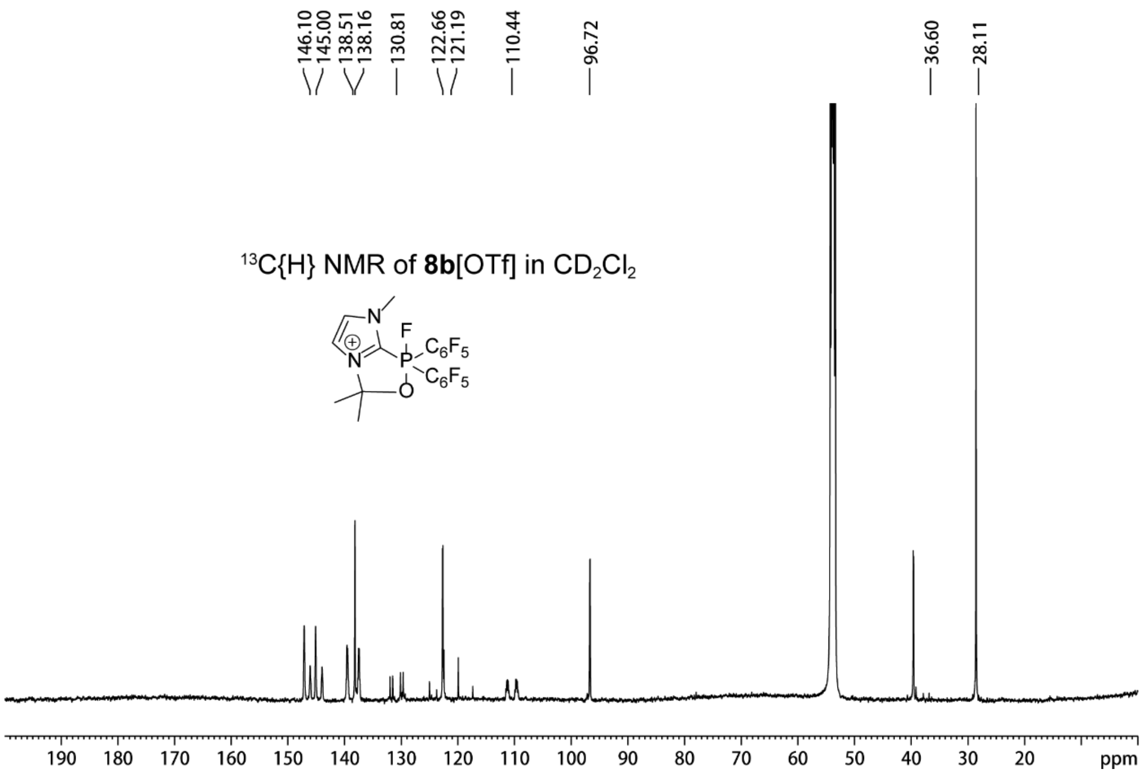

Figure **S45**. $^{13}C\{H\}$ NMR spectrum of **8a**[OTf] (CD_2Cl_2, 300 K).

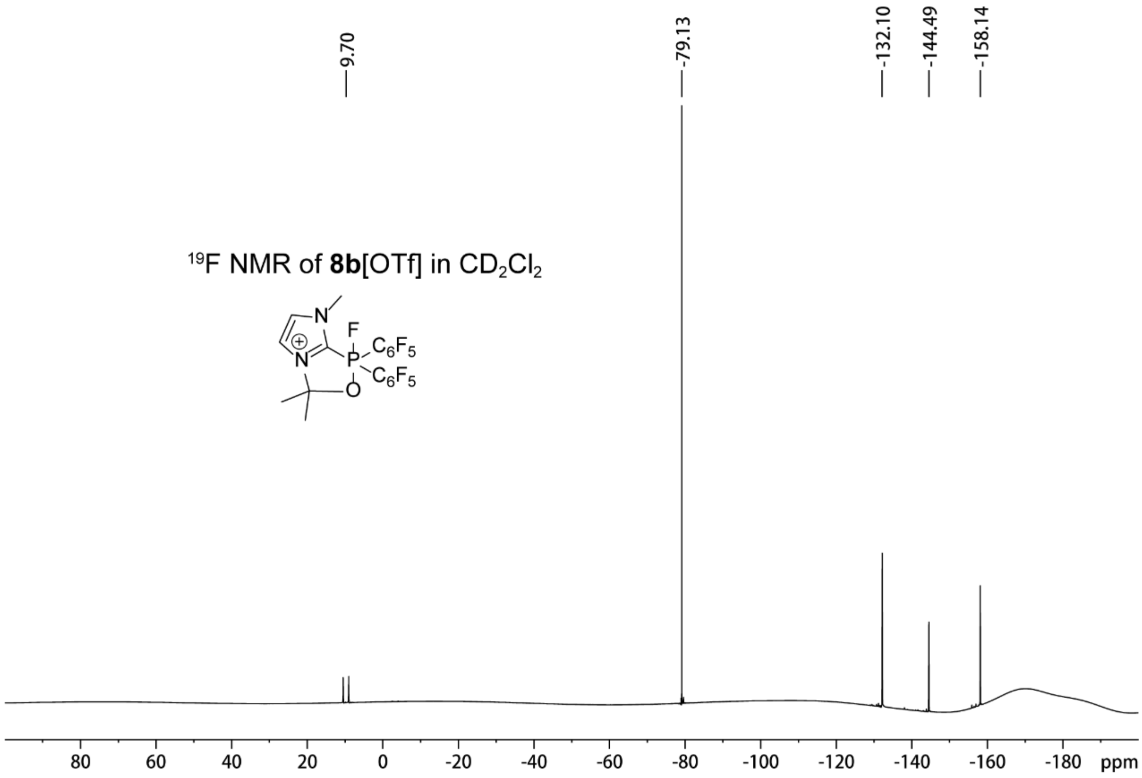

Figure **S46**. ^{19}F NMR spectrum of **8a**[OTf] (CD_2Cl_2, 300 K).

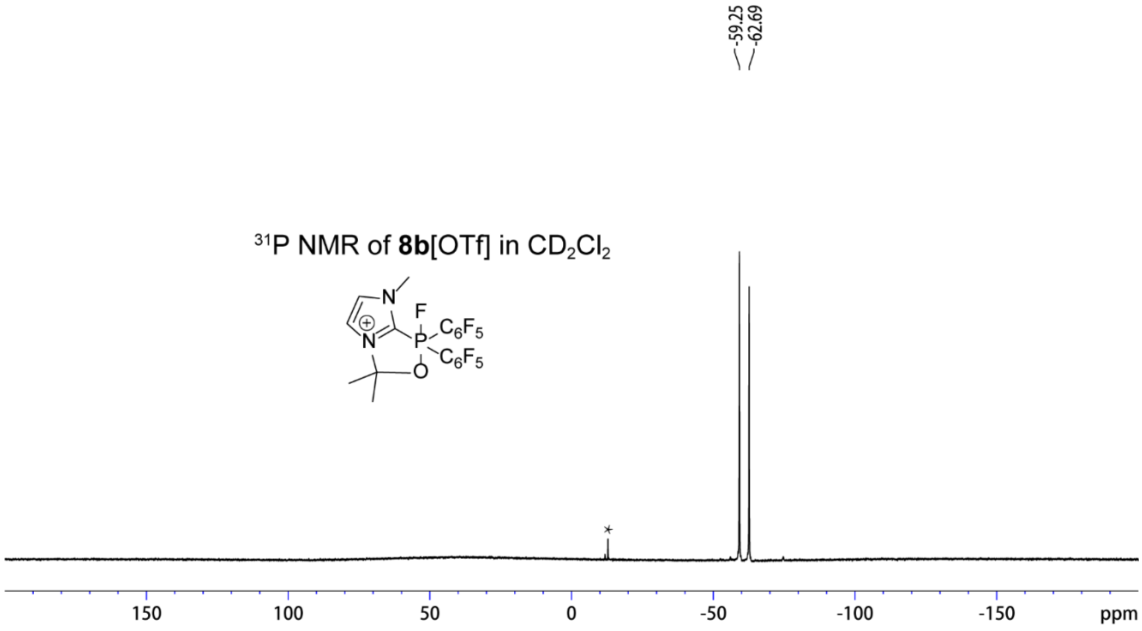

Figure **S47**. ^{31}P{H} NMR spectrum of **8a**[OTf] (CD_2Cl_2, 300 K); Asterisks indicate small amounts of unidentified side products.

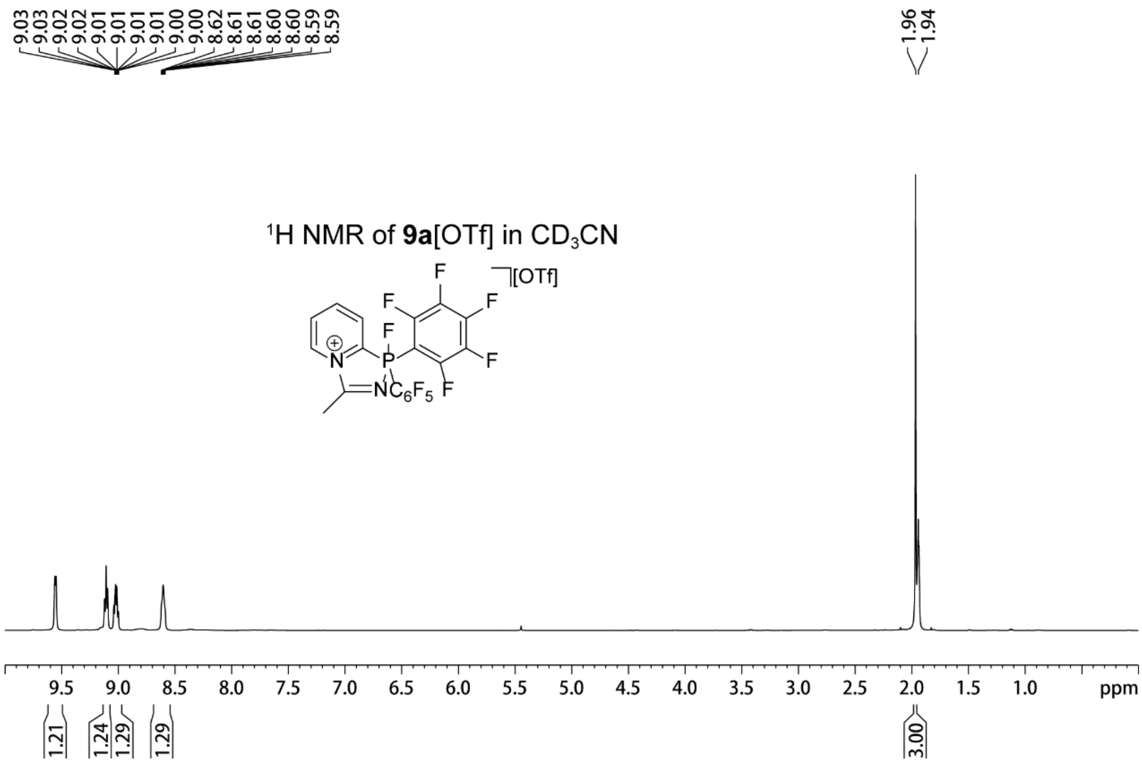

Figure **S48**. ^{1}H NMR spectrum of **9a**[OTf] (CD_3CN, 300 K).

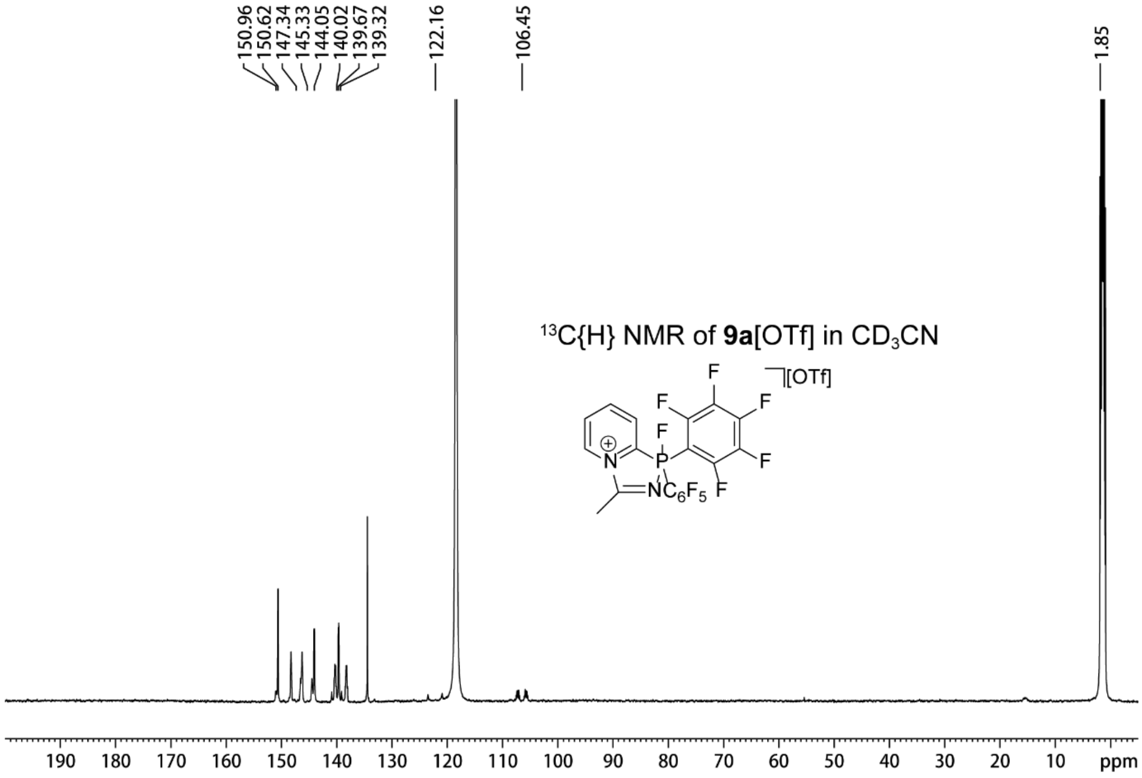

Figure **S49**. $^{13}C\{H\}$ NMR spectrum of **9a**[OTf] (CD_3CN, 300 K).

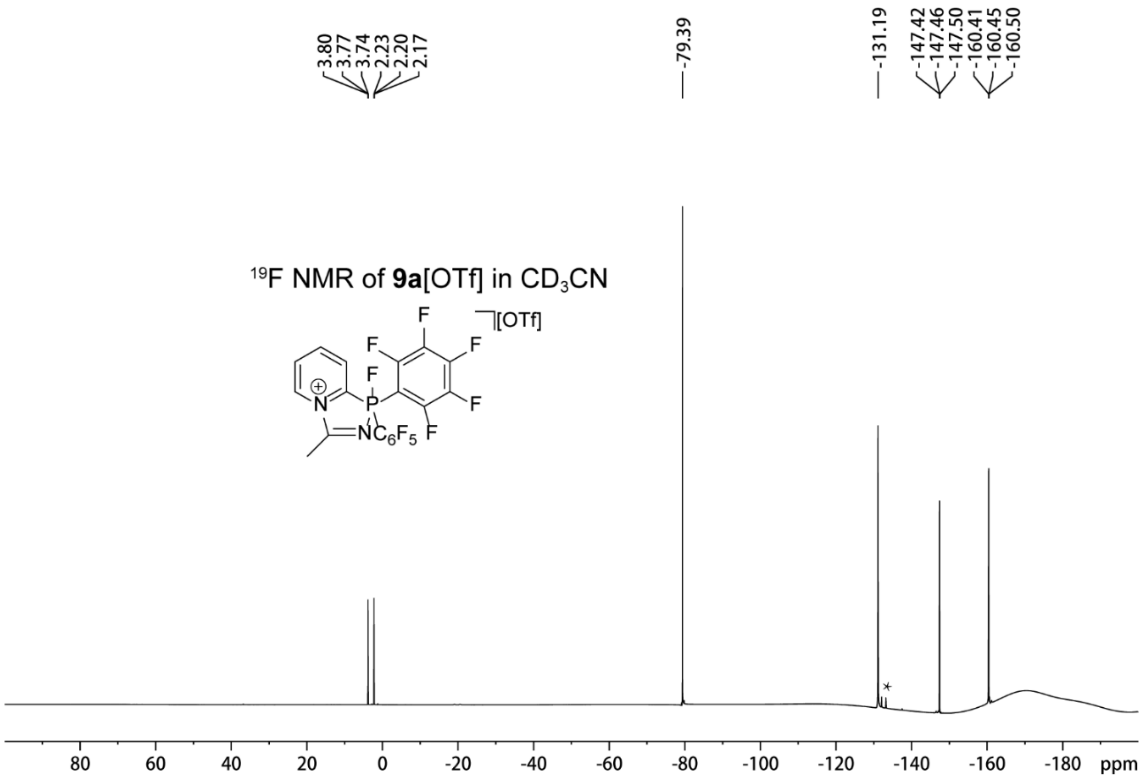

Figure **S50**. ^{19}F NMR spectrum of **9a**[OTf] (CD_3CN, 300 K); Asterisks indicate small amounts of unidentified side products.

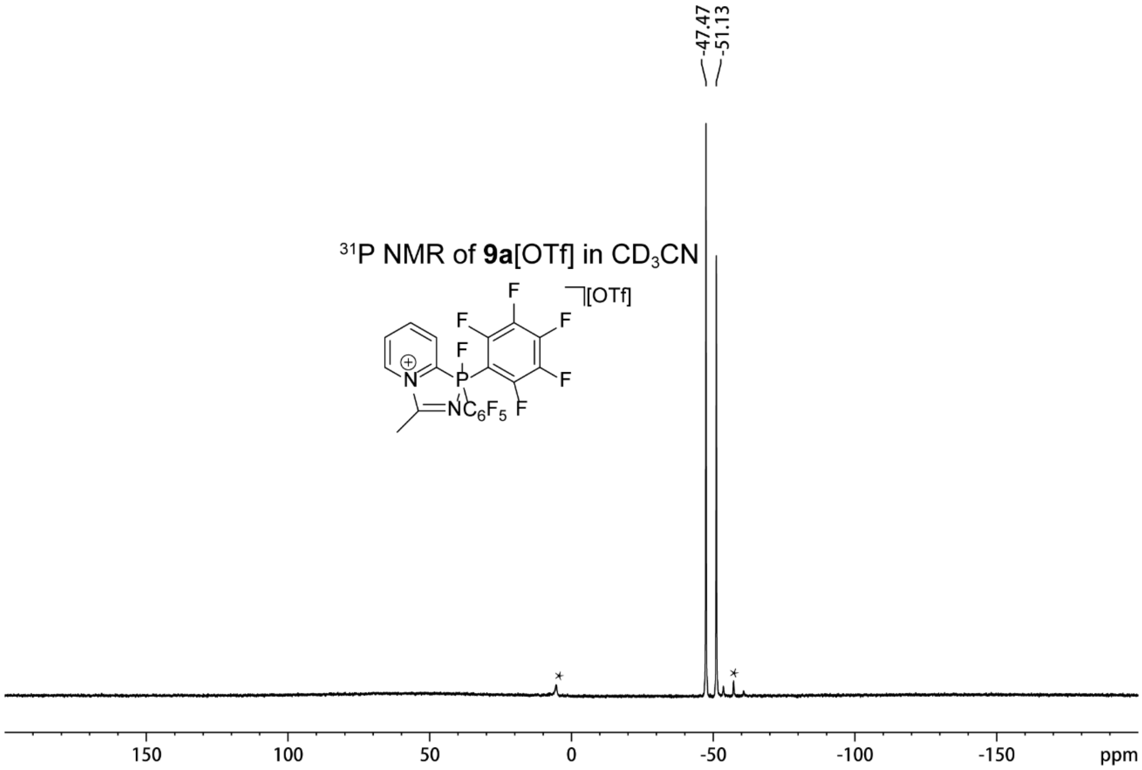

Figure **S51.** $^{31}P\{H\}$ NMR spectrum of **9a**[OTf] (CD_3CN, 300 K); Asterisks indicate small amounts of unidentified side products.

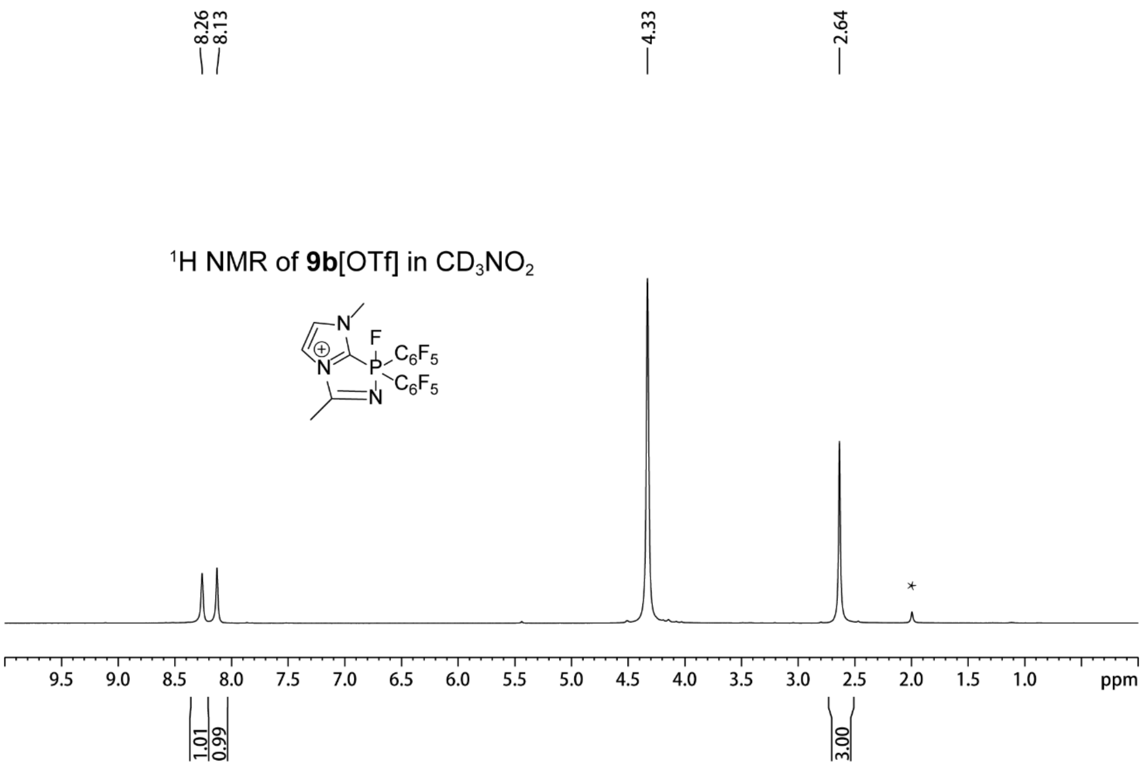

Figure **S52.** ^{1}H NMR spectrum of **9b**[OTf] (CD_3NO_2, 300 K); Asterisks indicate small amounts of CH_3CN.

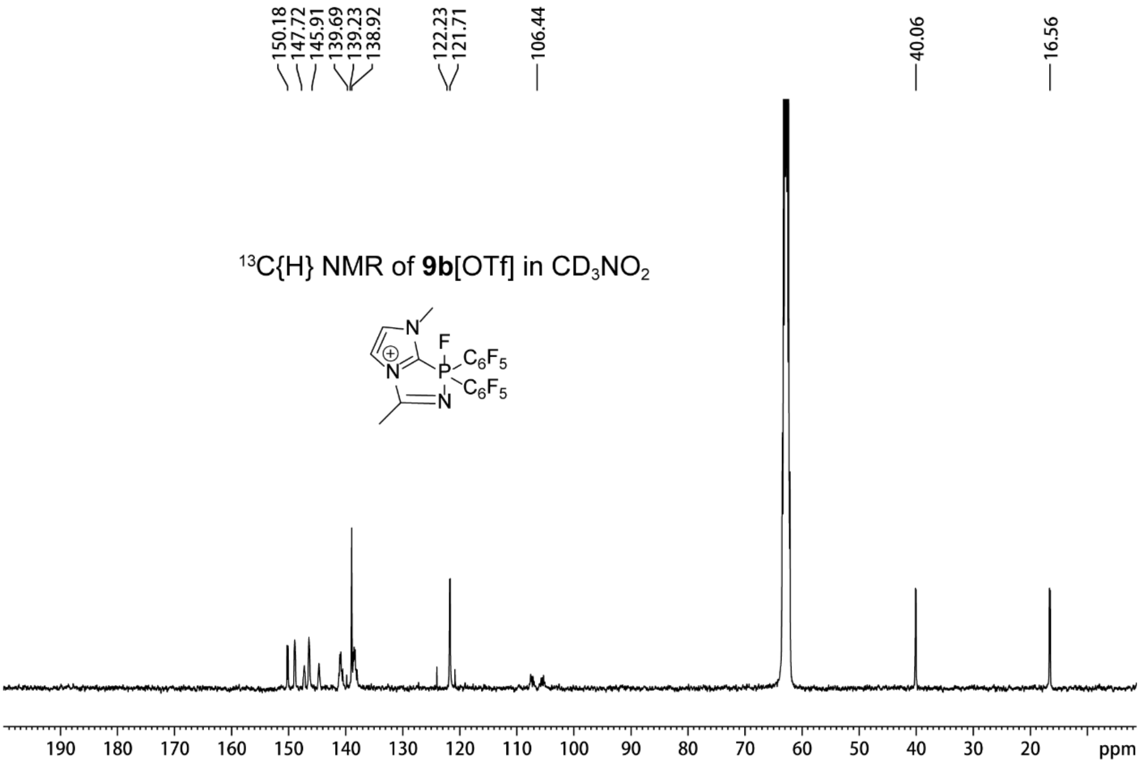

Figure **S53.** ^{13}C{H} NMR spectrum of **9b**[OTf] (CD_3NO_2, 300 K).

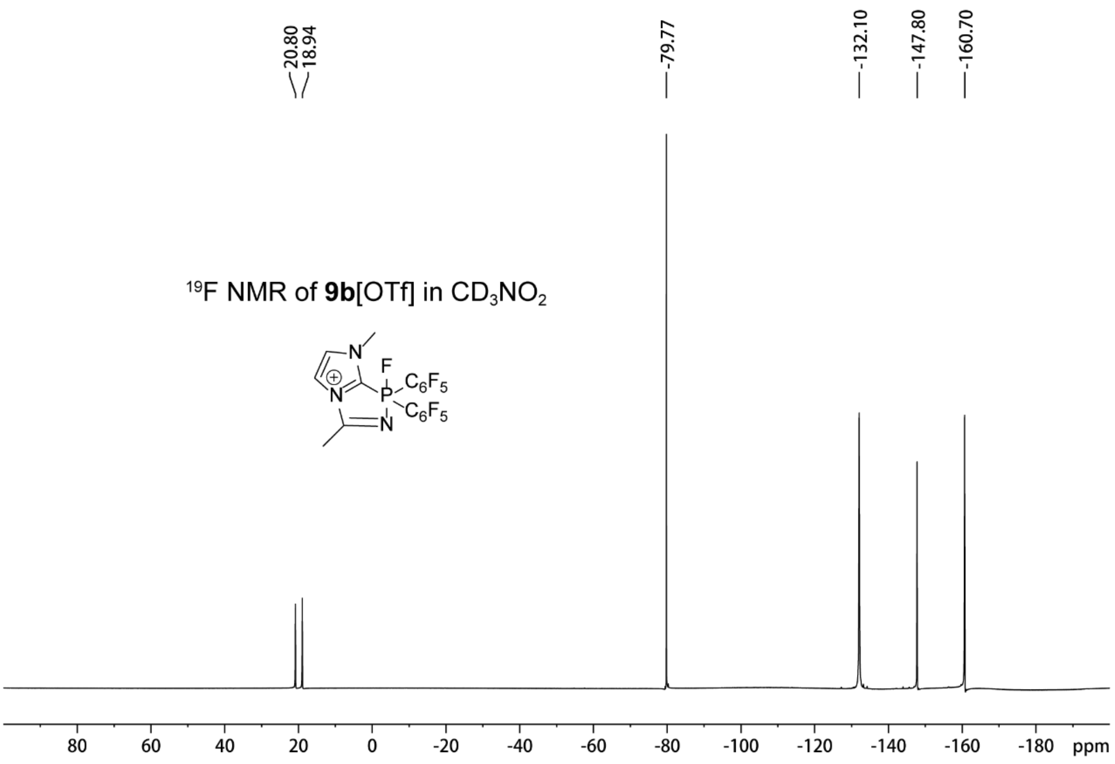

Figure **S54.** ^{19}F NMR spectrum of **9b**[OTf] (CD_3NO_2, 300 K).

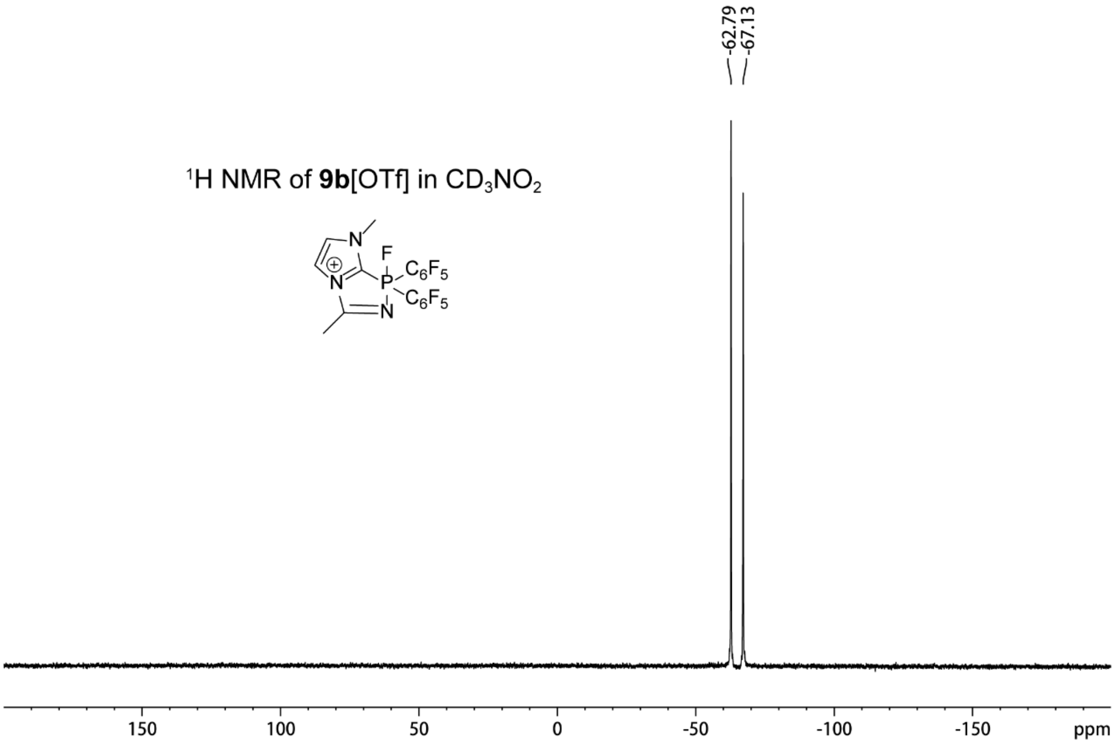

Figure **S55.** $^{31}P\{H\}$ NMR spectrum of **9b**[OTf] (CD_3NO_2, 300 K).

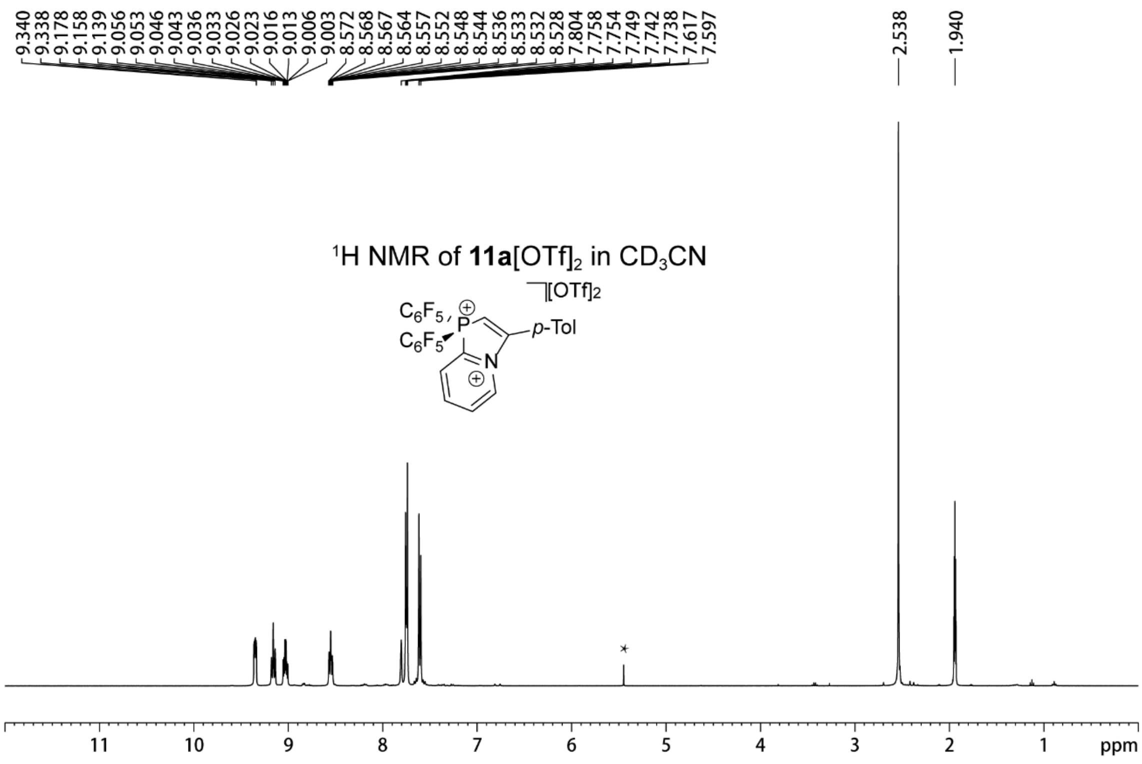

Figure **S56.** ^1H NMR spectrum of **11a**[OTf]$_2$ (CD_3CN, 300 K); Asterisks indicate small amounts of residual solvent.

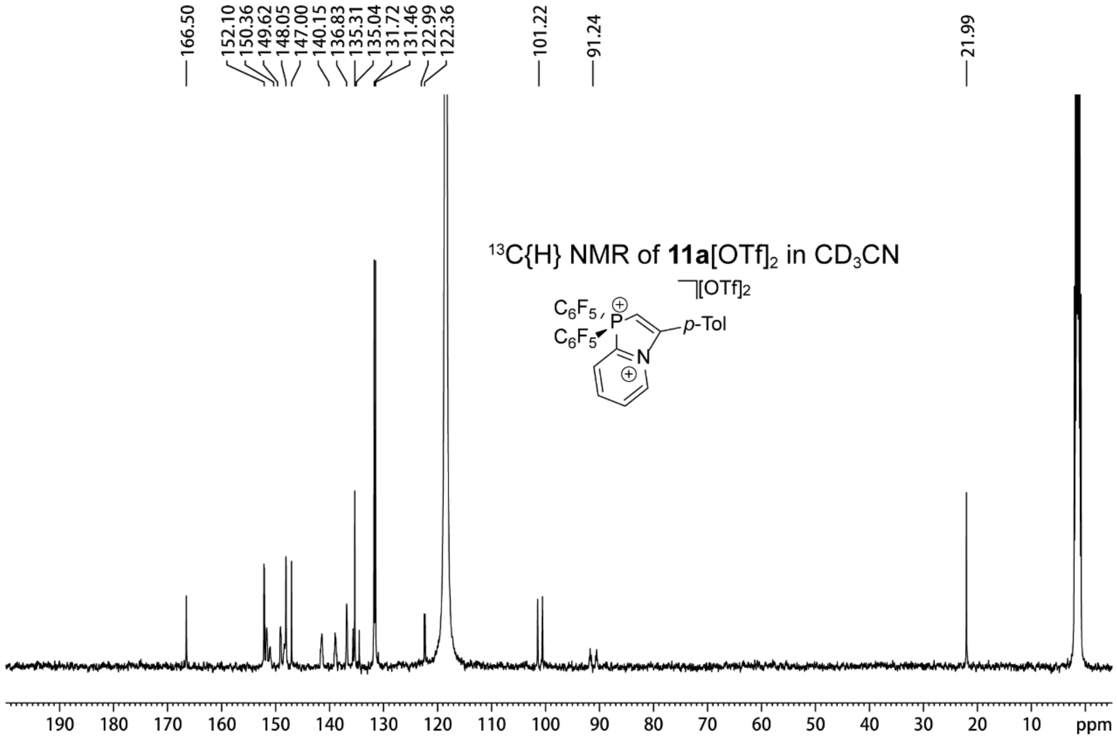

Figure **S57.**$^{13}C\{H\}$ NMR spectrum of **11a**[OTf]$_2$ (CD_3CN, 300 K).

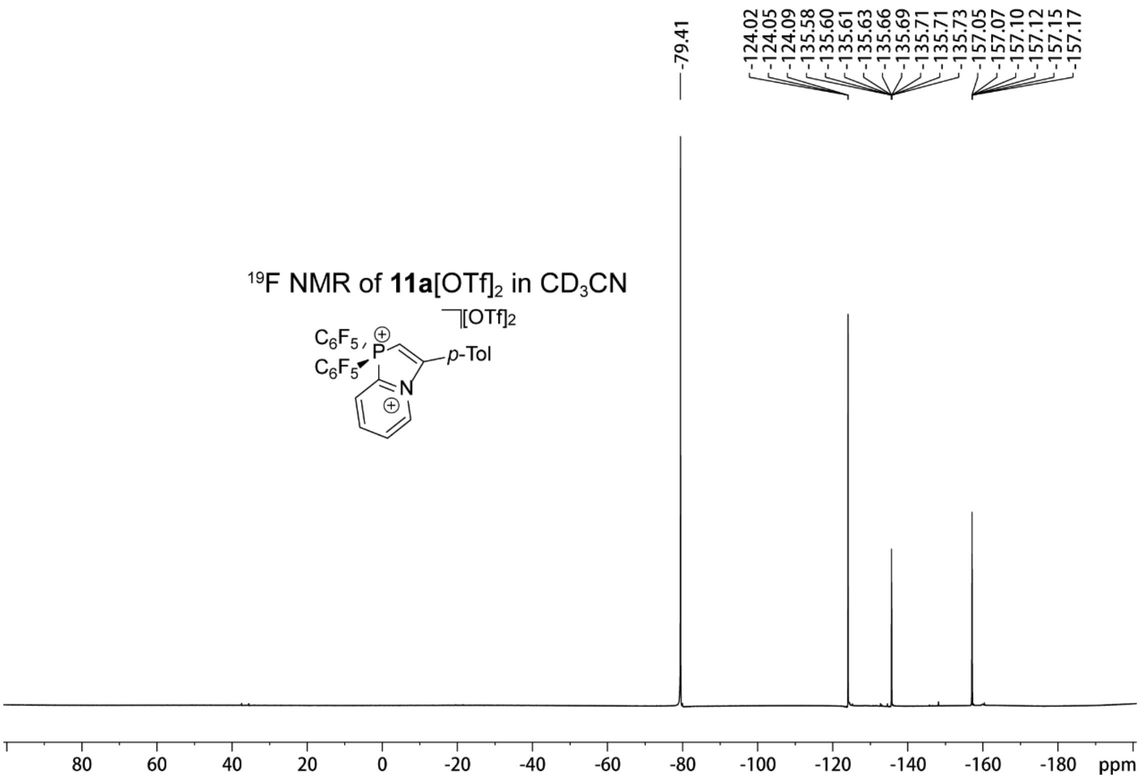

Figure **S58.** ^{19}F NMR spectrum of **11a**[OTf]$_2$ (CD_3CN, 300 K).

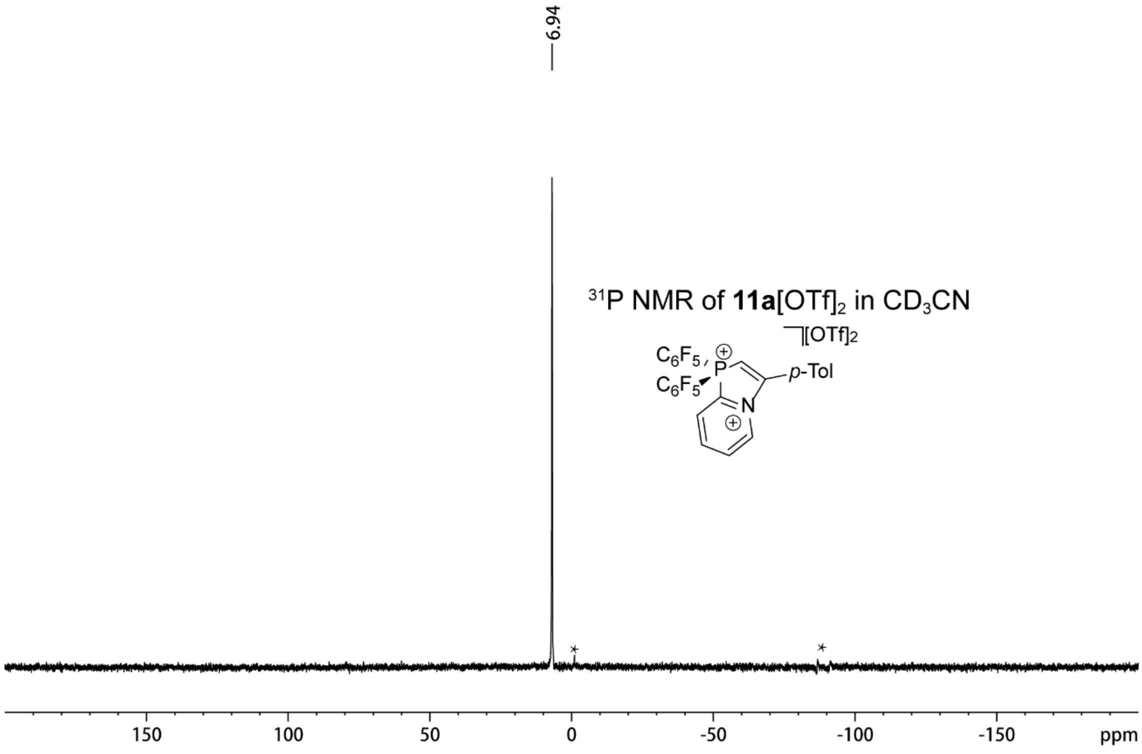

Figure **S59.** ^{31}P{H} NMR spectrum of **11a**[OTf]$_2$ (CD_3CN, 300 K); Asterisks indicate small amounts of unidentified side products.

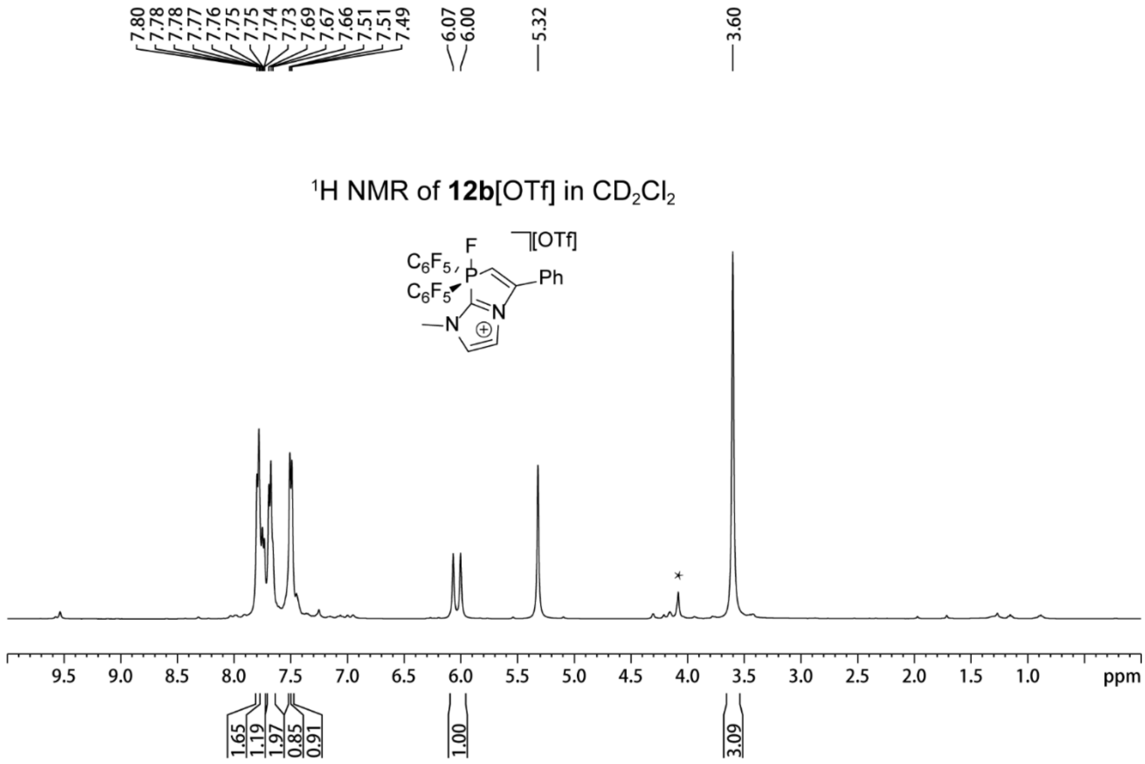

Figure **S60.** ${}^{1}H$ NMR spectrum of **12b**[OTf] (CD_2Cl_2, 300 K); Asterisks indicate small amounts of unidentified side products.

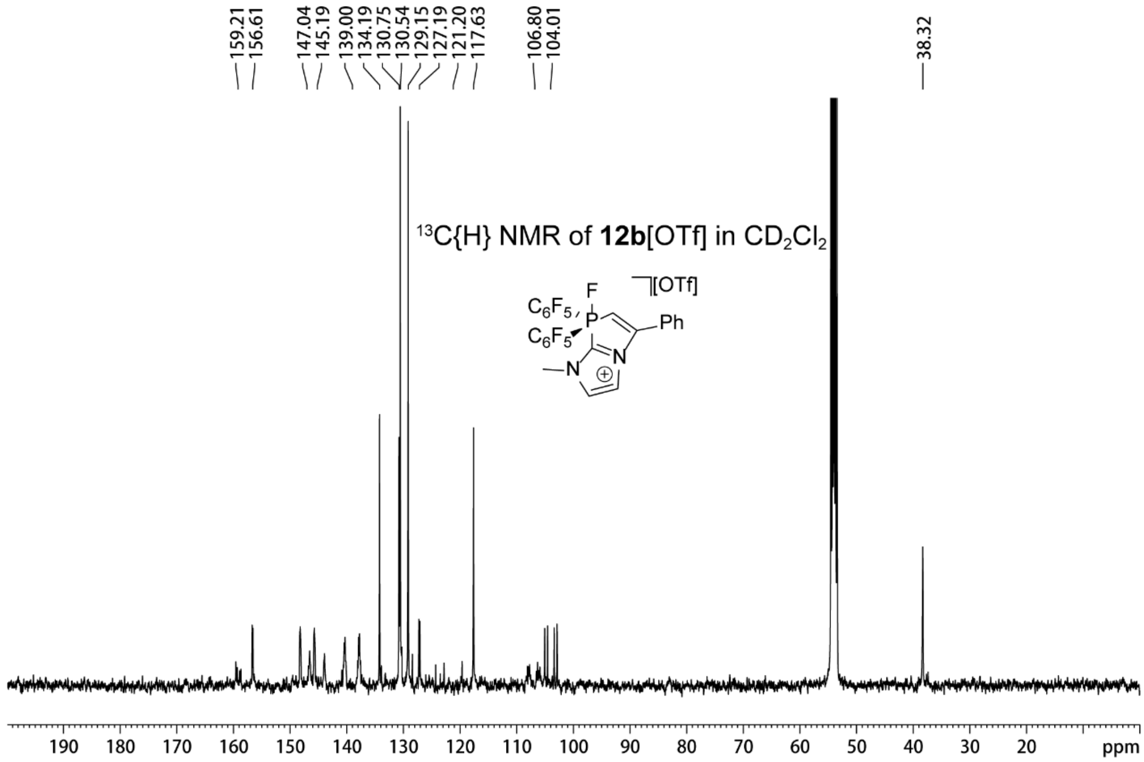

Figure **S61.** ${}^{13}C\{H\}$ NMR spectrum of **12b**[OTf] (CD_2Cl_2, 300 K).

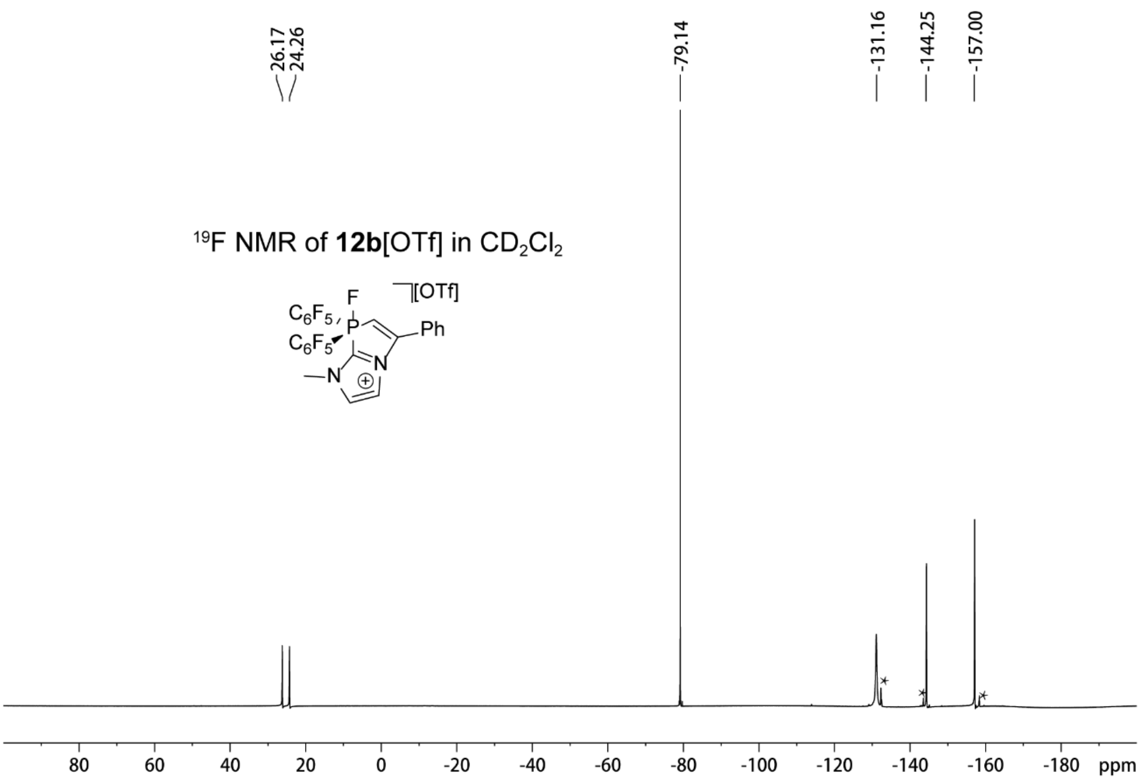

Figure **S62.** ^{19}F NMR spectrum of **12b**[OTf] (CD_2Cl_2, 300 K); Asterisks indicate small amounts of unidentified side products.

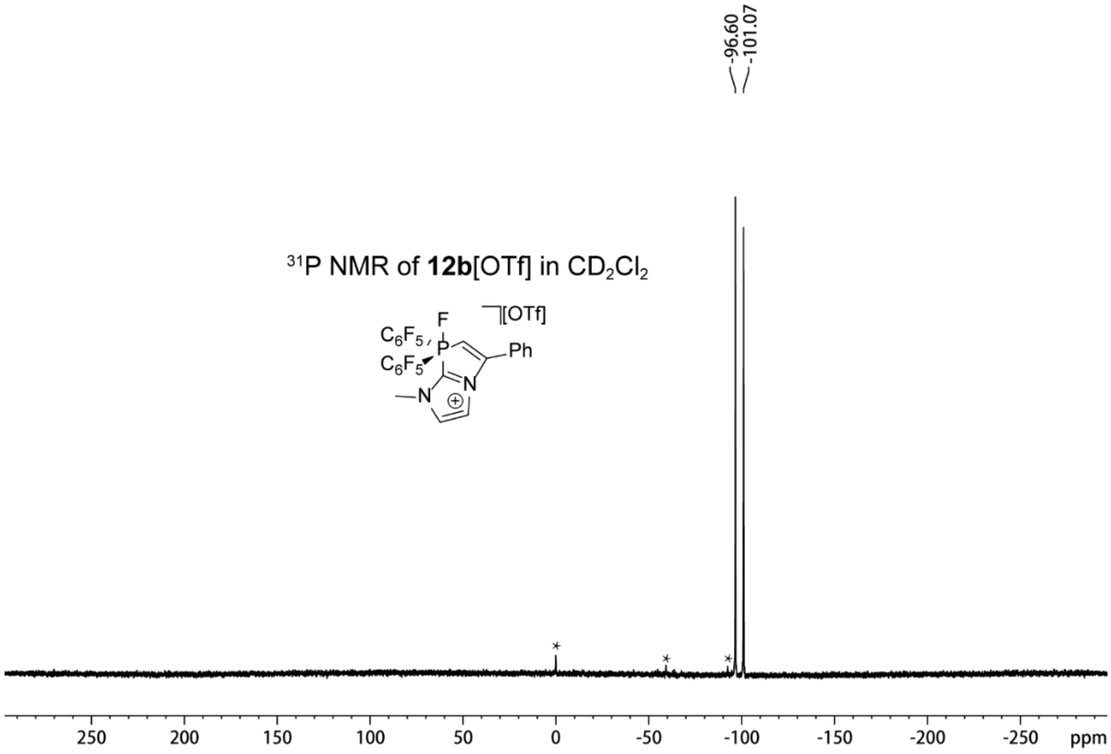

Figure **S63.** ^{31}P{H} NMR spectrum of **12b**[OTf] (CD_2Cl_2, 300 K); Asterisks indicate small amounts of unidentified side products.
